# Insertion, protonolysis and photolysis reactivity of a thorium monoalkyl amidinate complex†
†Electronic supplementary information (ESI) available: Experimental details, NMR spectra, and X-ray crystallographic tables. CCDC 1811106–1811116. For ESI and crystallographic data in CIF or other electronic format see DOI: 10.1039/c7sc05328b


**DOI:** 10.1039/c7sc05328b

**Published:** 2018-02-16

**Authors:** Nicholas S. Settineri, John Arnold

**Affiliations:** a Department of Chemistry , University of California , Berkeley , California 94720 , USA . Email: arnold@berkeley.edu; b Chemical Sciences Division , Lawrence Berkeley National Laboratory , Berkeley , California 94720 , USA

## Abstract

The reactivity of the thorium monoalkyl complex Th(CH_2_SiMe_3_)(BIMA)_3_ [**1**, BIMA = MeC(N^i^Pr)_2_] with various small molecules is described. While steric congestion prohibits the insertion of *N*,*N*′-diisopropylcarbodiimide into the Th–C bond in **1**, the first thorium tetrakis(amidinate) complex, Th(BIMA)_4_ (**2**), is synthesized *via* an alternative salt metathesis route. Insertion of *p*-tolyl azide leads to the triazenido complex Th[(*p*-tolyl)NNN(CH_2_SiMe_3_)-κ^2^N^1,2^](BIMA)_3_ (**3**), which then undergoes thermal decomposition to the amido species Th[(*p*-tolyl)N(SiMe_3_)](BIMA)_3_ (**4**). The reaction of **1** with 2,6-dimethylphenylisocyanide results in the thorium iminoacyl complex Th[η^2^-(C=N)-2,6-Me_2_-C_6_H_3_(CH_2_SiMe_3_)](BIMA)_3_ (**5**), while the reaction with isoelectronic CO leads to the products Th[OC(=CH_2_)SiMe_3_](BIMA)_3_ (**6**) and Th[OC(N^i^Pr)C(CH_2_SiMe_3_)(C(Me)N(^i^Pr))O-κ^2^O,O′](BIMA)_2_ (**7**), the latter being the result of CO coupling and insertion into an amidinate ligand. Protonolysis is achieved with several substrates, producing amido (**9**), aryloxide (**10**), phosphido (**11a,b**), acetylide (**12**), and cationic (**13**) complexes. Ligand exchange with 9-borabicyclo[3.3.1]nonane (9-BBN) results in formation of the thorium borohydride complex (BIMA)_3_Th(μ-H)_2_[B(C_8_H_14_)] (**14**). Complex **1** also reacts under photolytic conditions to eliminate SiMe_4_ and produce Th(BIMA)_2_(BIMA*) [**15**, BIMA* = (^i^Pr)NC(CH_2_)N(^i^Pr)], featuring a rare example of a dianionic amidinate ligand. Complexes **2**, **3**, **5**, **6**, **11a**, and **12–15** were characterized by ^1^H and ^13^C{^1^H} NMR spectroscopy, FTIR, EA, melting point and X-ray crystallography. All other complexes were identified by one or more of these spectroscopic techniques.

## Abstract 2

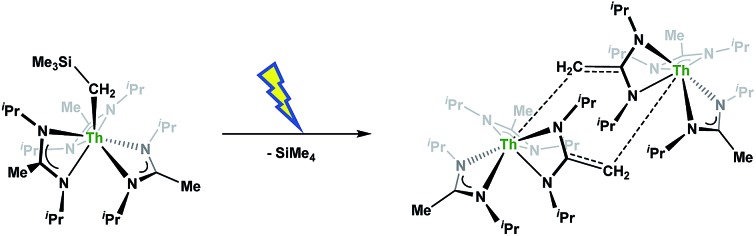
A tris-amidinate thorium monoalkyl complex facilitates new small molecule reactivity and eliminates SiMe_4_ under photolytic conditions to generate a mixed amidinate dimer.

## Introduction

The synthesis and reactivity of metal alkyl complexes have been a focus for organometallic chemists for decades due to their fundamental interest and relevance to catalytic and industrial processes. Studies of f-block alkyl complexes have been performed to a lesser extent, particularly those containing actinide metal centers. Actinide complexes display divergent coordination chemistry compared to the rest of the periodic table, and their large size combined with the accessibility of various oxidation states makes them particularly interesting for structural, electronic and reactivity studies.^1^–^6^ Much of the focus concerning organoactinide investigations has been devoted to uranium, due to its redox capabilities.^4^ Fewer studies have involved thorium, despite a fundamental question regarding whether thorium acts more as an actinide or group IV metal.^7^,^8^ Marks pioneered much of the work regarding thorium organometallic species bearing carbocyclic ancillary ligands (C_5_R_5_),^9^–^15^ and many groups have continued to explore the reactivity of these systems.^16^–^20^ Others have turned to non-carbocyclic ligands, in an attempt to investigate how complex stability and reactivity is affected by modifying the steric and electronic properties of the ancillary ligand framework.^21^–^28^ Having used a variety of non-carbocyclic ligand frameworks to stabilize and the explore the reactivity of both transition metal and actinide complexes,^29^–^34^ our group endeavored to expand these studies to thorium alkyl species. We recently reported on the synthesis of a thorium monoalkyl species utilizing a tris-amidinate ancillary framework, specifically Th(CH_2_SiMe_3_)(BIMA)_3_ (where BIMA = MeC(N^i^Pr)_2_) (**1**), and its ability to insert chalcogen atoms to generate rare thorium chalcogenolate complexes.^35^ In addition to the chalcogen insertion reactivity, the increased electrophilicity of the metal center with respect to analogous Cp-based systems led to the rare C–H activation of trimethylamine N-oxide. Inspired by this result, we sought to investigate how the unique properties of this system would impact the reactivity of **1** with a variety of small molecules. Here we report on the reactivity of **1** with organic azides, isocyanide, CO, nitrile, 9-BBN, and various protic substrates, as well as the stability of **1** under photolytic conditions.

## Results and discussion

**Scheme 1:**
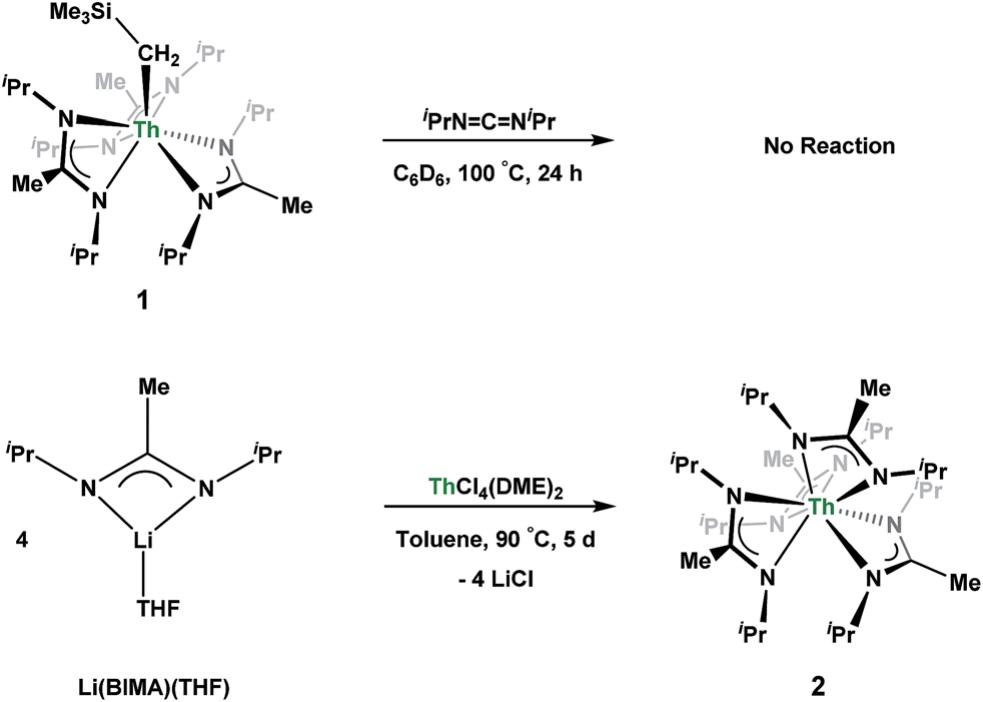
Attempted insertion and salt metathesis routes to tetrakis(amidinate) species.

**Fig. 1:**
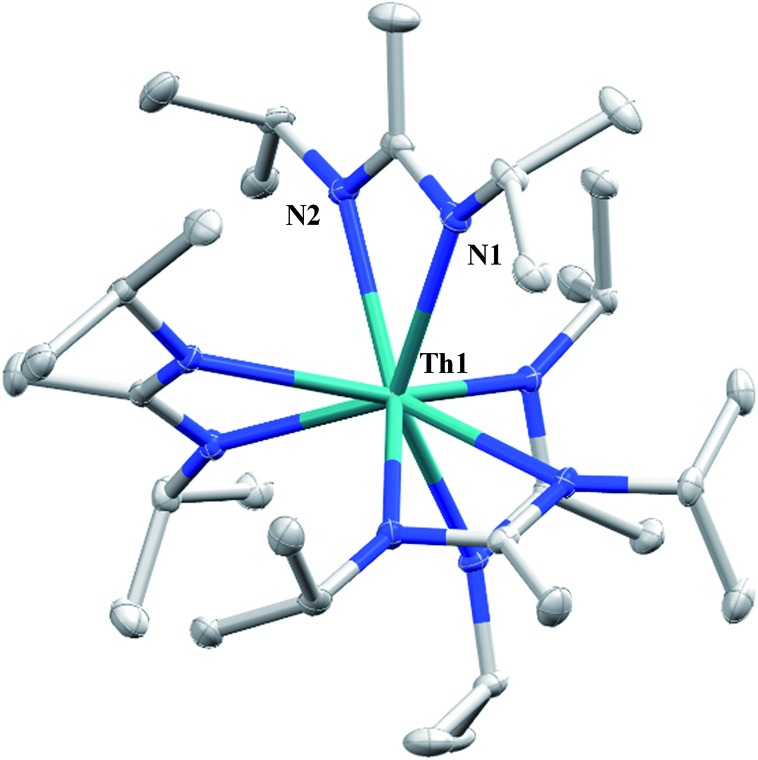
Molecular structure of **2** (thermal ellipsoids drawn at the 50% probability level). Hydrogen atoms omitted for clarity.

Having previously established the ability of **1** to undergo chalcogen atom insertion and generate unique thorium chalcogenolates,^35^ we next sought to examine potential insertion chemistry of this monoalkyl system with various small molecules. Evans has described the insertion of carbodiimides and organic azides into one alkyl moiety of 
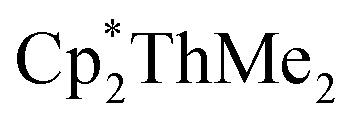
.^16^ With this in mind, we targeted the synthesis of a tetrakis(amidinate) complex through the reaction of **1** with *N*,*N*′-diisopropylcarbodiimide (Scheme 1). However, despite forcing conditions (100 °C), carbodiimide insertion into the Th–C bond was not observed, most likely a result of steric saturation around the metal center. Regardless, the alternative salt metathesis route using one equiv. of ThCl_4_(DME)_2_ (ref. 36) and 4.05 equiv. of Li(BIMA)(THF)^37^ and heating to 90 °C for 5 d afforded the desired homoleptic complex Th(BIMA)_4_ (**2**) as colourless crystals in 66% yield (Scheme 1). The ^1^H NMR spectrum of **2** reflects averaged *C*_4_-symmetry in solution, with one set of peaks observed for the equivalent amidinate ligands. The molecular structure of **2**, determined by single-crystal X-ray diffraction studies, shows a pseudo-tetrahedral geometry of the amidinate ligands around the thorium center (Fig. 1). The Th–N_amid_ bond lengths vary between 2.49 and 2.62 Å, a noticeably larger range than that seen in **1** (2.49–2.54 Å), indicating the significant steric congestion imposed by the isopropyl groups of the amidinate ligands. The structural parameters of **2** combined with the reaction conditions necessary to form **2** support the notion that insertion of carbodiimide into the Th–C bond was hindered by steric crowding, and indicate that related insertion reactions might be subject to this constraint. Due to the steric protection afforded by the tetrakis(amidinate) framework, and inspired by the work of Evans regarding low-valent thorium chemistry,^38^ we attempted the reduction of **2** with KC_8_ in the presence of 18-crown-6; nevertheless, no colour change was observed and only starting material was isolated.

**Scheme 2:**
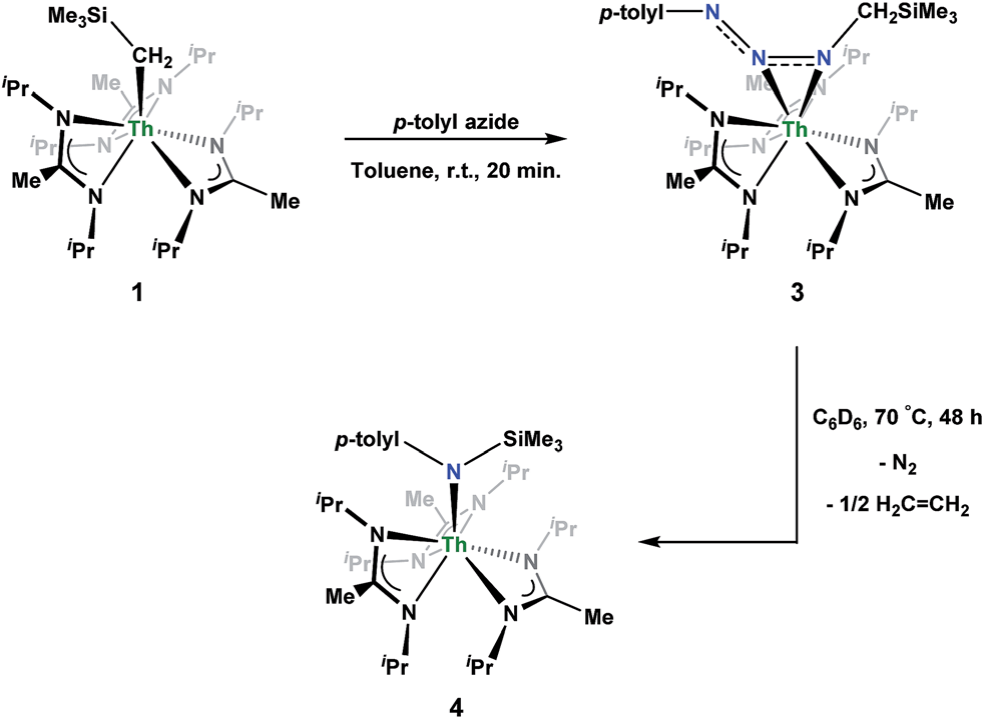
Synthesis of **3** and thermal decomposition to **4**.

**Fig. 2:**
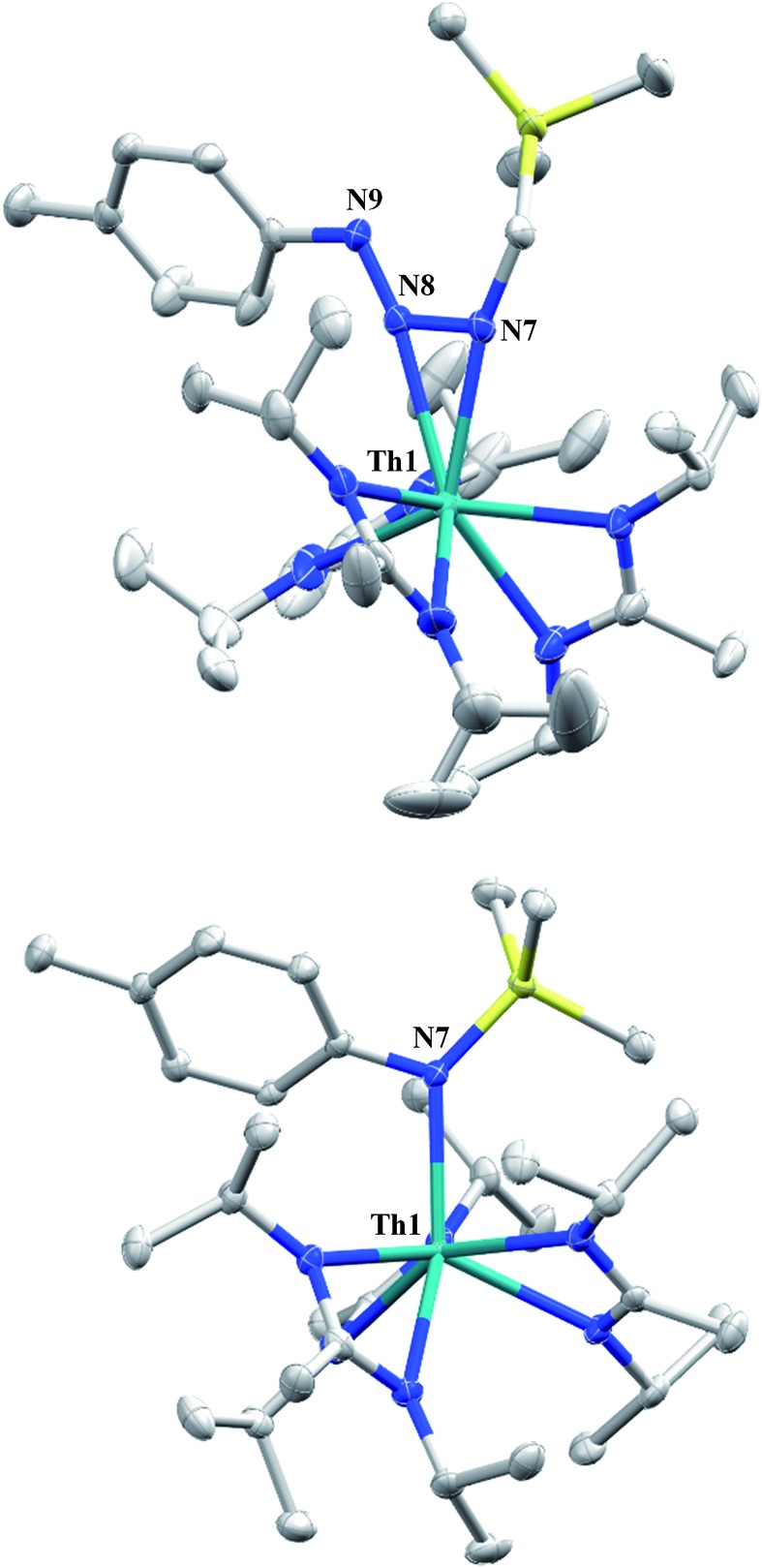
Molecular structures of **3** (top) and **4** (bottom) (thermal ellipsoids drawn at the 50% probability level). Hydrogen atoms omitted for clarity.

Reaction of **1** with an equivalent of *p*-tolyl azide resulted in insertion to form the triazenido complex Th[(*p*-tolyl)NNN(CH_2_SiMe_3_)-κ^2^N^1,2^](BIMA)_3_ (**3**) in 83% yield (Scheme 2). The ^1^H NMR spectrum displayed the diagnostic downfield shift of the methylene resonance which was also observed to result from chalcogen insertion.^35^ In the present case, we observed a shift from *δ* –0.08 in **1** to *δ* 3.99 in **3**, alongside shifted amidinate resonances and the appearance of resonances attributable to the *p*-tolyl group. X-ray diffraction studies revealed a κ^2^N^1,2^ coordination mode of the triazenido moiety (Fig. 2), similar to that observed by Evans in the thorium metallocene system.^16^ The metrical parameters relating to the N_3_ fragment show the effect of delocalization, with bond lengths of 1.314(6) and 1.285(6) Å for N(7)–N(8) and N(8)–N(9), respectively, a difference of only ∼0.03 Å. In contrast, Evans' system that utilized adamantyl azide exhibited a more localized bonding for the analogous nitrogens, with bond lengths of 1.360(3) and 1.243(3) Å, a difference of ∼0.12 Å.^16^ The Th(1)–N(7) distance of 2.474(4) Å is ∼0.1 Å longer than that seen in the thorium metallocene system, while the Th(1)–N(8) distance of 2.597(4) Å is the same in both. This indicates that the Th(1)–N(7) interaction is best described as anionic, whereas Th(1)–N(8) is more dative. The difference between **3** and Evans' metallocene system is likely a combination of both steric and electronic effects. Insertion with *tert*-butyl azide was also achieved; however, a mixture of two species was always observed, with the major product slowly converting to the minor product in solution until a ratio of ∼4 : 1 was established. Heating did not alter this ratio, although elevated temperatures (100 °C) induced decomposition of the products. We postulate that the two products are both triazenido complexes that differ only in their coordination mode, with the κ^2^N^1,2^ and κ^2^N^1,3^ species present in solution. Exposing **3** to elevated temperatures in solution to see if a similar change in coordination mode would occur brought about a different result; **3** undergoes clean thermal decomposition to a new complex, with significantly shifted *p*-tolyl aromatic resonances and, most notably, no methylene resonance. Complete conversion was achieved within 48 h while heating at 70 °C. This new product was stable to further heating. Close inspection of the ^1^H NMR spectrum revealed a resonance attributable to ethylene. With this information we envisioned that **3** was losing diazomethane (N_2_CH_2_), which then decomposed to dinitrogen and ethylene,^39^ resulting in the thorium amido species Th[(*p*-tolyl)N(SiMe_3_)](BIMA)_3_ (**4**, Scheme 2). Ethylene formation may be the result of singlet methylene^40^ generation and coupling upon N_2_CH_2_ decomposition. In order to try and trap the transient singlet methylene, similar heating experiments were conducted in the presence of 2-butyne and 1,1-diphenylethylene and monitored by ^1^H NMR spectroscopy. Although it was difficult to unambiguously identify the trapped products (1,2-dimethylcyclopropene and 1,1-diphenylcyclopropane, respectively), the formation of ethylene was not seen with either trapping reagent, and a singlet at *δ* 1.13 was observed in the 1,1-diphenylethylene experiment, which we tentatively assign to 1,1-diphenylcyclopropane.^41^ We were able to confirm the identity of **4** as the thorium amido complex by X-ray diffraction studies (Fig. 2). To the best of our knowledge, this is the first example of clean thermal decomposition of an actinide triazenido complex to the corresponding amido species. Bart and co-workers have observed thermal instability in certain uranium triazenido species, which has led to intractable mixtures of products.^42^ However, the thermal decomposition of L^*t*Bu^Fe(η^2^-HNNNAd) (where L^*t*Bu^ = *tert*-butyl substituted-*N*,*N*′-diaryl-β-diketiminate, aryl = 2,6-^i^Pr_2_-C_6_H_3_) to the corresponding primary amido species L^*t*Bu^FeNHAd has been observed.^43^ This was rationalized based on the instability of free H_2_NNNR compounds with respect to loss of dinitrogen. The Th(1)–N(7) bond distance of 2.399(2) Å in **4** is very close to the Th–N bond distance of 2.389(2) Å observed by Walter and co-workers in [η^5^-1,2,4-(Me_3_C)_3_C_5_H_2_]_2_Th(Cl)-[N(*p*-tolyl)SiH_2_Ph], which has a similar silyl amide environment.^44^ The nitrogen atom of the amido exhibits a trigonal planar geometry (Σ∠ ≈ 360°), also consistent with Walter's complex. A series of NMR scale experiments revealed the thermal decomposition to be concentration dependent, with higher concentrations of **3** leading to the generation of the silyl amine (*p*-tolyl)NH(SiMe_3_) (as determined by ^1^H NMR spectroscopy) and a mixture of unknown species (see Fig. S7 in ESI†). The identity of these products, along with mechanistic studies regarding the formation of **4** from **3**, is currently under investigation.

**Scheme 3:**
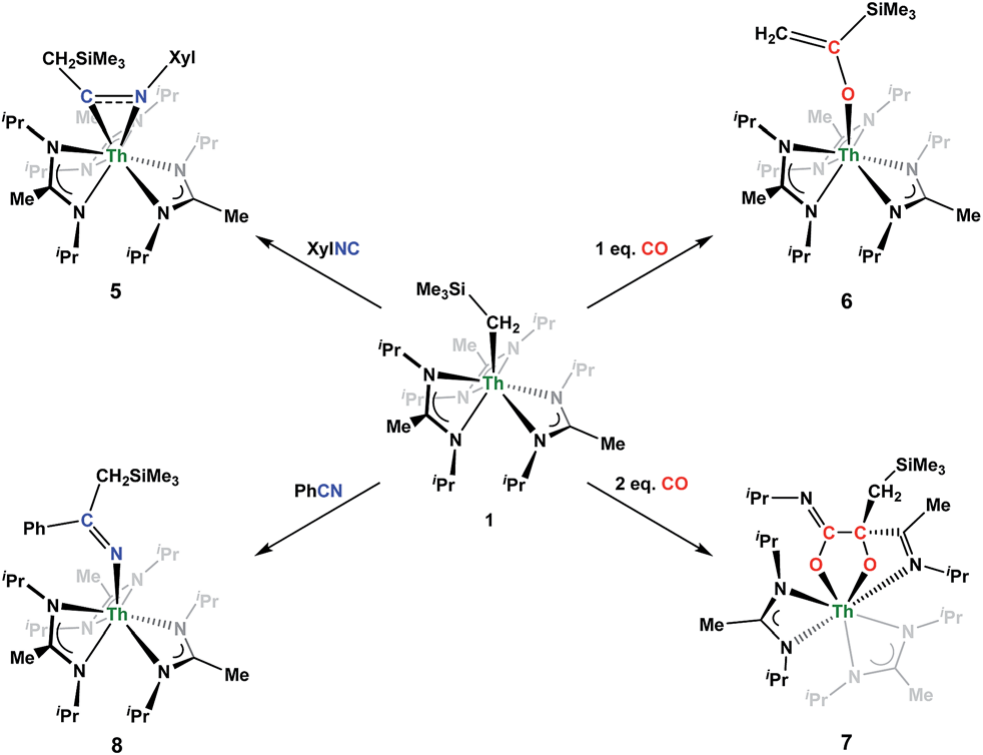
Insertion reactivity of **1**.

**Fig. 3:**
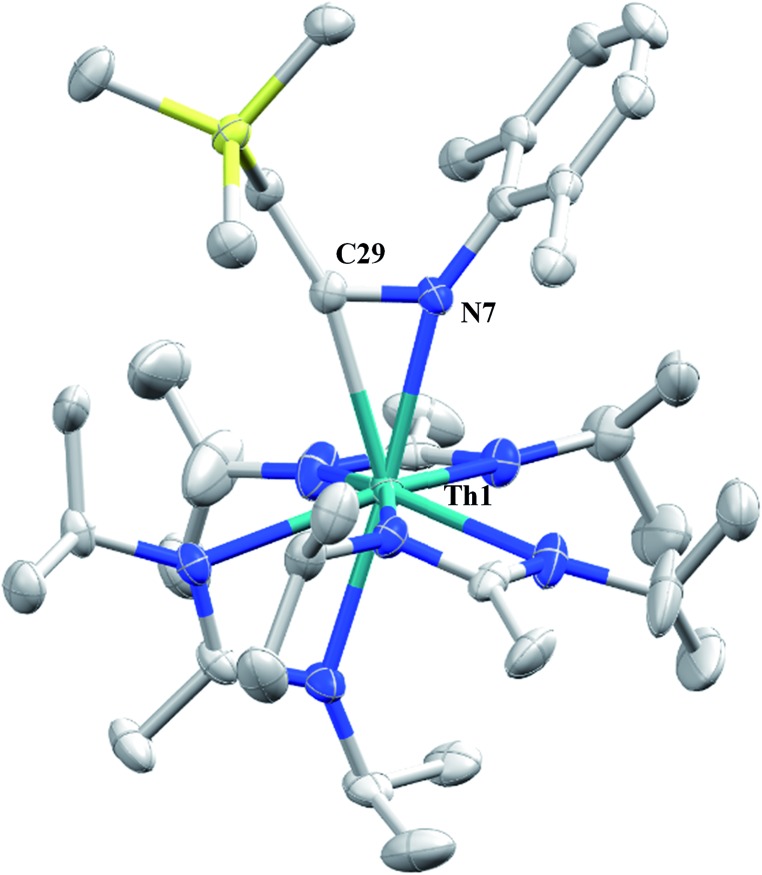
Molecular structure of **5** (thermal ellipsoids drawn at the 50% probability level). Hydrogen atoms omitted for clarity.

**Fig. 4:**
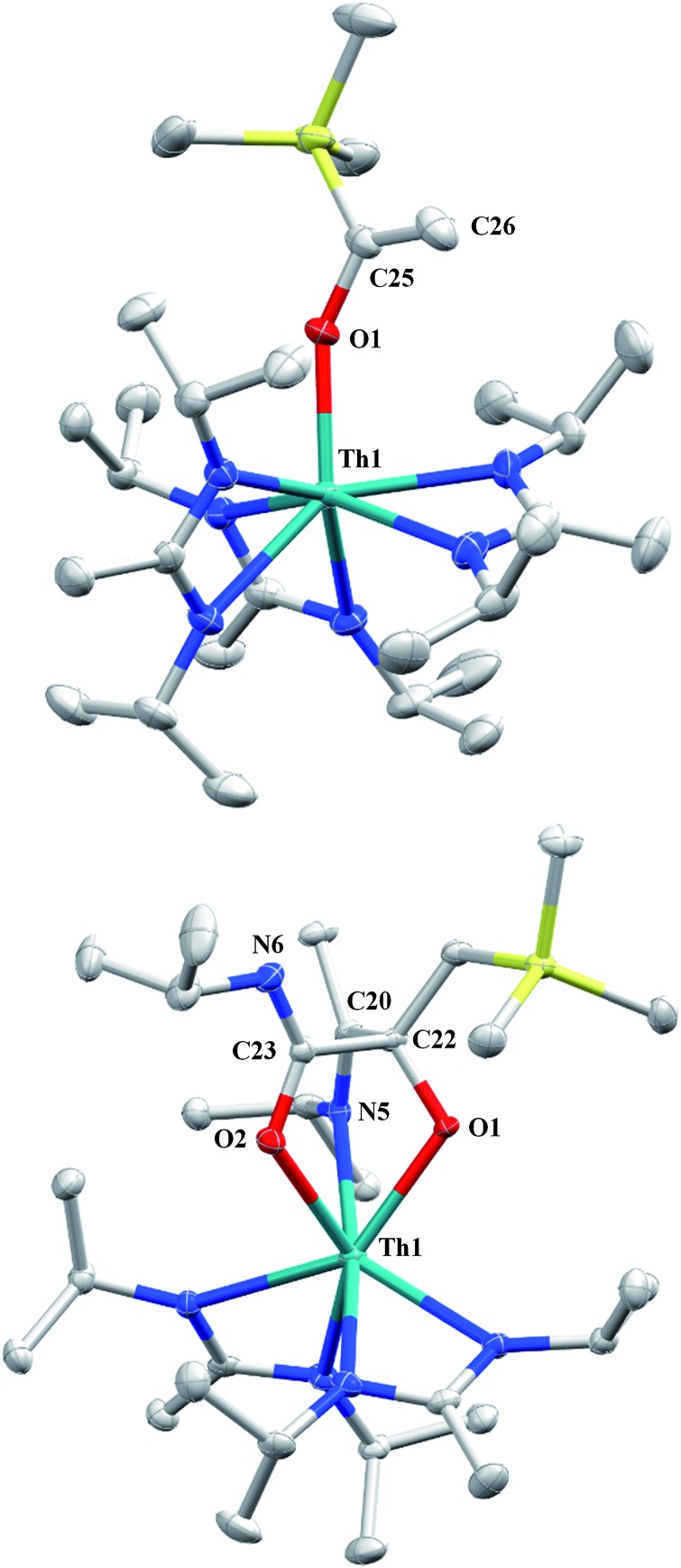
Molecular structures of **6** (top) and **7** (bottom) (thermal ellipsoids drawn at the 50% probability level). Hydrogen atoms omitted for clarity.

**Scheme 4:**
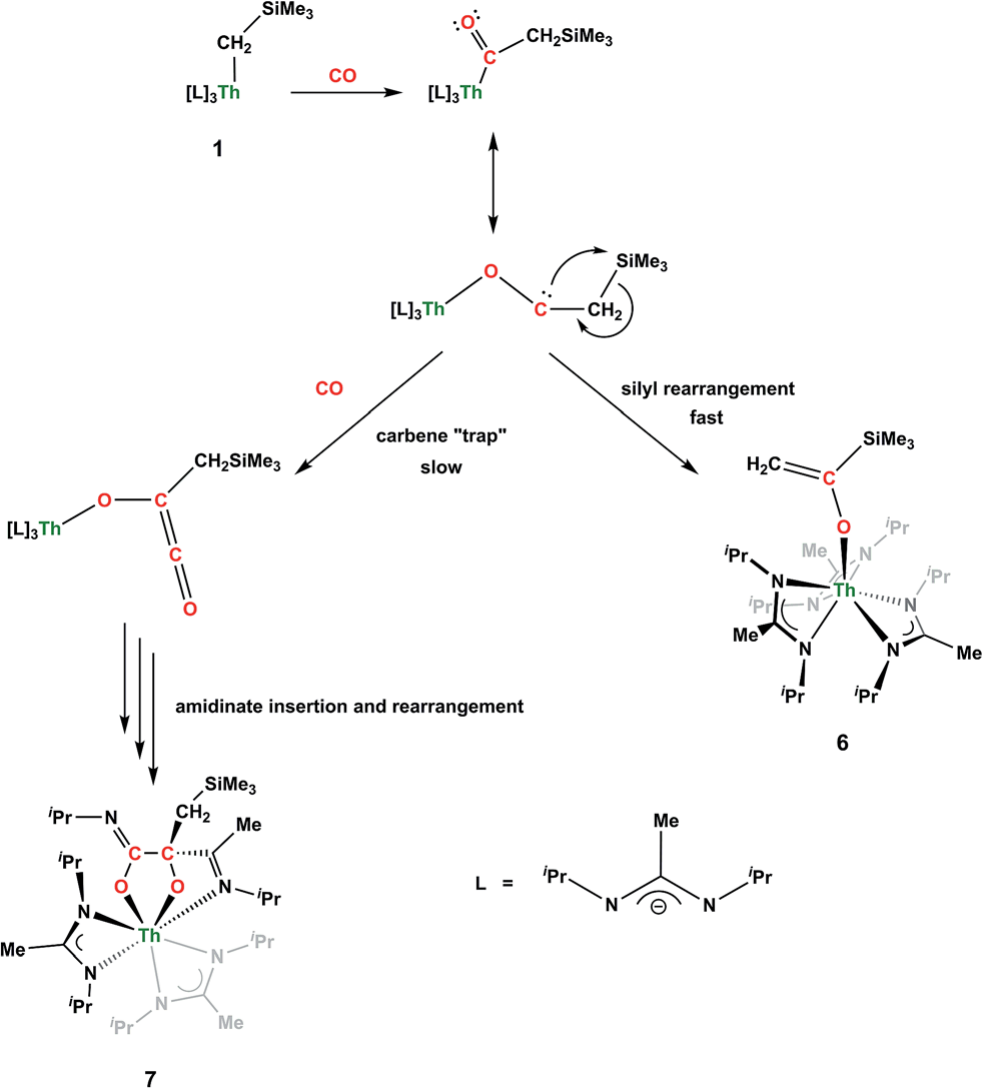
Proposed pathways for the formation of **6** and **7**.

Although achieved with both transition metal^45^–^48^ and uranium^49^–^52^ species, isocyanide insertion into thorium alkyl bonds to form the corresponding η^2^-iminoacyl complexes has not yet been reported. While sterics precluded the insertion of *N*,*N*′-diisopropylcarbodiimide, isocyanide insertion was realized with one equivalent of 2,6-dimethylphenylisocyanide and moderate heating, resulting in Th[η^2^-(C=N)-2,6-Me_2_-C_6_H_3_(CH_2_SiMe_3_)](BIMA)_3_ (**5**) as a colourless, crystalline solid in 81% yield (Scheme 3). To the best of our knowledge this is the first thorium η^2^-iminoacyl complex. Monitoring the reaction by ^1^H NMR spectroscopy confirms that this insertion proceeds slowly at room temperature (>95% conversion after 96 h), presumably due to the steric clash between the xylyl moiety and amidinate ligands. As expected, the diagnostic downfield shift of the methylene singlet to *δ* 2.85 indicated successful isocyanide insertion, along with new methyl and aromatic resonances corresponding to the xylyl group. X-ray diffraction studies revealed the η^2^-coordination mode of the imine moiety, with the N(7)–C(29) bond length of 1.299(4) Å falling in the range seen for other transition metal iminoacyl species (Fig. 3).^45^–^52^ The Th(1)–N(7) distance of 2.469(2) Å is noticeably shorter than that typically observed for a dative Th–N bond,^53^–^56^ while the Th(1)–C(29) bond length of 2.529(3) Å is in the range observed for other σ-bonded alkyl moieties.^14^,^19^,^57^ This insertion differs from that observed by Andersen and co-workers in their Th(*C*H_2_SiMe_2_*N*SiMe_3_)(NR_2_)_2_ (where R = SiMe_3_) complex, which undergoes insertion of *tert*-butyl isocyanide into the C–Si bond of the metallacycle to produce Th[(^*t*^Bu)*N*C(CH_2_)SiMe_2_*N*SiMe_3_](NR_2_)_2_.^58^ Similar reactivity was observed with CO in Andersen's system, resulting in Th[(*O*C(=CH_2_)SiMe_2_*N*SiMe_3_)](NR_2_)_2_. Exposing **1** to 1 atm of CO resulted in the formation of two products with similar solubilities in non-polar solvents, precluding their clean isolation and characterization, despite yields of ∼80% for the bulk mixture. The major species, as identified by ^1^H NMR spectroscopy, exhibited inequivalent methylene protons that are consistent with those observed in Th[(*O*C(=CH_2_)SiMe_2_*N*SiMe_3_)](NR_2_)_2_ (see Fig. S10 in ESI†). This inequivalency would not be seen in the ^1^H NMR spectrum of the thorium acyl species generated by simple CO insertion into the Th–C bond; thus, we postulated that it was likely a similar insertion into the C–Si bond of **1** occurred (Scheme 3). This hypothesis was proven by X-ray diffraction studies, as a few X-ray quality crystals were isolated from a very concentrated pentane solution stored at –35 °C for 3 days, confirming the identity of the CO insertion as the enolate complex Th[OC(=CH_2_)SiMe_3_](BIMA)_3_ (**6**) (Fig. 4). Complex **6** crystallized with two independent molecules in the asymmetric unit due to disorder in the enolate moiety; thus, the metrical parameters of only the nondisordered molecule will be discussed. The Th(1)–O(1) bond length of 2.216(2) Å is slightly longer than the Th–O bond length of 2.166(2) Å seen in Th(OCH_2_NMe_2_)(BIMA)_3_,^35^ while the C(25)–C(26) bond length of 1.338(5) Å is consistent with a carbon–carbon double bond, and the trigonal planar geometry of C25 (Σ∠ ≈ 360°) indicates sp^2^ hybridization. This type of reactivity has precedent in both uranium and thorium systems,^52^,^58^–^61^ and has been explained by initial CO insertion into the M–C bond to form the metal acyl, which then isomerizes to form a carbene-like intermediate that can then insert into the Si–C bond (Scheme 4). Alongside crystals of **6** were crystals of a different product, which we have tentatively assigned to the other resonances observed in the ^1^H NMR spectrum of the bulk material (see Fig. S10 in ESI†). X-ray diffraction studies revealed this product to be Th[OC(N^i^Pr)C(CH_2_SiMe_3_)(C(Me)N(^i^Pr))O-κ^2^O,O′](BIMA)_2_ (**7**), the result of reductive CO coupling and insertion into an amidinate ligand (Fig. 4). The Th(1)–O(1) and Th(1)–O(2) bond lengths of 2.220(2) and 2.290(2) Å, respectively, are slightly longer than that seen in **6** and Th(OCH_2_NMe_2_)(BIMA)_3_,^35^ while the bond length of 2.764(3) Å for Th(1)–N(5) is indicative of a dative interaction.^53^–^56^ A single bond length of 1.558(5) Å is observed for C(22)–C(23), whereas imine bonds are seen for C(23)–N(6) and C(20)–N(5) (1.266(4) and 1.282(4) Å, respectively). This type of CO coupling mimics the enediolate formation observed with various actinide bis-alkyl systems.^59^,^62^ Regarding the formation of **7**, it seems unlikely that this product is obtained from the interaction of a molecule of CO with **6**, due to the intact nature of the trimethylsilylmethyl alkyl fragment. Instead, it is likely that **7** results from “trapping” of the carbene-like intermediate, which precedes formation of **6**, by another molecule of CO to form a transient ketene,^63^ and subsequent insertion and rearrangement steps lead to **7** as the final product (Scheme 4). Kinetically this intermolecular process is slower than the intramolecular attack by the carbene intermediate on the C–Si bond, producing **6** as the major product. Exposing a mixture of **6** and **7** to additional CO did not change the ratio of products observed, confirming that **7** is not generated from **6**. In an attempt to avoid the formation of **7**, the slow addition of 1 eq. of CO to a stirred hexanes solution of **1** was conducted, resulting in clean formation of **6** in 64% yield. Development of a synthetic strategy to produce **7** is currently underway. Looking to other small molecules, insertion reactivity with CO_2_ and CS_2_ did not proceed cleanly, yielding intractable mixtures.

Nitrile insertion was examined by the NMR-scale reaction of **1** and benzonitrile. The reaction was sufficiently complete (>95% conversion by ^1^H NMR spectroscopy) upon heating to 100 °C for 72 h, resulting in the ketimide complex Th[–N=C(Ph)(CH_2_SiMe_3_)](BIMA)_3_ (**8**) (Scheme 3). The methylene singlet is seen downfield at *δ* 2.73, along with new resonances corresponding to the aromatic protons of the phenyl ring, as well as aromatic resonances corresponding to the cyclotrimerization product of benzonitrile, 2,4,6-triphenyl-1,3,5-triazine (see Fig. S13 in ESI†).^64^ The Lewis acid-catalysed cyclotrimerization of benzonitrile has been previously reported for lanthanide-imido species,^64^ although the active species in this transformation has not been identified.

**Scheme 5:**
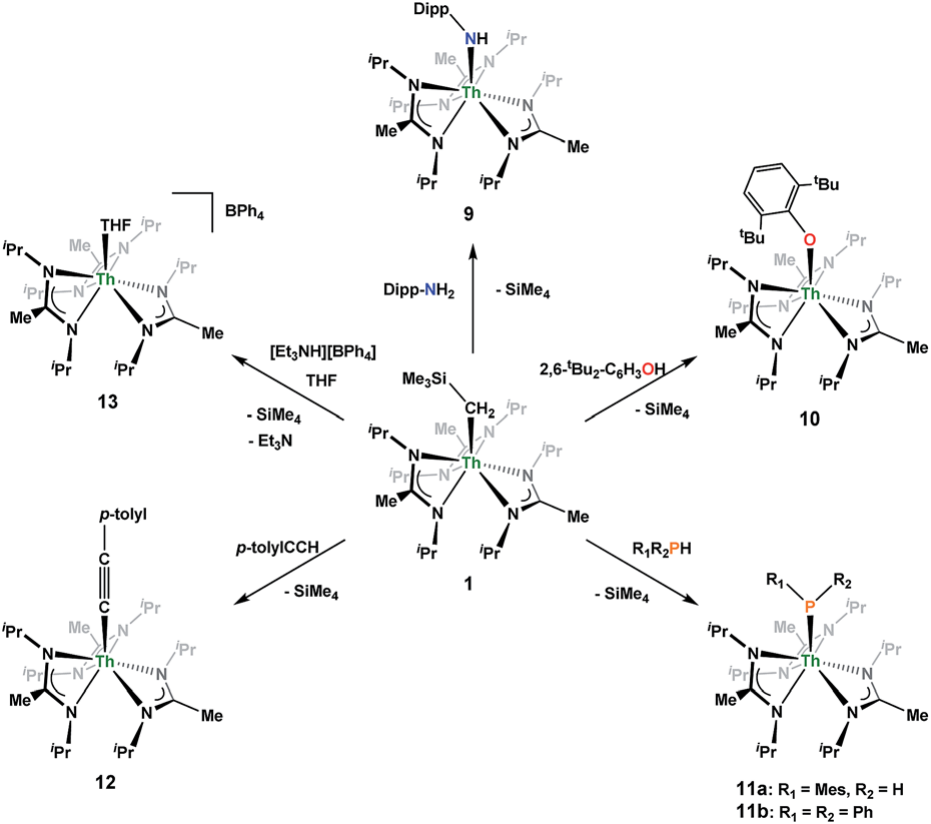
Protonolysis reactivity of **1**.

Looking to exploit the basic nature of the alkyl moiety of **1**, protonolysis reactivity was explored with a variety of protic substrates, the results of which are summarized in Scheme 5. NMR-scale experiments were carried out with **1** and 2,6-diisopropylaniline, resulting in the primary amido species Th(NH-2,6-^i^Pr_2_-C_6_H_3_)(BIMA)_3_ (**9**), as well as **1** and 2,6-di-*tert*-butylphenol, resulting in the aryloxide complex Th(O-2,6-^*t*^Bu_2_-C_6_H_3_)(BIMA)_3_ (**10**), as determined by ^1^H NMR spectroscopy. While phenol addition and subsequent elimination of SiMe_4_ occurred within 12 h at room temperature, deprotonation of the aniline required extended reaction times at elevated temperatures before conversion to **9** was achieved. This can be rationalized on the basis of the higher p*K*_a_ of the aniline (∼30 *vs.* 16.8 in DMSO),^65^,^66^ as the sterics imposed by the 2,6-di-*tert*-butylphenol are greater than that of 2,6-diisopropylaniline.

**Fig. 5:**
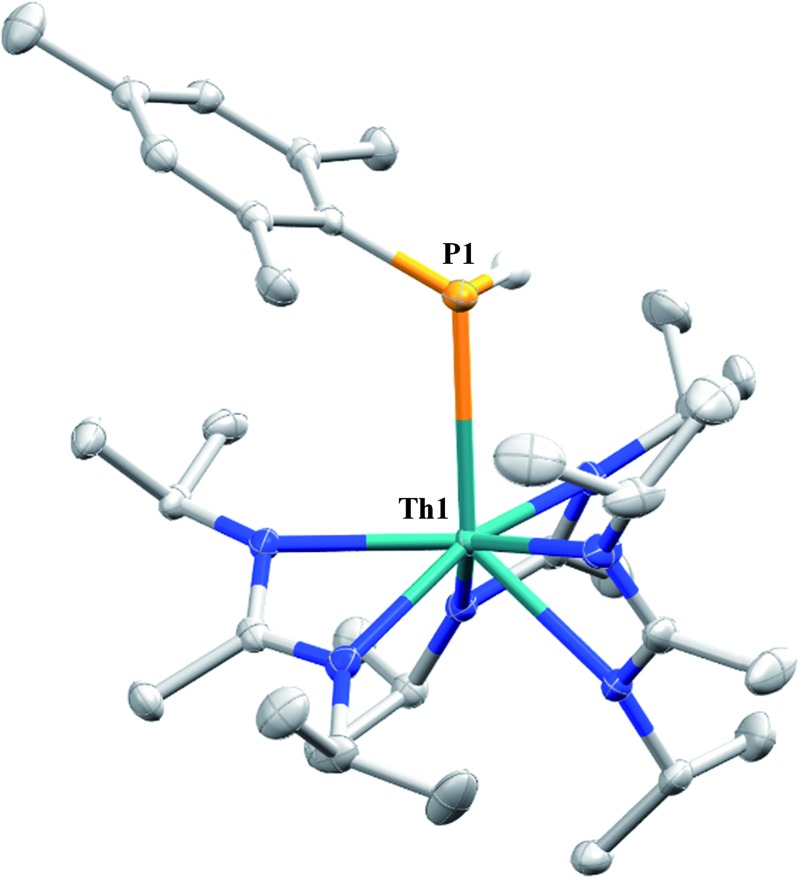
Molecular structure of **11a** (thermal ellipsoids drawn at the 50% probability level). Non-phosphorus-bound hydrogen atoms omitted for clarity.

Similar NMR experiments were also performed with primary and secondary phosphines, namely mesitylphosphine and diphenylphosphine, resulting in the thorium phosphido complexes Th(PHMes)(BIMA)_3_ (**11a**) (Mes = 2,4,6-trimethylphenyl) and Th(PPh_2_)(BIMA)_3_ (**11b**), respectively, as determined by ^1^H and ^31^P NMR spectroscopy. Complex **11a** exhibits a doublet at *δ* –45.4 in the ^31^P NMR spectrum with a ^1^*J*_P,H_ coupling constant of 195 Hz; the corresponding doublet in the ^1^H NMR is observed at *δ* 3.41. In the ^31^P NMR spectrum of **11b** a singlet is observed at *δ* 89.5 corresponding to the thorium phosphido, alongside peaks consistent with the dehydrocoupled product Ph_2_P–PPh_2_ (*δ* –14.9)^67^ and an unidentified phosphorus-containing species (*δ* 106). These ^31^P chemical shifts are in the range reported for other primary and secondary thorium phosphido species.^68^–^70^ The dehydrocoupling of phosphines has been reported with zirconium phosphido complexes under similar reaction conditions.^71^ Both complexes **11a** and **11b** required elevated temperatures and prolonged reaction times to reach completion. We sought a more scalable strategy to synthesize **11a** without the need for harsh reaction conditions, as complex **11a** has the potential to form a thorium phosphinidene *via* deprotonation of the phosphide ligand. With few examples of thorium phosphinidenes available, this would provide valuable information regarding metal–ligand multiple bonding between thorium and the heavier pnictogens.^72^–^74^ Salt metathesis between the previously reported ThCl(BIMA)_3_ (ref. 35) and KPHMes^70^ provided a more direct route to **11a** as bright yellow crystals in 58% yield. The ^13^C{^1^H} NMR spectrum of **11a** exhibits rare through-space coupling of the phosphorus atom and the isopropyl methyl carbons of the BIMA ligand (^TS^*J*_P,C_ = 2.1 Hz), as well as the *ortho*-methyls of the mesitylene ring (^TS^*J*_P,C_ = 9.6 Hz).^75^–^78^ This coupling is supported by the X-ray crystal structure of **11a**, which displays a close proximity of the phosphorus atom to one of the BIMA isopropyl methyls (3.746(4) and 3.779(4) Å) and mesityl methyls (3.062(3) and 3.070(3) Å), along with significant pyramidalization at P (Σ∠ ≈ 311°) in the two independent molecules found in the asymmetric unit (Fig. 5). The orientation of the phosphorus lone pair toward these carbon atoms facilitates this spin–spin interaction. This is the first crystallographically characterized example of a thorium monophosphido species bearing a primary phosphide ligand; to date, only a handful of primary bis(phosphido)-thorium species have been isolated and characterized.^70^,^72^,^79^ The Th–P bond lengths seen in the two molecules in the asymmetric unit (3.0497(8) and 3.0404(8) Å) are ∼0.15 Å longer than those observed in the previously reported bis(phosphido)-thorium complexes. Attempts to deprotonate **11a** to form the corresponding thorium phosphinidene have thus far proven unsuccessful.

**Fig. 6:**
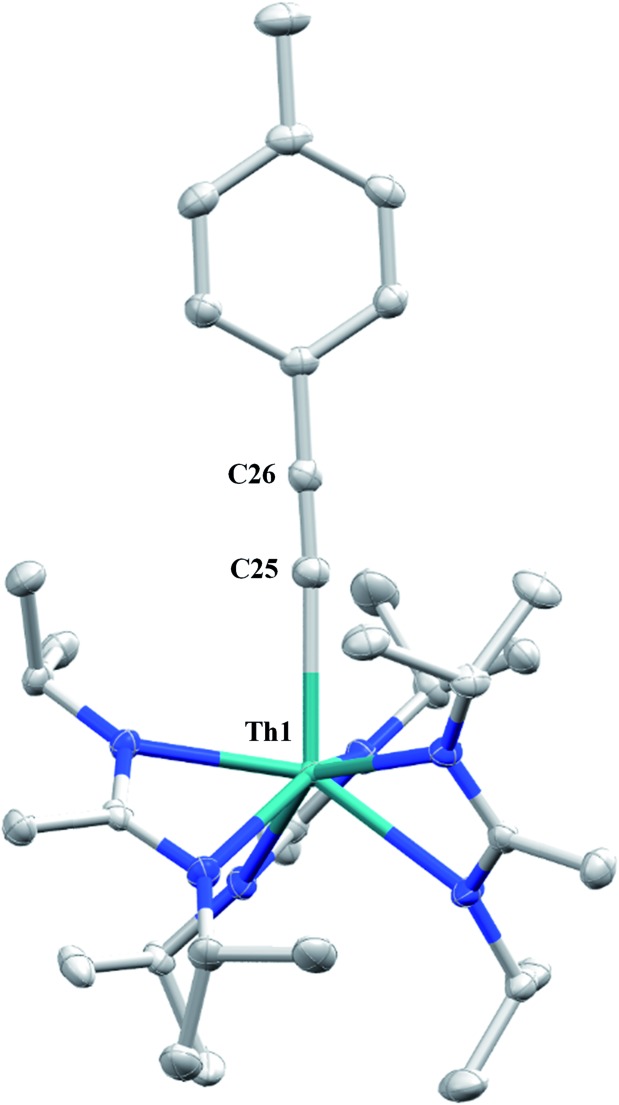
Molecular structure of **12** (thermal ellipsoids drawn at the 50% probability level). Hydrogen atoms omitted for clarity.

The reaction of **1** with *p*-tolylacetylene proceeds cleanly, providing the thorium acetylide complex Th(C≡C-*p*-tolyl)(BIMA)_3_ (**12**) as colourless crystals in 95% yield. This new alkynyl species may serve as a useful starting material for future chemistry, as other thorium acetylide complexes have been shown to be active catalysts for the linear oligomerization of terminal alkynes.^80^ The ^1^H NMR spectrum displays a *C*_3_-symmetric amidinate environment along with resonances attributable to the *p*-tolyl group, while the ^13^C{^1^H} NMR spectrum features a downfield resonance of *δ* 189.2 corresponding to the thorium-bound carbon atom of the alkyne, consistent with other thorium and group IV acetylides.^81^–^84^ The IR spectrum exhibits a characteristic signal at 2061 cm^–1^ assigned to the C≡C stretch. X-ray diffraction studies revealed a near-linear Th–C≡C bond angle of 175.7(2)° and bond lengths of 2.542(2) and 1.219(3) Å for Th(1)–C(25) and C(25)–C(26), respectively (Fig. 6). The Th–C bond length in **12** is ∼0.05 Å longer than that of the few other thorium acetylide species to have been characterized crystallographically, namely [(L)Th(C≡CSiMe_3_)_2_] and [(L)Th(C≡CSi^i^Pr_3_)_2_] (where L = *trans*-calix[2]benzene[2]pyrrolide), and Th(Bc^Mes^)_2_(C≡C-*p*-tolyl)_2_ (where Bc^Mes^ = mesityl-substituted bis(NHC)borate, NHC = N-heterocyclic carbene), which were only recently reported.^27^,^75^

**Fig. 7:**
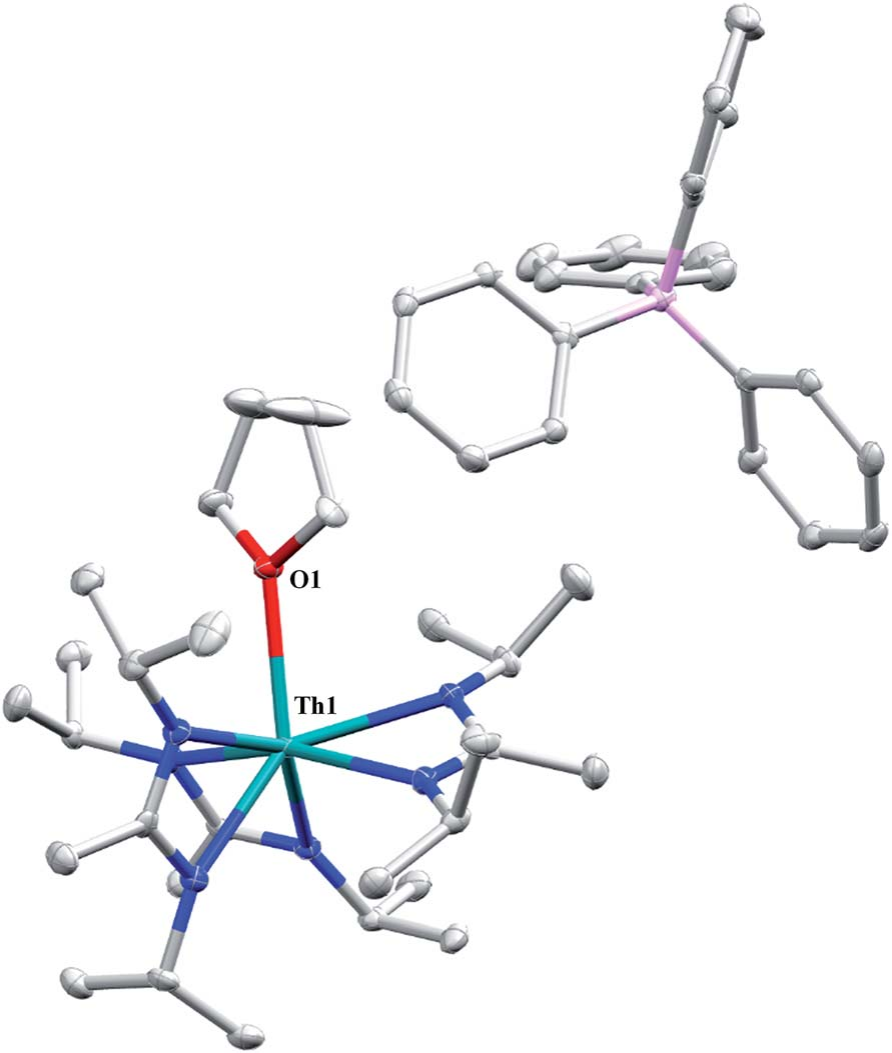
Molecular structure of **13** (thermal ellipsoids drawn at the 50% probability level). Hydrogen atoms and THF solvent molecule omitted for clarity.

A cationic species was targeted as a potential precursor to a Th(iii) amidinate complex, as Evans has shown that reduction of a mixed cyclopentadienyl amidinate thorium cation, namely {(C_5_Me_5_)_2_[^i^PrNC(Me)N^i^Pr]Th}{BPh_3_Me}, can be achieved with KC_8_.^85^ Treatment of **1** with [Et_3_NH][BPh_4_]^86^ in THF led to the isolation of [Th(THF)(BIMA)_3_][BPh_4_] (**13**) as colourless crystals in 81% yield. The NH_3_ and SiMe_4_ byproducts were easily removed under vacuum upon workup, and X-ray diffraction studies show a well separated ion pair with one THF molecule coordinated to the thorium center and another co-crystallized in the lattice (Fig. 7). The Th–N_amid_ bond lengths are noticeably shorter (2.45–2.48 Å) than that of **1** or **2**, likely due to reduced steric congestion and higher electrophilicity of the metal center. The Th(1)–O(1) distance of 2.504(2) Å is in the range observed for other THF-bonded thorium complexes.^87^–^90^ The second equivalent of THF can be removed under high vacuum. The ^1^H NMR spectrum of dried **13** in CDCl_3_ displays equivalent amidinate resonances, the aromatic peaks of the BPh_4_ anion, and one set of resonances corresponding to coordinated THF. The complex displays appreciable stability in CDCl_3_, but begins to decompose after ∼24 h at room temperature. Attempted reduction of **13** with KC_8_ in THF led to the isolation of **2**. No colour change was observed throughout the reaction. Increasing the sterics of the R groups on the amidinate nitrogens may help stabilize a tris-amidinate Th(iii) complex, and work is currently ongoing to test this hypothesis. Attempts to utilize H_2_ as a protic substrate and form a thorium hydride complex were not successful.

**Scheme 6:**
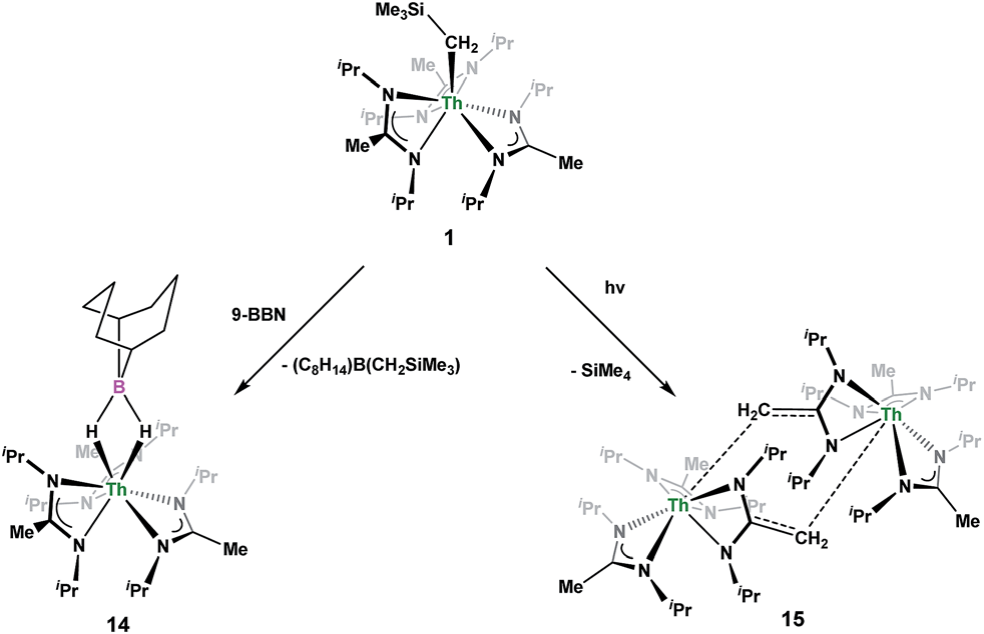
Ligand exchange and photolytic reactivity of **1**.

**Fig. 8:**
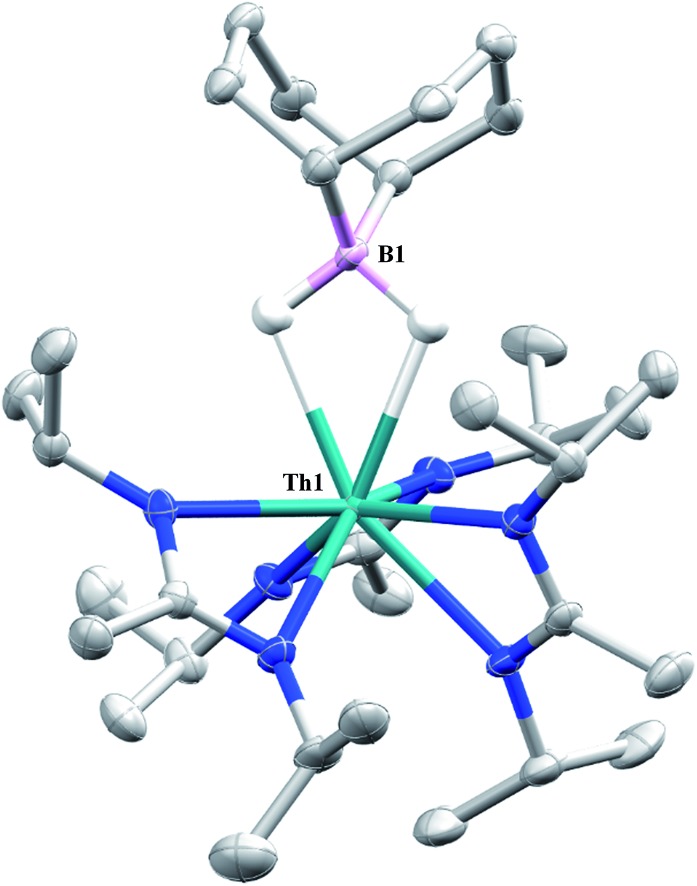
Molecular structure of **14** (thermal ellipsoids drawn at the 50% probability level). Non-hydride hydrogen atoms omitted for clarity.

Complex **1** undergoes ligand exchange with one equivalent of 9-borabicyclo[3.3.1]nonane (9-BBN), affording (BIMA)_3_Th(μ-H)_2_[B(C_8_H_14_)] (**14**) alongside one equivalent of (C_8_H_14_)B(CH_2_SiMe_3_), as determined by ^11^B{^1^H} NMR spectroscopy (resonance observed at *δ* 84.3 in C_6_D_6_).^91^ Complex **14** was isolated in 66% crystalline yield after workup (Scheme 6). The thorium borohydride complex exhibits equivalent amidinate ligands in solution according to ^1^H NMR spectroscopy. Additionally, broad μ-*H* resonances and several multiplets corresponding to the C_8_H_14_ fragment are observed. The ^11^B{^1^H} NMR spectrum exhibits a single resonance at *δ* 4.90, which is in the range typically observed for boron hydrides.^92^ FTIR spectroscopy reveals a broad B–H stretch centered at 2021 cm^–1^. X-ray diffraction studies show an eight-coordinate thorium center bearing bridging hydrides bound to the 9-BBN moiety (Fig. 8). The hydrides were located in the Fourier difference map and refined isotropically. The Th(1)–B(1) distance of 2.952(9) Å is significantly longer than typically observed with other thorium complexes containing bridging borohydrides (2.49(6)–2.670(2) Å),^93^–^97^ but is within the range observed by Girolami and co-workers in the complexes [Th(H_3_BNMe_2_BH_3_)_4_] and [Th(H_3_BNMe_2_BH_3_)_2_(BH_4_)_2_], which exhibit Th–B distances between 2.848(9) to 3.193(5) Å.^97^ Complex **14** is surprisingly stable both under photolytic and elevated temperature conditions, with no decomposition or elimination of H_2_ observed.

Storage of **1** at room temperature under dry nitrogen for several weeks led to slight discolouration of the crystalline solid. While the ^1^H NMR spectrum of this material showed very little change from that of pure **1**, we decided to investigate the stability of **1** under photolytic conditions. The UV-Vis spectrum of **1** features an absorption with *λ*_max_ = 295 nm, likely a ligand-based π–π* transition (see Fig. S31 in ESI†). Irradiation of **1** with UV-light centered at 253 nm in C_6_D_6_ and monitoring by ^1^H NMR spectroscopy showed elimination of SiMe_4_ alongside the production of a single new product, which displayed inequivalent amidinate ligands. Reaction times ranged from 24 h to 10 days, depending on the concentration of **1** in the sample (∼0.02–0.45 M), with higher concentrations taking longer. Optimization of the reaction conditions, by use of a quartz reaction vessel, cyclohexane-d_12_ and a xenon arc lamp, resulted in significantly reduced reaction times (∼2 h for ∼0.03 M solution). Removal of SiMe_4_ under vacuum and crystallization from toluene afforded Th(BIMA)_2_(BIMA*) (**15**) in 53% yield [BIMA* = (^i^Pr)NC(CH_2_)N(^i^Pr)]. Heating a solution of **1** at 100 °C for 24 h and monitoring by ^1^H NMR spectroscopy showed no decomposition of **1** or production of **15**, eliminating the possibility that conversion of **1** to **15** was thermally-induced.

**Fig. 9:**
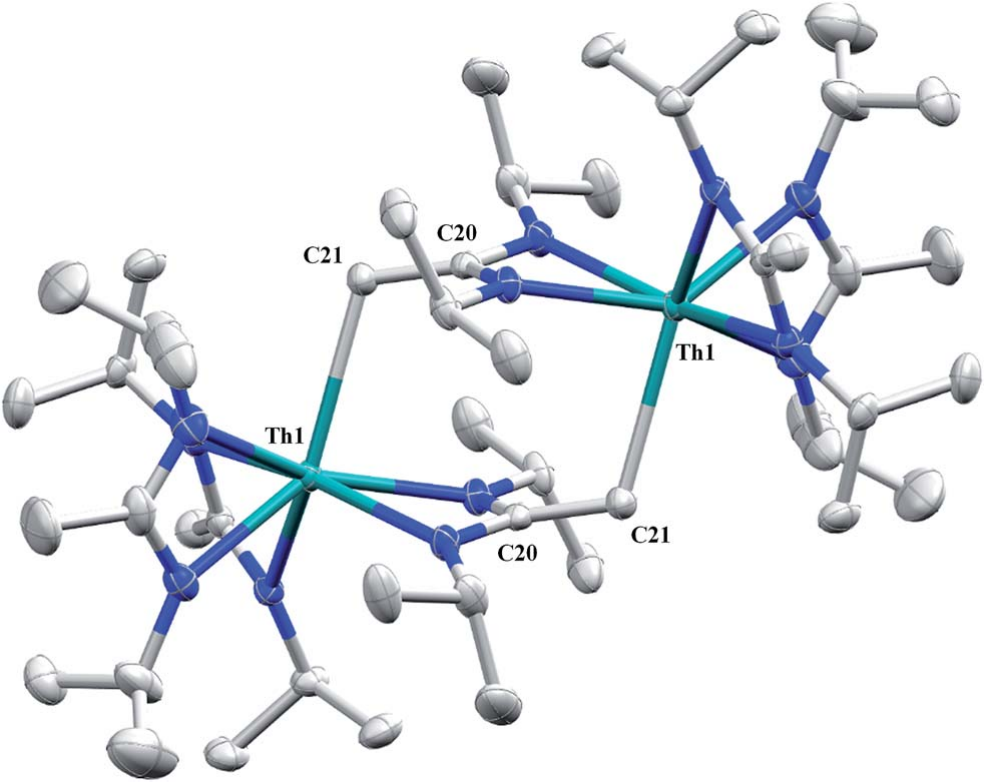
Molecular structure of **15** (thermal ellipsoids drawn at the 50% probability level).

Irradiation of **1** results in C–H activation of a methyl group on an amidinate ligand by the –CH_2_SiMe_3_ moiety, eliminating SiMe_4_ and reducing the activated amidinate to a dianionic ligand (Scheme 6). Heterolytic bond cleavage of the Th–C bond resulting in a –CH_2_SiMe_3_ anion, which then attacks the methyl backbone of an amidinate ligand, is a possible explanation for the C–H activation and subsequent ligand reduction observed. This is a rare example of an amidinate dianion,^64^ and the first generated under photolytic conditions. The ^1^H NMR spectrum of **15** exhibits a set of equivalent amidinate resonances, alongside the resonances attributable to the amidinate dianion, specifically a 2H septet at *δ* 4.24, a 2H singlet at *δ* 3.44, and a 12H doublet at *δ* 1.53. The 6H singlet corresponding to the methyl groups of the monoanionic amidinates also appears at *δ* 1.53 (see Fig. S29 in ESI†). In the ^13^C{^1^H} NMR spectrum the terminal methylene carbon resonance is observed noticeably downfield at *δ* 53.3, shifted by ∼40 ppm from the amidinate methyls seen at *δ* 12.2, but further upfield than that typically seen with alkenes. The ^1^*J*_C–H_ coupling constant of 157.6 Hz (as measured from the ^13^C satellites observed in the ^1^H NMR spectrum) is consistent with sp^2^ hybridization and similar to that observed for ethylene.^98^ The *ipso*-carbon of the amidinate dianion is shifted upfield to *δ* 154.2 from *δ* 172.8 as observed for the monoanionic amidinates. X-ray diffraction studies revealed a dimeric structure where the thorium centers are bridged by the methylene carbon of the dianionic amidinate ligand (Fig. 9). Complex **15** crystallizes in *P*1 with the asymmetric unit containing only the monomer unit; the dimer is generated through inversion symmetry. The Th(1)–C(21) bond distance of 2.749(3) Å is significantly longer than the Th–C bond observed in **1** (2.557(3) Å), but shorter than the long Th–σ-alkyl bond distance of 2.875(9) Å observed by Liddle and co-workers in [Th{N(CH_2_CH_2_NSiMe_2_^*t*^Bu)_2_(CH_2_CH_2_NSiMe^*t*^Bu-μ-CH_2_)}]_2_.^90^ The C(20)–C(21) bond distance of 1.438(4) is longer by ∼0.1 Å than typically observed for C–C double bonds, but this lengthening may be a result of delocalized electron density involved in the Th–C contact, reminiscent of a three-center two-electron bond. This is also manifested in the lack of planarity seen in the –N(CH_2_)CN– unit of the amidinate dianion.

## Conclusions

The amidinate-supported thorium monoalkyl complex **1** exhibits a variety of reactivity with small molecules, including insertion, protonolysis and photolysis. The insertion of *p*-tolyl azide lead to the thorium triazenido complex **3** which undergoes clean thermal decomposition at low concentrations to the corresponding amido complex **4**, through the loss of the unstable “N_2_CH_2_” fragment. This is the first example of an actinide complex undergoing this rare transformation. Insertion of xylyl isocyanide results in the first crystallographically characterized thorium iminoacyl complex. This insertion reactivity differs from that observed with CO, which instead results in the corresponding enolate species **6** upon CO insertion and rearrangement, as well as the unique double CO insertion and amidinate cleavage product **7**. The utility of the alkyl moiety as an internal base was demonstrated with a variety of protic substrates, with the thorium phosphido complex **11a** generated *via* both protonolysis and salt metathesis routes, the latter providing a more scalable option for the synthesis of **11a**. The photolytic elimination of SiMe_4_ concomitant with the reduction of an amidinate ligand to form complex **15** is unprecedented reactivity with amidinate-supported metal complexes, and a rare example of a complex bearing a dianionic amidinate ligand. Mechanistic and reactivity investigations of several of the complexes reported are currently ongoing.

## Conflicts of interest

There are no conflicts to declare.

## Supplementary Material

Supplementary information

Crystal structure data
