# Supplementary material for: Insertion, protonolysis and photolysis reactivity of a thorium monoalkyl amidinate complex
Source: Chem Sci. 2018 Feb 16;9(10):2831–41. doi: 10.1039/c7sc05328b (PMC5914426; doi:10.1039/c7sc05328b)
Supplement: Supplementary file 1 [file SC-009-C7SC05328B-s001.pdf]

Electronic Supplementary Material (ESI) for Chemical Science.
This journal is © The Royal Society of Chemistry 2018

Insertion, protonolysis and photolysis reactivity of a thorium monoalkyl amidinate complex

Nicholas S. Settineri and John Arnold*

Department of Chemistry, University of California, Berkeley, and Chemical Sciences Division, Lawrence Berkeley Laboratory, Berkeley, California, 94720, United States

E-mail: arnold@berkeley.edu

Supporting Information

A. Experimental Details S2-S8

B. NMR and UV-Vis Spectra S9-S39

C. X-ray Crystallographic Tables S40-S42

D. References S42

A. Experimental Details

General Considerations. Unless otherwise stated, all reactions were performed under an atmosphere of dry N_2 using standard Schlenk line techniques or in an MBraun N_2 atmosphere glovebox (<1.0 ppm of O_2/H_2O). All solvents were dried and degassed using a commercially available Phoenix SDS from JC Meyer Solvent Systems. All glassware, syringes, and cannulas were stored in a 140 °C oven for a minimum of 16 h prior to use. Deuterated solvents were vacuum-transferred from flasks containing sodium/benzophenone (C_6D_6, C_6D_{12}) or calcium hydride ($CDCl_3$), degassed with three freeze−pump−thaw cycles, and stored over molecular sieves. $ThCl_4(DME)_2$,[1] Li(BIMA)(THF),[2] $ThCl(BIMA)_3$,[3] $Th(CH_2SiMe_3)(BIMA)_3$ (**1**),[3] *p*-tolyl azide,[4] and KPHMes[5] were prepared according to previously reported literature procedures. 2,6-diisopropylaniline was purchased from SigmaAldrich and distilled prior to use. 2,6-di-*tert*-butylphenol was sublimed prior to use. Benzonitrile was dried over CaH_2 and distilled prior to use. CO gas was purchased from Praxair, Inc. and used directly from the cylinder without purification. All other reagents were purchased from commercial sources and used as received. Unless otherwise stated, NMR spectra were collected at ambient temperature on a Bruker AV-300, AVB-400, AV-500, or AV-600 spectrometer. ^1H and $^{13}C\{^1H\}$ NMR chemical shifts (δ) are reported in ppm and were calibrated to residual solvent peaks. $^{11}B\{^1H\}$ and ^{31}P NMR shifts (δ) are referenced to an external standard ($BF_3 \cdot Et_2O$ and H_3PO_4, respectively). Photo-activation reactions were conducted using a Rayonet reactor (model RPR-100) centered at 253 nm or a Newport 67005 Arc Lamp Housing containing a xenon arc lamp operating at 300 W. Melting points were determined on an Optimelt SRS instrument using capillary tubes sealed under dry N_2. Elemental analysis samples were sealed under vacuum and analyzed at either the London Metropolitan University or the University of California, Berkeley. Infrared spectra were collected on a Thermo Scientific Nicolet iS10 FTIR spectrophotometer using Nujol mulls pressed between KBr plates.

Synthesis of Th(BIMA)$_4$ (2): Solid $ThCl_4(DME)_2$ (2.49 g, 4.49 mmol) and Li(BIMA)(THF) (4.01 g, 18.2 mmol) were added to a 250 mL Schlenk flask containing a magnetic stir bar and suspended in toluene (60 mL). The suspension was then heated to 90 °C and stirred for 5 d, upon which the solution became slightly orange and contained a colourless precipitate. The volatiles were removed under reduced pressure, and the resulting solid was triturated with hexanes (2 x 30 mL), extracted into hexanes (2 x 50 mL), filtered through Celite, and concentrated to 15 mL. The solution was stored at -40 °C overnight, yielding **2** as analytically pure, colourless crystals (2.35 g, 65.6%). ^1H NMR (600 MHz, C_6D_6): δ 3.72 (sept, C*H*Me$_2$, 8H, $^3J_{H,H}$ = 6.6 Hz), 1.80 (s, NC(C*H*$_3$)N, 12H), 1.30 (d, CH*Me*$_2$, 48H, $^3J_{H,H}$ = 6.6 Hz). $^{13}C\{^1H\}$ (151 MHz, C_6D_6): δ 174.2 (N*C*N), 48.9 (*C*HMe$_2$), 25.3 (CH*Me*$_2$), 14.8 (NC(*C*H$_3$)N). Anal. Calcd for $C_{32}H_{68}N_8Th$ (797.0): C, 48.23; H, 8.60; N, 14.06. Found: C, 48.25; H, 8.71; N, 13.93. Mp: 150 °C (decomp.). FTIR (Nujol): 2595 (w), 1495 (s), 1358 (s), 1340 (s), 1310 (s), 1187 (s), 1175 (s), 1137 (m), 1118 (s), 1044 (m), 1007 (s), 943 (w), 798 (s), 722 (w), 618 (s), 577 (m), 542 (m). Crystals suitable for single-crystal X-ray diffraction studies were grown from a concentrated hexanes solution stored at -35 °C for 16 h.

Synthesis of Th[(*p*-tolyl)NNN(CH_2SiMe_3)-κ^{2}N^{1,2}](BIMA)$_3$ (3): Solid **1** (0.303 g, 0.408 mmol) was added to a 20 mL scintillation vial containing a magnetic stir bar and dissolved in 4 mL toluene. To this stirred solution was added neat *p*-tolyl azide (0.056 g, 0.421 mmol), resulting in an immediate solution colour change from colourless to gold. The solution was allowed to stir at ambient temperature for 20 min. The volatiles were removed under reduced pressure, and the resulting solid was triturated with hexane (2 x 4 mL) and extracted into HMDSO (6 mL). The solution was concentrated to 2 mL, filtered through Celite, and placed in the freezer at -35 °C for 16 h, resulting in isolation of **3** as a tan solid (0.295 g, 83.4%). ^{1}H NMR (600 MHz, C_6D_6): δ 7.81 (m, CH_{ptolyl}, 2H), 7.19 (m, CH_{ptolyl}, 2H), 3.99 (s, CH_2SiMe$_3$, 2H), 3.64 (sept, C*H*Me$_2$, 6H, $^3J_{H,H}$ = 6.6 Hz), 2.26 (s, C$H_{3,ptolyl}$, 3H), 1.68 (s, NC(CH_3)N, 9H), 1.16 (d, CH*Me*$_2$, 36H, $^3J_{H,H}$ = 6.6 Hz), 0.45 (s, CH$_2$Si*Me*$_3$, 9H). ^{13}C{^{1}H} NMR (151 MHz, C_6D_6): δ 174.0 (N*C*N), 150.8 (C_{ptolyl}), 132.0 (C_{ptolyl}), 129.2 (C_{ptolyl}), 120.8 (C_{ptolyl}), 49.9 (*C*H$_2$SiMe$_3$), 48.6 (*C*HMe$_2$), 25.1 (CH*Me*$_2$), 21.2 (H$_3C_{ptolyl}$), 13.7 (NC(*C*H$_3$)N), 1.55 (CH$_2$Si*Me*$_3$). Anal. Calcd for $C_{35}H_{69}N_9SiTh$ (876.1): C, 47.98; H, 7.94; N, 14.39. Found: C, 47.98; H, 7.88; N, 14.18; Mp: 180 °C (decomp.) FTIR (Nujol): 1489 (s), 1344 (s), 1312 (m), 1243 (w), 1199 (m), 1173 (w), 1123 (w), 1051 (w), 1013 (w), 980 (w), 852 (m), 821 (w), 805 (w), 722 (w). Crystals suitable for single-crystal X-ray diffraction studies were grown from a concentrated diethyl ether solution stored at -35 °C for 48 h.

Generation of Th[(*p*-tolyl)N($SiMe_3$)](BIMA)$_3$ (4): Solid **3** (0.010 g, 0.0114 mmol) was added to a 4 mL dram vial and dissolved in C_6D_6 (0.6 mL). The solution was transferred to a *J*-Young NMR tube sealed with a Teflon screw cap and heated at 70 °C for 48 h, resulting in clean conversion to **4** as judged by ^{1}H NMR spectroscopy. ^{1}H NMR (600 MHz, C_6D_6): δ 7.22 (m, CH_{ptolyl}, 2H), 7.11 (m, CH_{ptolyl}, 2H), 3.60 (sept, C*H*Me$_2$, 6H, $^3J_{H,H}$ = 6.5 Hz), 2.26 (s, C$H_{3,ptolyl}$, 3H), 1.74 (s, NC(CH_3)N, 9H), 1.20 (d, CH*Me*$_2$, 36H, $^3J_{H,H}$ = 6.5 Hz), 0.55 (s, Si*Me*$_3$, 9H). ^{13}C{^{1}H} NMR (151 MHz, C_6D_6): δ 174.4 (N*C*N), 151.0 (C_{ptolyl}), 130.5 (C_{ptolyl}), 123.5 (C_{ptolyl}), 48.4 (*C*HMe$_2$), 25.1 (CH*Me*$_2$), 20.8 (H$_3C_{ptolyl}$), 15.6 (NC(*C*H$_3$)N), 3.40 (Si*Me*$_3$). Crystals suitable for single-crystal X-ray diffraction studies were grown from a concentrated pentane solution stored at -35 °C for 7 d.

Synthesis of Th[*η*^{2}-(C=N)-2,6-Me$_2$-C$_6$H$_3$(CH$_2$SiMe$_3$)](BIMA)$_3$ (5): Solid **1** (0.400 g, 0.538 mmol) and 2,6-dimethylphenyl isocyanide (0.0717 g, 0.547 mmol) were combined in a 100 mL Schlenk flask containing a magnetic stir bar and dissolved in toluene (10 mL). The solution was then heated to 65 °C and stirred for 16 h. The volatiles were removed under reduced pressure, and the resulting solid was triturated with hexane (2 x 4 mL) and extracted into HMDSO (6 mL). The solution was then filtered through Celite, concentrated to ~0.6 mL and placed in the freezer at -35 °C for 16 h, resulting in off-white crystals of **5** (0.383 g, 81.3%). ^{1}H NMR (600 MHz, C_6D_6): δ 7.06 (d, *m*-CH_{Xyl}, 2H, $^3J_{H,H}$ = 7.5 Hz), 6.96 (t, *p*-CH_{Xyl}, 1H, $^3J_{H,H}$ = 7.5 Hz), 3.85 (sept, C*H*Me$_2$, 6H, $^3J_{H,H}$ = 6.6 Hz), 2.85 (s, CH_2SiMe$_3$, 2H), 2.18 (s, C$H_{3,Xyl}$, 6H), 1.78 (s, NC(CH_3)N, 9H), 1.28 (d, CH*Me*$_2$, 36H, $^3J_{H,H}$ = 6.6 Hz), 0.11 (s, CH$_2$Si*Me*$_3$, 9H). ^{13}C{^{1}H} NMR (151 MHz, C_6D_6): δ 289.2 (-*C*=NH$_3$C$_6$Me$_2$), 174.3 (N*C*N), 152.6 (C_{Xyl}), 129.2 (C_{Xyl}), 128.2 (C_{Xyl}), 124.2 (C_{Xyl}), 48.4 (*C*HMe$_2$), 35.0 (*C*H$_2$SiMe$_3$), 25.4 (CH*Me*$_2$), 19.2 (-C=NH$_3$C$_6$*Me*$_2$), 15.5 (NC(*C*H$_3$)N), 1.1 (CH$_2$Si*Me*$_3$). Anal. Calcd for $C_{37}H_{71}N_7SiTh$ (874.2): C, 50.84; H, 8.19; N, 11.22. Found: C, 50.73; H, 8.00; N, 11.44. Mp: 198(4) °C. FTIR (Nujol): 1497 (s, $\nu_{C=N}$), 1377

(s), 1357 (s), 1336 (s), 1316 (s), 1248 (m), 1196 (s), 1174 (s), 1124 (m), 1093 (m), 1051 (m), 1012 (m), 860 (s), 802 (m), 762 (m), 692 (w), 672 (w), 622 (w), 575 (w), 481 (w), 431 (w). Crystals suitable for single-crystal X-ray diffraction studies were grown from a concentrated pentane solution stored at -35 °C for 16 h.

Synthesis of Th[OC(=CH$_2$)SiMe$_3$](BIMA)$_3$ (6): Solid **1** (0.500 g, 0.673 mmol) was added to a 100 mL Schlenk tube containing a magnetic stir bar and dissolved in hexanes (30 mL). CO gas (16.5 mL, 0.673 mmol) was slowly added via syringe directly into the stirred solution over the course of two minutes at ambient temperature, during which the colourless solution became light green. The solution was then sealed off, and allowed to stir at ambient temperature for 24 h. During this time the solution became nearly colourless. The volatiles were removed under reduced pressure, and the resulting residue was extracted into pentane (10 mL), filtered through Celite, and concentrated to <0.5 mL. The super saturated solution was placed in the freezer at -35 °C for 16 h, resulting in colourless crystals of **6** (0.330 g, 63.6%). ^{1}H NMR (600 MHz, C_6D_6): δ 5.07 (d, H_2C=, 1H, $^2J_{H,H}$ = 1.0 Hz), 4.79 (d, H_2C=, 1H, $^2J_{H,H}$ = 1.0 Hz), 3.66 (sept, C*H*Me$_2$, 6H, $^3J_{H,H}$ = 6.3 Hz), 1.71 (s, NC(CH_3)N, 9H), 1.28 (d, CH*Me*$_2$, 36H, $^3J_{H,H}$ = 6.3 Hz), 0.39 (s, Si*Me*$_3$, 9H). ^{13}C{^{1}H} NMR (151 MHz, C_6D_6): δ 173.0 (N*C*N), 104.7 (=*C*H$_2$), 48.5 (*C*HMe$_2$), 25.2 (CH*Me*$_2$), 13.0 (NC(*C*H$_3$)N), -0.6 (CH$_2$Si*Me*$_3$). Anal. Calcd for $C_{29}H_{62}N_6OSiTh$ (771.0): C, 45.18; H, 8.11; N, 10.90. Found: C, 44.96; H, 8.04; N, 10.94. Mp: 130 °C (decomp.). FTIR (Nujol): 1490 (s, $\nu_{C=C}$), 1377 (s), 1360 (s), 1331 (s), 1314 (s), 1254 (w), 1243 (m), 1219 (m), 1200 (s), 1174 (m), 1123 (m), 1051 (w), 1014 (m), 996 (s), 850 (s), 837 (s), 804 (m), 754 (w), 722 (w), 678 (w), 621 (w), 575 (w), 543 (w). X-ray quality crystals were grown from a mixture of **6** and **7**, as described below.

Generation of Th[OC(=CH$_2$)SiMe$_3$](BIMA)$_3$ (6) and Th[OC(N^{i}Pr)C(CH$_2$SiMe$_3$)(C(Me)N(^{i}Pr))O-κ^{2}O,O′](BIMA)$_2$ (7): Solid **1** (0.500 g, 0.673 mmol) was added to a 75 mL Schlenk tube containing a magnetic stir bar and dissolved in toluene (10 mL). CO was vigorously bubbled through this stirred solution for 5 min., during which the colourless solution became light green. The bubbling was then discontinued, and the tube sealed under an atmosphere of CO. The solution was then stirred at ambient temperature for 1 h, at which point the solution had become colourless. The volatiles were removed under reduced pressure, and the resulting residue was triturated with pentane (2 x 5 mL) and extracted into pentane (10 mL). The solution was filtered through Celite and concentrated to <0.5 mL. The super saturated solution was then placed in the freezer at -35 °C for 48 h, resulting in a microcrystalline solid (0.425 g) which contained both **6** and **7**. Despite repeated crystallization attempts, separation of **6** and **7** could not be achieved due to their similar solubility in non-polar solvents. Complex **6** could be identified by ^{1}H NMR spectroscopy. X-ray quality crystals were grown from a concentrated pentane solution stored at -35 °C for 72 h. Individual crystals of **6** and **7** were selected from a mixture of crystals and distinguished based upon their unit cell.

Generation of Th[-N=C(Ph)(CH$_2$SiMe$_3$)](BIMA)$_3$ (8): Solid **1** (0.020 g, 0.027 mmol) was added to a 4 mL dram vial and dissolved in C_6D_6 (0.3 mL). In a separate dram vial, benzonitrile (0.0083 g, 0.081 mmol) was weighed out and dissolved in C_6D_6 (0.3 mL). The two solutions were then combined and added to a *J*-Young NMR tube sealed with a Teflon screw cap. The

solution was then heated at 100 °C for 3 d, resulting in >95% conversion to **8** as judged by ^{1}H NMR spectroscopy. ^{1}H NMR (300 MHz, C_6D_6): δ 7.89 (m, CH_{Ar}, 2H), 7.17-7.27 (m, CH_{Ar}, 3H), 3.67 (sept, C*H*Me$_2$, 6H, $^3J_{H,H}$ = 6.4 Hz), 2.73 (s, CH_2SiMe$_3$, 2H), 1.73 (s, NC(CH_3)N, 9H), 1.23 (d, CHMe_2, 36H, $^3J_{H,H}$ = 6.4 Hz), 0.20 (s, CH$_2$SiMe_3, 9H). It was also observed that ~0.33 eq. of 2,4,6-triphenyl triazine was also generated along with the ketimide product, as determined by its characteristic ^{1}H NMR resonances. ^{1}H NMR (300 MHz, C_6D_6): δ 8.85 (m, CH_{Ar}, 6H), 7.31 (m, CH_{Ar}, 9H).

Generation of Th(NH-2,6-*i*Pr$_2$-C$_6$H$_3$)(BIMA)$_3$ (9): Solid **1** (0.020 g, 0.027 mmol) was added to a 4 mL dram vial and dissolved in C_6D_6 (0.3 mL). In a separate dram vial, 2,6-diisopropylaniline (0.005 g, 0.028 mmol) was weighed out and dissolved in C_6D_6 (0.3 mL). The two solutions were then combined and added to a *J*-Young NMR tube sealed with a Teflon screw cap. The solution was then heated at 100 °C for 5 d, resulting in >90% conversion to **9** as judged by ^{1}H NMR spectroscopy. ^{1}H NMR (300 MHz, C_6D_6): δ 7.25 (d, CH_{Ar}, 2H, $^3J_{H,H}$ = 7.5 Hz), 6.91 (t, CH_{Ar}, 1H, $^3J_{H,H}$ = 7.5 Hz), 4.94 (brs, N*H*, 1H), 3.65 (sept, C*H*Me$_2$, 6H, $^3J_{H,H}$ = 6.5 Hz), 3.54 (sept, C*H*Me$_2$, 2H, $^3J_{H,H}$ = 6.7 Hz), 1.70 (s, NC(CH_3)N, 9H), 1.42 (d, CHMe_2, 12H, $^3J_{H,H}$ = 6.7 Hz), 1.17 (d, CHMe_2, 36H, $^3J_{H,H}$ = 6.5 Hz).

Generation of Th(O-2,6-^{t}Bu$_2$-C$_6$H$_3$)(BIMA)$_3$ (10): Solid **1** (0.021 g, 0.028 mmol) was added to a 4 mL dram vial and dissolved in C_6D_6 (0.3 mL). In a separate dram vial, 2,6-di-*tert*-butylphenol (0.006 g, 0.029 mmol) was weighed out and dissolved in C_6D_6 (0.3 mL). The two solutions were then combined and added to a *J*-Young NMR tube sealed with a Teflon screw cap. The solution was allowed to stand at ambient temperature for 12 h, at which point full conversion to **10** had been achieved as judged by ^{1}H NMR spectroscopy. ^{1}H NMR (600 MHz, C_6D_6): δ 7.40 (d, CH_{Ar}, 2H, $^3J_{H,H}$ = 7.7 Hz), 6.85 (t, CH_{Ar}, 1H, $^3J_{H,H}$ = 7.7 Hz), 3.71 (sept, C*H*Me$_2$, 6H, $^3J_{H,H}$ = 6.5 Hz), 1.74 (s, NC(CH_3)N, 9H), 1.73 (s, C(CH_3)$_3$, 18H), 1.20 (d, CHMe_2, 36H, $^3J_{H,H}$ = 6.5 Hz).

Synthesis of Th(PHMes)(BIMA)$_3$ (11a): Solid ThCl(BIMA)$_3$ (0.243 g, 0.352 mmol) was added to a 20 mL scintillation vial containing a magnetic stir bar and dissolved in diethyl ether (2 mL). To this stirred solution was added KPHMes (0.082 g, 0.431 mmol) in THF (2 mL) dropwise, resulting in a light orange/yellow, cloudy solution. The reaction was allowed to stir at room temperature for 48 h, at which point the reaction appeared bright yellow. The volatiles were removed under reduced pressure, and the resulting solid was triturated with hexanes (2 x 2 mL), extracted into pentane (6 mL), filtered through Celite and concentrated to ~1.5 mL. The solution was then placed in the freezer at -35 °C for 16 h, resulting in yellow crystals of **11a** (0.164 g, 57.8%) ^{1}H NMR (600 MHz, C_6D_6): δ 7.01 (s, CH_{Ar}, 2H), 3.60 (sept, C*H*Me$_2$, 6H, $^3J_{H,H}$ = 6.4 Hz), 3.42 (d, P*H*, $^1J_{P,H}$ = 195 Hz, 1H), 2.74 (s, *o*-CH_3, 6H), 2.42 (s, *p*-CH_3, 3H), 1.63 (s, NC(CH_3)N, 9H), 1.22 (d, CHMe_2, 36H, $^3J_{H,H}$ = 6.4 Hz). ^{13}C{^{1}H} (151 MHz, C_6D_6): δ 174.3 (N*C*N), 147.5 (d, $C_{Ar,ipso}$, $^1J_{P,C}$ = 35.2 Hz), 137.5 (d, $C_{Ar,o}$, $^2J_{P,C}$ = 9.1 Hz), 129.9 ($C_{Ar,p}$), 127.7 (d, $C_{Ar,m}$, $^3J_{P,C}$ = 2.7 Hz), 48.8 (*C*HMe$_2$), 25.7 (d, H$_3C_{Ar,o}$, $^{TS}J_{P,C}$ = 9.6 Hz), 24.7 (d, CHMe_2, $^{TS}J_{P,C}$ = 2.1 Hz), 21.0 (H$_3C_{Ar,p}$), 13.9 (NC(*C*H$_3$)N). ^{31}P NMR (162 MHz, C_6D_6): δ -45.4 (d, $^1J_{P,H}$ = 195 Hz). Anal. Calcd for $C_{33}H_{63}N_6PTh$ (806.9): C, 49.12; H, 7.87; N, 10.42. Found: C, 48.79; H, 7.85; N, 10.36. Mp: 102(2) °C. FTIR (Nujol): 2337 (w, ν_{P-H}), 1337 (m), 1314 (m), 1196 (m),

1174 (w), 1122 (w), 1056 (w), 1013 (w), 847 (w), 806 (w), 722 (m), 620 (w). Crystals suitable for single-crystal X-ray diffraction studies were grown from a concentrated pentane solution stored at -35 °C for 16 h.

Generation of Th(PPh$_2$)(BIMA)$_3$ (11b): Solid **1** (0.021 g, 0.028 mmol) was added to a 4 mL dram vial and dissolved in C_6D_6 (0.3 mL). In a separate dram vial, $PHPh_2$ (0.0052 g, 0.028 mmol) was weighed out and dissolved in C_6D_6 (0.3 mL). The two solutions were then combined and added to a *J*-Young NMR tube sealed with a Teflon screw cap. The solution was then heated at 100 °C for 4 d, resulting in a bright orange solution and >95% conversion to **11b** as judged by ^{1}H NMR spectroscopy. ^{1}H NMR (600 MHz, C_6D_6): δ 7.81 (m, CH_{Ar}, 4H), 7.22 (m, CH_{Ar}, 4H), 6.96 (m, CH_{Ar}, 2H), 3.60 (sept, C*H*Me$_2$, 6H, $^3J_{H,H}$ = 6.4 Hz), 1.63 (s, NC(CH_3)N, 9H), 1.20 (d, CH*Me*$_2$, 36H, $^3J_{H,H}$ = 6.4 Hz). ^{31}P{^{1}H} NMR (162 MHz, C_6D_6): δ 89.5. It was also observed that the dehydrocoupled product, Ph_2P-PPh_2, was generated in small quantities as determined by its characteristic ^{31}P NMR resonance in C_6D_6 (δ -14.9) as well as an unidentified phosphorus-containing species (^{31}P: δ 106). Synthesis of **11a** via protonolysis of **1** with mesitylphosphine followed identical reaction conditions.

Synthesis of Th(C≡C-*p*-tolyl)(BIMA)$_3$ (12): Solid **1** (0.400 g, 0.538 mmol) was added to a 20 mL scintillation vial containing a magnetic stir bar and dissolved in hexanes (10 mL). To this stirred solution was added neat *p*-tolylacetylene (0.065 g, 0.560 mmol) dropwise, resulting in a faint yellow solution which was allowed to stir at ambient temperature for 1 h. The volatiles were removed under reduced pressure, and the resulting solid was extracted into hexanes (10 mL), concentrated to 6 mL, and placed in the freezer at -35 °C for 16 h, resulting in colourless crystals of **12** (0.392 g, 94.5%). ^{1} H NMR (600 MHz, C_6D_6): δ 7.69 (d, CH_{ptolyl}, 2H, $^3J_{H,H}$ = 8.0 Hz), 6.96 (d, CH_{ptolyl}, 2H, $^3J_{H,H}$ = 8.0 Hz), 3.66 (sept, C*H*Me$_2$, 6H, $^3J_{H,H}$ = 6.3 Hz), 2.04 (s, C$H_{3,ptolyl}$, 3H), 1.66 (s, NC(CH_3)N, 9H), 1.42 (d, CH*Me*$_2$, 36H, $^3J_{H,H}$ = 6.3 Hz). ^{13}C{^{1}H} NMR (151 MHz, C_6D_6): δ 189.2 (Th*C*≡C), 172.7 (N*C*N), 134.9 (C_{ptolyl}), 131.0 (C_{ptolyl}), 128.8 (C_{ptolyl}), 124.7 (C_{ptolyl}), 107.8 (ThC≡*C*), 48.3 (*C*HMe$_2$), 24.6 (CH*Me*$_2$), 20.9 (C_6H_4*Me*) 11.8 (NC(*C*H$_3$)N). Anal. Calcd for $C_{33}H_{58}N_6Th$ (770.9): C, 51.42; H, 7.58; N, 10.90. Found: C, 51.72; H, 7.55; N, 10.60. Mp: 131(2) °C. FTIR (Nujol): 2599 (w), 2061 (w, $\nu_{C≡C}$), 1358 (s), 1332 (s), 1313 (s), 1198 (s), 1173 (s), 1137 (w), 1125 (m), 1051 (w), 1016 (m), 819 (s), 803 (s), 711 (w), 618 (w), 569 (w), 546 (w), 534 (w), 464 (m). Crystals suitable for single-crystal X-ray diffraction studies were grown from a concentrated hexanes solution stored at -35 °C for 16 h.

Synthesis of Th[(THF)(BIMA)$_3$][BPh$_4$] (13): Solid **1** (0.300 g, 0.404 mmol) was added to a 20 mL scintillation vial containing a magnetic stir bar and dissolved in THF (4 mL). To this stirred solution was added a THF solution (2 mL) of [Et$_3$NH][BPh$_4$] (0.170 g, 0.403 mmol), and the resulting colourless solution was allowed to stir at ambient temperature for 2 h. The volatiles were removed under reduced pressure, and the resulting solid was re-dissolved in THF (6 mL), concentrated to 2 mL, and placed in the freezer at -35 °C for 16 h, resulting in colourless crystals of **13** (0.368 g, 81.4%). The complex crystallizes with an equivalent of THF in the lattice, which can be removed under vacuum. ^{1}H NMR (600 MHz, $CDCl_3$): δ 7.43 (br s, CH_{Ar}, 8H), 7.06 (m, CH_{Ar}, 8H), 6.91 (m, CH_{Ar}, 4H), 3.94 (m, ^{THF}CH_2, 4H), 3.84 (sept, C*H*Me$_2$, 6H, $^3J_{H,H}$ = 6.4 Hz), 2.02 (s, NC(CH_3)N, 9H), 1.91 (m, ^{THF}CH_2, 4H), 1.16 (d, CH*Me*$_2$, 36H, $^3J_{H,H}$ = 6.4 Hz). ^{11}B{^{1}H}

NMR (192 MHz, $CDCl_3$): -7.16 (s). $^{13}C\{^1H\}$ (151 MHz, $CDCl_3$): δ 176.4 (N*C*N), 164.4 (B-$C_{Ph,}$ $^1J_{B,C}$ = 49.7 Hz), 136.4 (C_{Ph}), 125.5 (C_{Ph}), 121.6 (C_{Ph}), 69.6 ($^{THF}CH_2$), 48.7 (*C*HMe₂), 25.6 ($^{THF}CH_2$), 25.2 ($CHMe_2$), 14.0 (NC(CH_3)N). Anal. Calcd for $C_{52}H_{78}N_6O_1B_1Th$ (1046.1): C, 59.71; H, 7.52; N, 8.03. Found: C, 59.38; H, 7.53; N, 8.21. Mp: 183(5) °C. FTIR (Nujol): 1378 (s), 1345 (m), 1315 (m), 1194 (m), 1174 (w), 1124 (w), 1015 (w), 806 (w), 742 (w), 730 (m), 706 (m), 614 (w), 605 (w), 424 (w). Crystals suitable for single-crystal X-ray diffraction studies were grown from a concentrated THF solution stored at -35 °C for 16 h.

Synthesis of $(BIMA)_3Th(\mu\text{-}H)_2[B(C_8H_{14})]$ (14): Solid **1** (0.251 g, 0.338 mmol) was added to a 20 mL scintillation vial containing a magnetic stir bar and dissolved in 2 mL toluene. To this stirred solution was added a toluene solution (3 mL) of 9-BBN dimer (0.082 g, 0.336 mmol) dropwise over 1 minute. The resulting colourless solution was allowed to stir at ambient temperature for 48 h. The volatiles were removed under reduced pressure, and the resulting solid was triturated with hexane (2 x 4 mL) and extracted into HMDSO (8 mL), leaving behind a small amount of insoluble, colourless solid. The HMDSO extract was then filtered through Celite, concentrated to 5 mL, and placed in the freezer at -35 °C for 16 h, resulting in colourless crystals of **14** (0.173 g, 65.8%). ^1H NMR (300 MHz, C_6D_6): δ 4.67-5.30 (br, μ-*H*, 2H), 3.68 (sept, C*H*Me₂, 6H, $^3J_{H,H}$ = 6.5 Hz), 2.25-2.41 (m, BBN, 10H), 2.06 (m, BBN, 2H), 1.78 (brs, BBN, 2H), 1.66 (s, NC(CH_3)N, 9H), 1.23 (d, CHMe_2, 36H, $^3J_{H,H}$ = 6.5 Hz). $^{11}B\{^1H\}$ NMR (192 MHz, C_6D_6): δ 4.90 (s). $^{13}C\{^1H\}$ (126 MHz, C_6D_6): δ 174.6 (N*C*N), 48.7 (*C*HMe₂), 34.5 (BBN), 25.8 (BBN), 24.7 (CHMe_2), 14.6 (NC(CH_3)N). Anal. Calcd for $C_{32}H_{67}BN_6Th$ (778.8): C, 49.35; H, 8.67; N, 10.79. Found: C, 49.30; H, 8.59; N, 10.68; Mp: 207(2) °C. FTIR (Nujol): 2021 cm^{-1} (br, B-H), 1377 (s), 1334 (s), 1313 (s), 1283 (s), 1197 (s), 1171 (s), 1119 (m), 1051 (m), 1011 (m), 919 (w), 805 (m), 775 (w), 723 (m), 618 (w), 575 (w), 543 (w). Crystals suitable for single-crystal X-ray diffraction studies were grown from a concentrated pentane solution stored at -35 °C for 16 h.

Synthesis of $Th(BIMA)_2(BIMA^*)$ (15): Solid **1** (0.500 g, 0.673 mmol) was added to a 4 mL dram vial and dissolved in 1.5 mL C_6D_6. The solution was transferred to a *J*-Young NMR tube and sealed with a Teflon screw cap. The *J*-Young tube was then placed in the photolysis reactor and irradiated for 10 days, at which point the solution had changed from colourless to green, and **1** was completely consumed as observed by ^1H NMR spectroscopy. The *J*-Young tube was then brought into the glovebox and emptied into a 20 mL scintillation vial, and the tube was rinsed with toluene (2 mL). This rinse was combined with the C_6D_6 solution, and the volatiles were then removed under reduced pressure. The resulting solid was then extracted into toluene (4 mL), concentrated to 1 mL and placed in the freezer at -35 °C for 16 h, resulting in colourless crystals of **15** (0.232 g, 52.7%). ^1H NMR (600 MHz, C_6D_6): δ 4.24 (sept, C*H*Me₂*, 2H, $^3J_{H,H}$ = 6.2 Hz), 3.57 (sept, C*H*Me₂, 4H, $^3J_{H,H}$ = 6.2 Hz), 3.44 (s, NC(CH_2)N*, 2H), 1.54 (d, CHMe_2*, 12H, $^3J_{H,H}$ = 6.2 Hz), 1.53 (s, NC(CH_3)N, 6H), 1.15 (d, CHMe_2, 24H, $^3J_{H,H}$ = 6.2 Hz). $^{13}C\{^1H\}$ NMR (151 MHz, C_6D_6): δ 172.8 (N*C*N), 154.2 (N*C*N*), 53.3 (NC(CH_2)N*), 49.1 (Me₂*C*H*), 48.3 (Me₂*C*H), 26.0 (Me_2CH*), 25.7 (Me_2CH), 12.2 (NC(CH_3)N). Anal. Calcd for $C_{24}H_{50}N_6Th$ (654.7): C, 44.03; H, 7.70; N, 12.84. Found: C, 43.77; H, 7.67; N, 12.46. Mp: 170(decomp.) °C. FTIR (Nujol): 1496 (s), 1414 (s), 1308 (s), 1197 (s), 1169 (m), 1123 (m), 1049 (m), 1010 (m), 886 (w), 796 (s), 722 (w), 695 (m), 620 (w), 577 (w), 546 (w), 456 (w), 433 (w). Crystals

suitable for single-crystal X-ray diffraction studies were grown from a concentrated toluene solution stored at -35 °C for 16 h. Alternatively, solid **1** (0.009 g, 0.012 mmol) was added to a 4 mL dram vial and dissolved in 0.4 mL C_6D_{12}. The solution was transferred to a quartz EPR tube custom-fitted with a *J*-Young neck and sealed with a Teflon screw cap. The tube was then placed in front of a xenon arc lamp and irradiated for 2 h, at which point the solution had changed from colourless to green, and **1** was completed consumed as observed by ^{1}H NMR spectroscopy.

B. NMR Spectroscopic Data

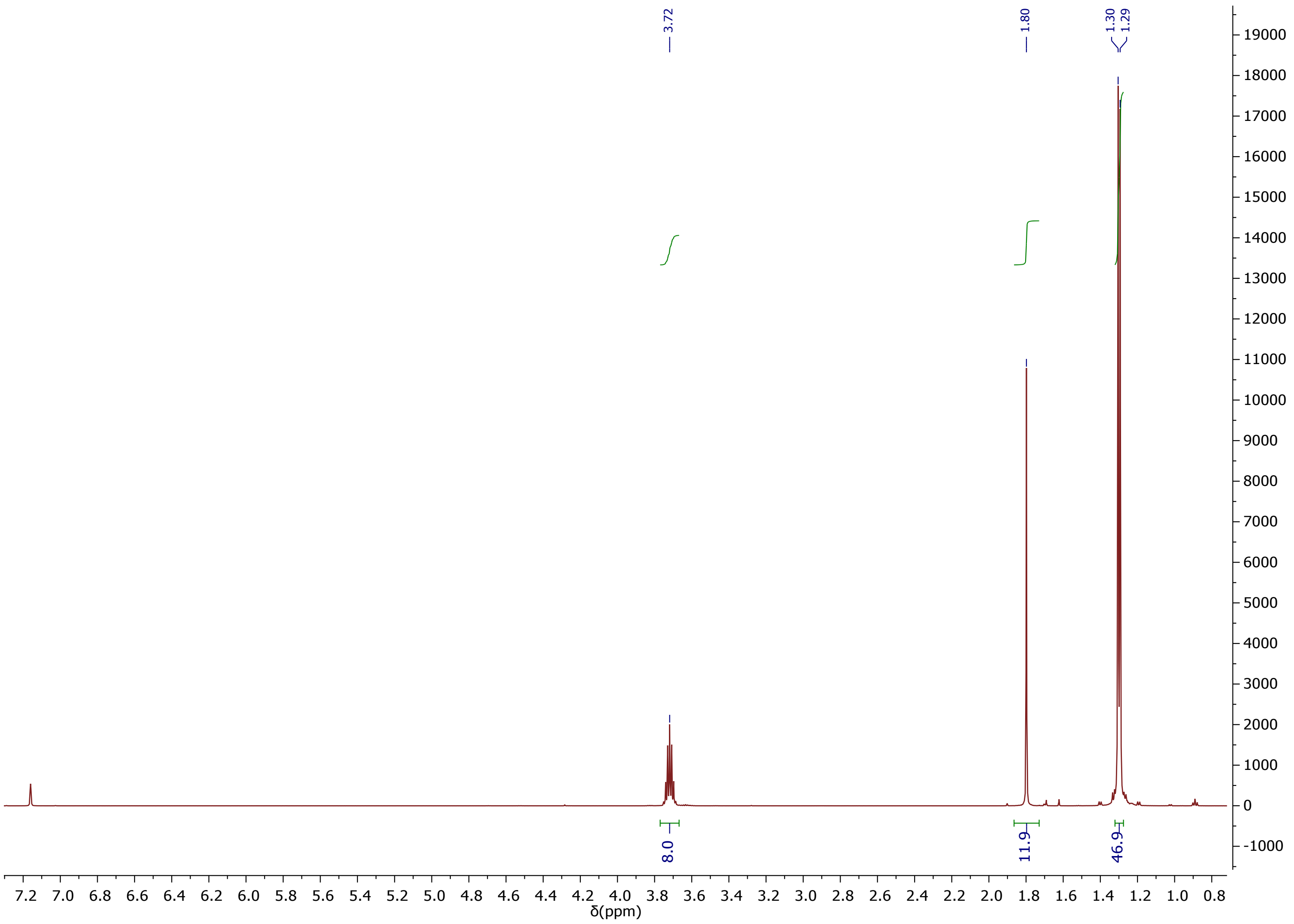

Figure S1. ^{1}H NMR spectrum of $Th(BIMA)_4$ (**2**) at 298 K in C_6D_6

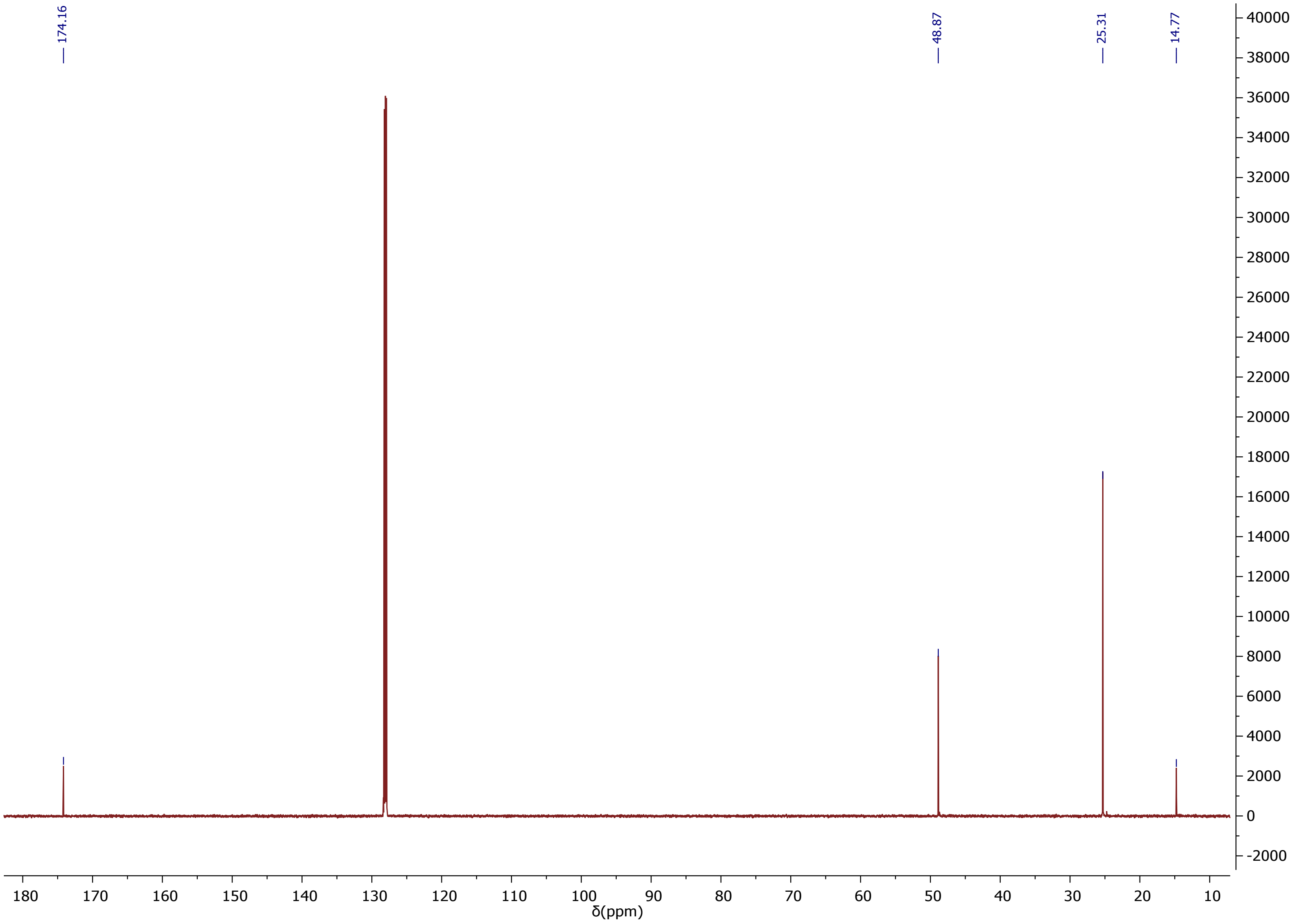

Figure S2. $^{13}C\{^{1}H\}$ NMR spectrum of $Th(BIMA)_4$ (**2**) at 298 K in C_6D_6

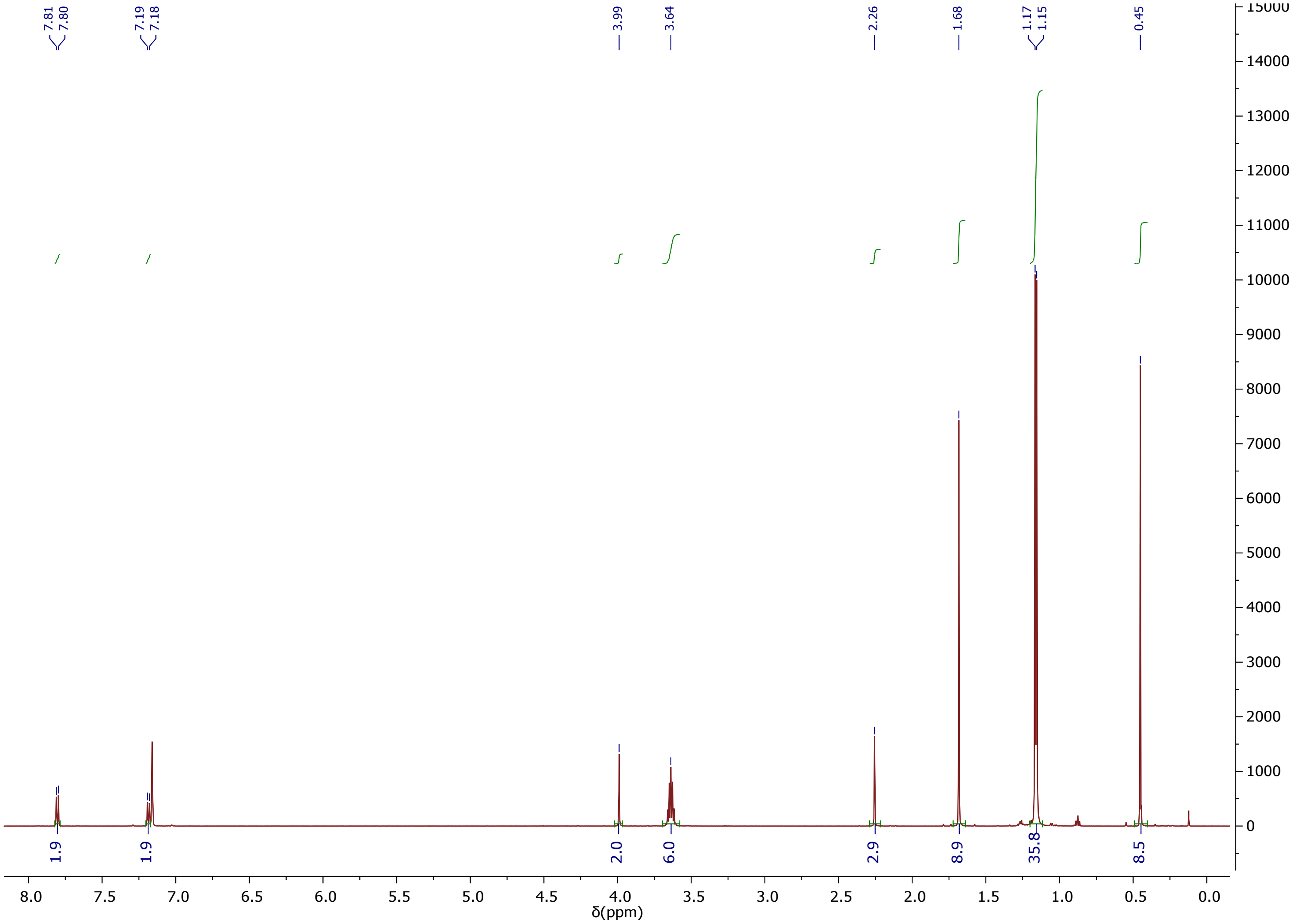

Figure S3. ^{1}H NMR spectrum of Th[(*p*-tolyl)NNN(CH_2SiMe_3)-$\kappa^2N^{1,2}$](BIMA)$_3$ (**3**) at 298 K in C_6D_6

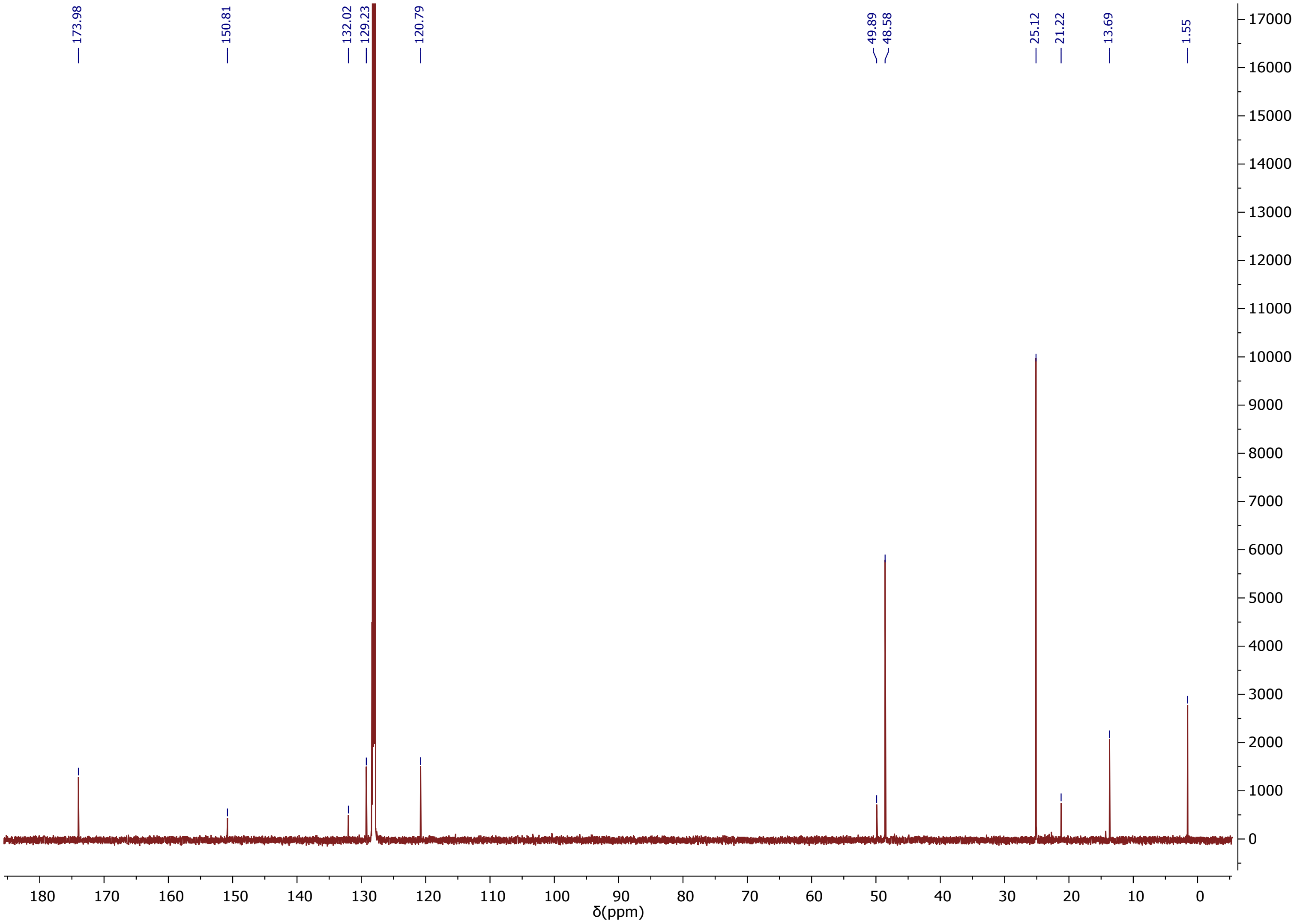

Figure S4. $^{13}C\{^1H\}$ NMR spectrum of Th[(*p*-tolyl)NNN(CH_2SiMe_3)-$\kappa^2N^{1,2}$](BIMA)$_3$ (**3**) at 298 K in C_6D_6

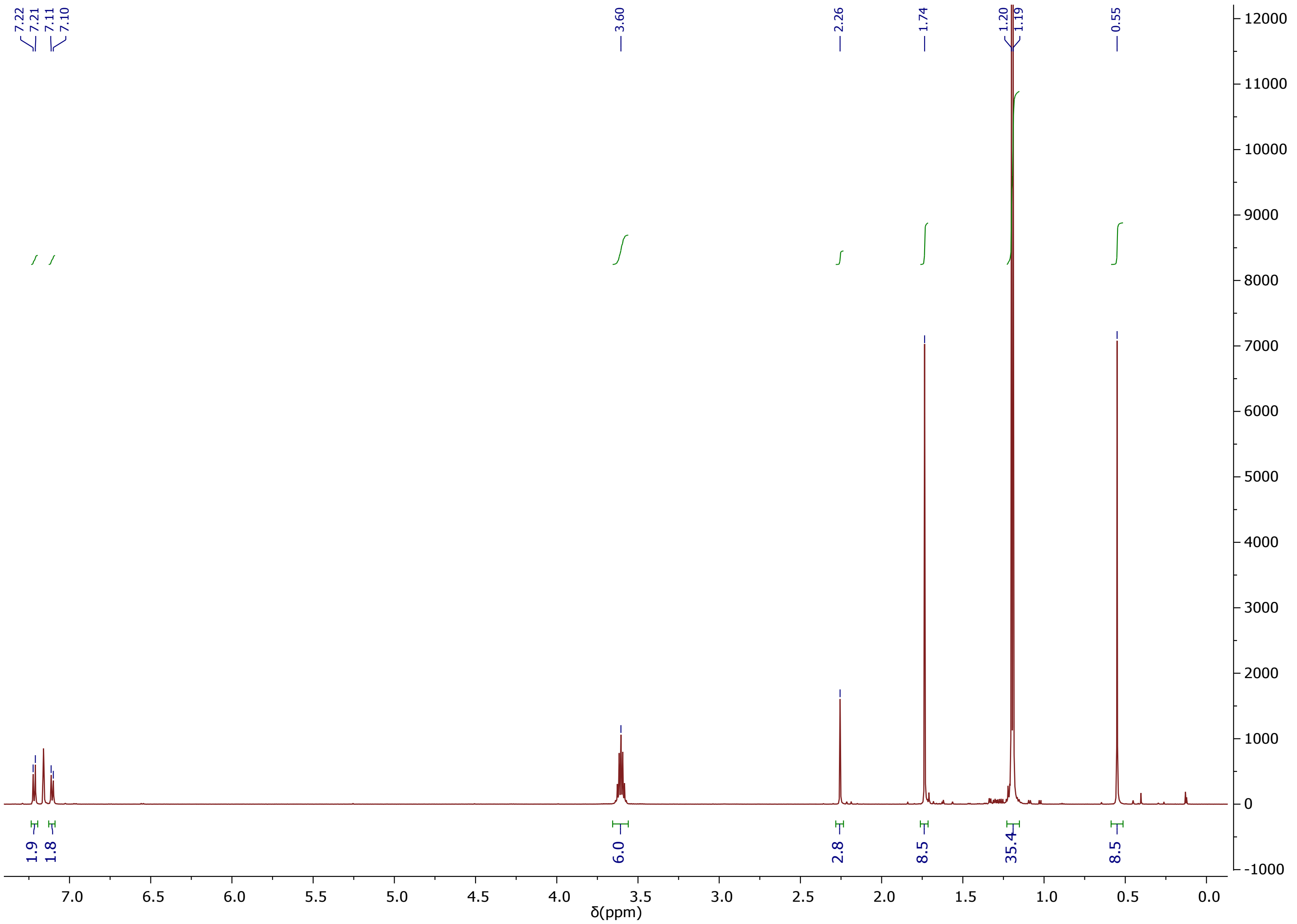

Figure S5. ^{1}H NMR spectrum of Th[(*p*-tolyl)N(SiMe$_3$)](BIMA)$_3$ (**4**) at 298 K in C_6D_6

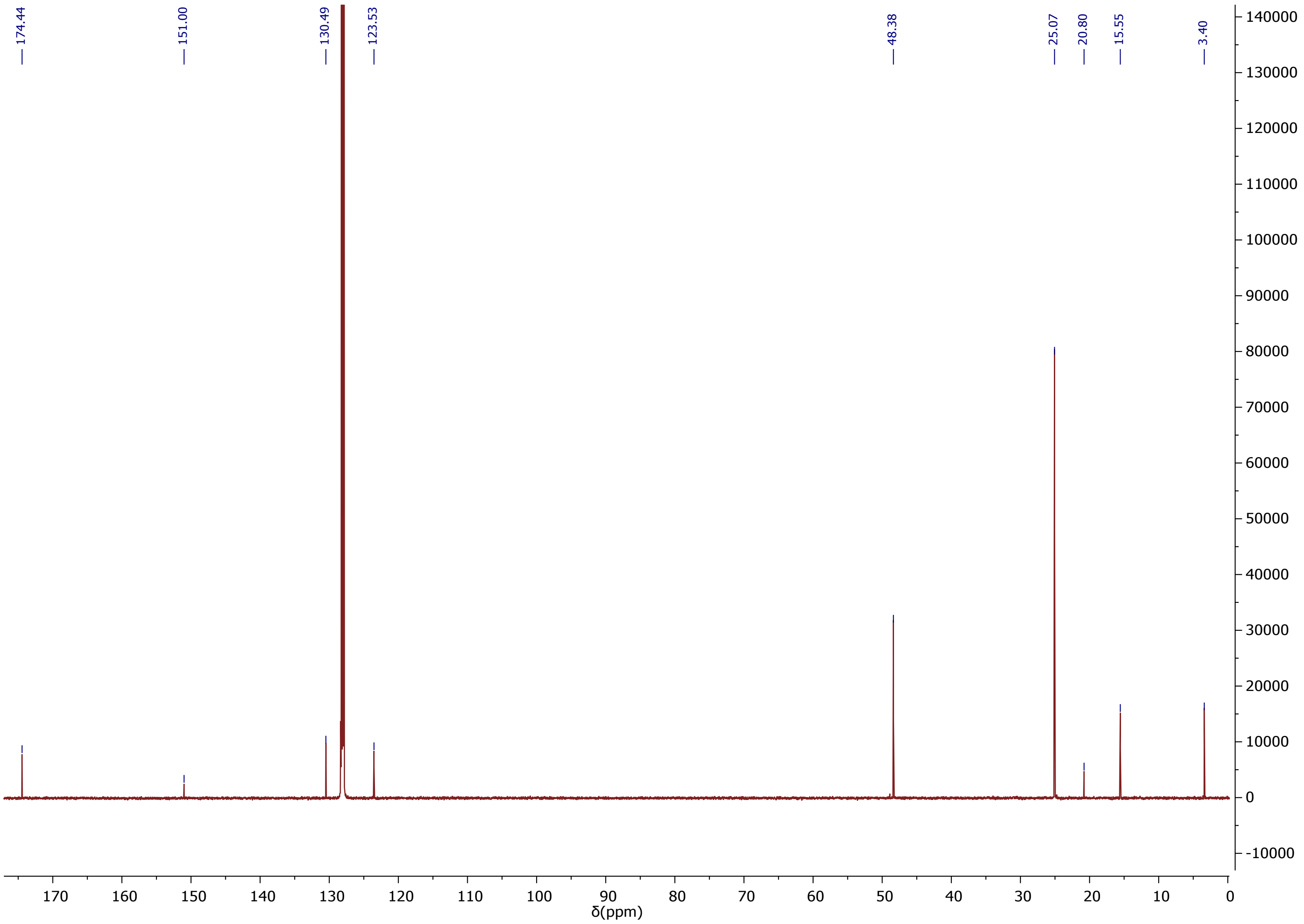

Figure S6. $^{13}C\{^{1}H\}$ NMR spectrum of Th[(*p*-tolyl)N(SiMe$_3$)](BIMA)$_3$ (**4**) at 298 K in C_6D_6

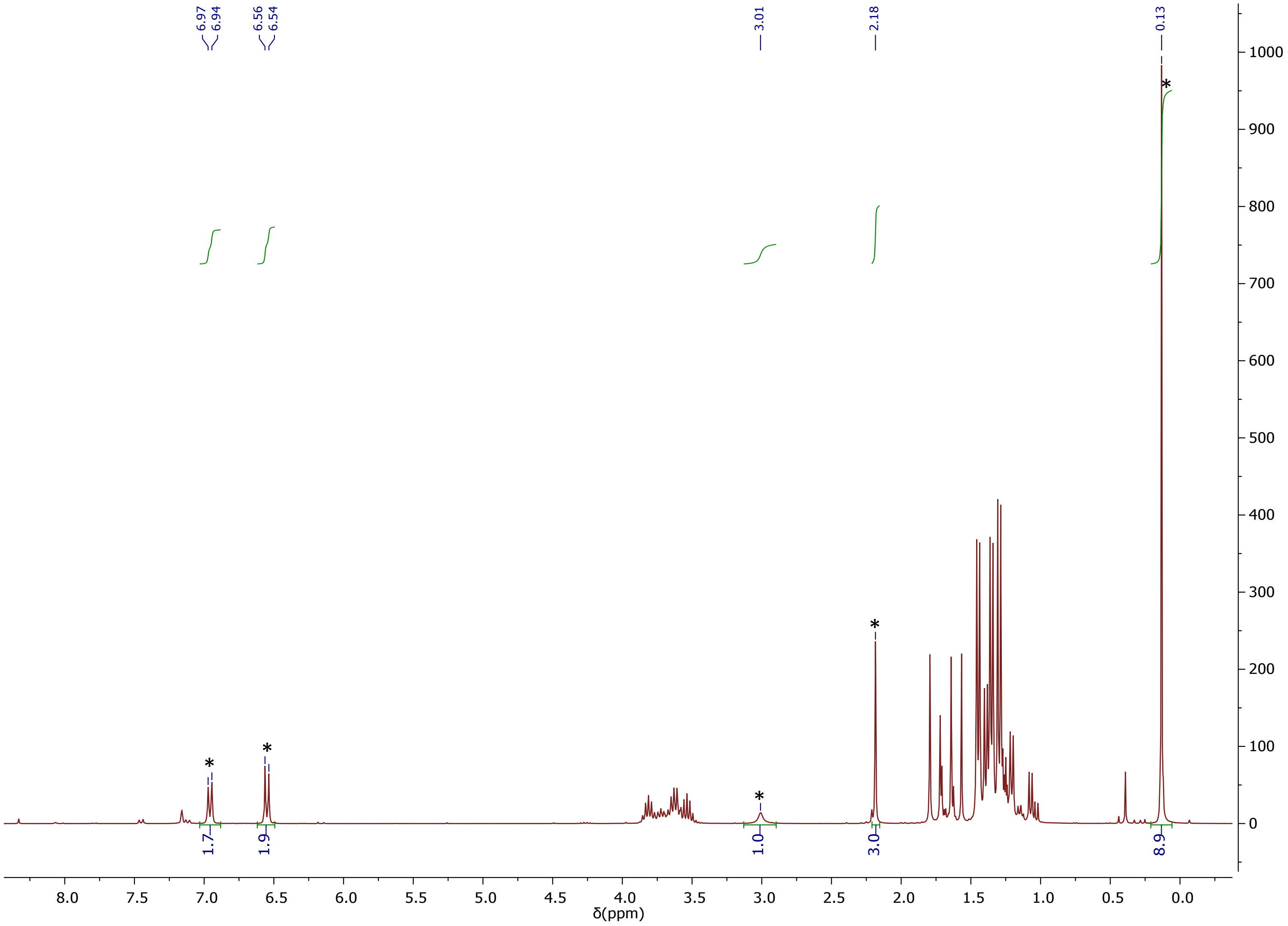

Figure S7. ^{1}H NMR spectrum of **3** (0.100 g, 0.114 mmol) in 0.6 mL C_6D_6 after heating at 70 °C for 4 d. *Resonances attributable to (*p*-tolyl)NH($SiMe_3$)

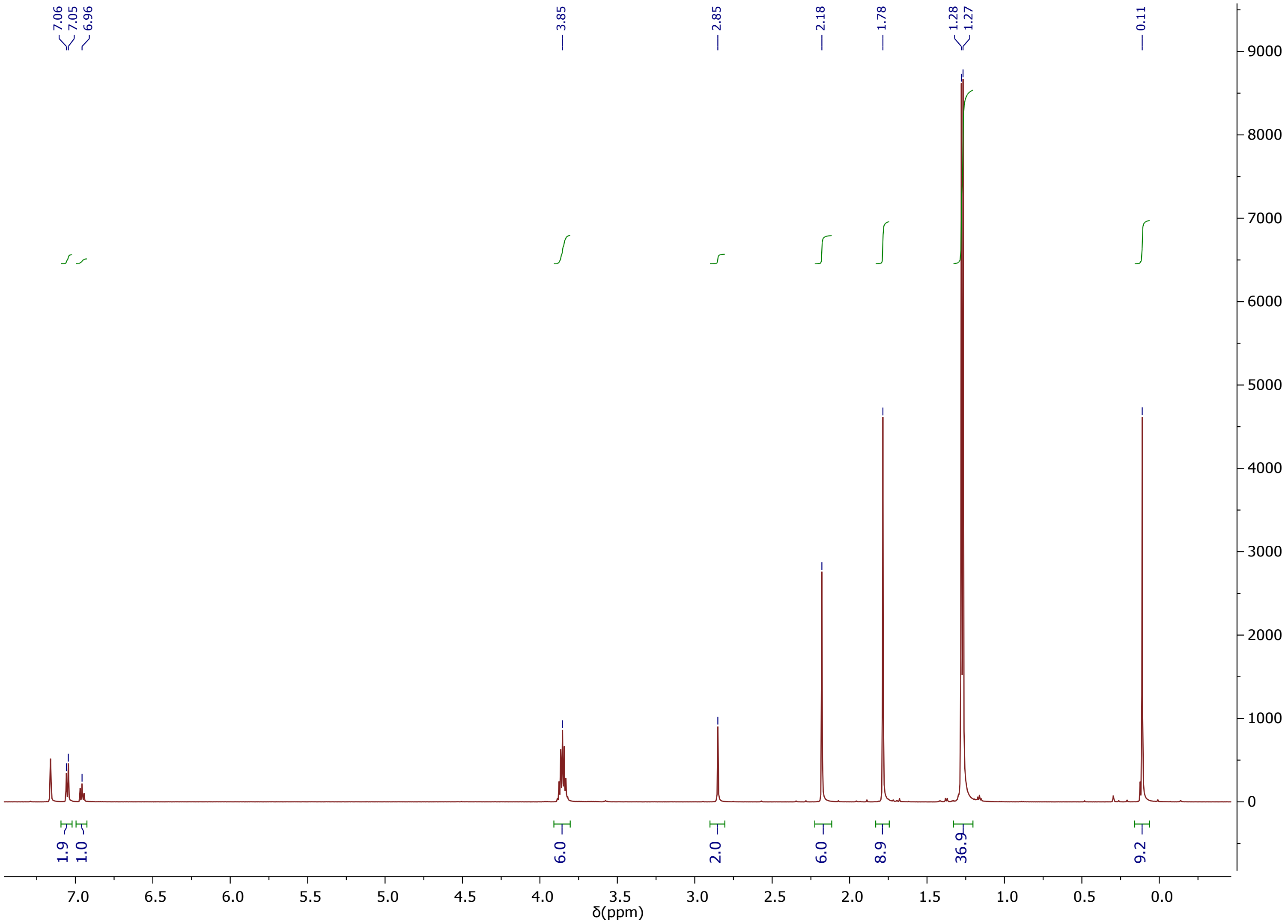

Figure S8. ^{1}H NMR spectrum of Th[η^2-(C=N)-2,6-Me_2-C_6H_3(CH_2SiMe_3)](BIMA)$_3$ (**5**) at 298 K in C_6D_6

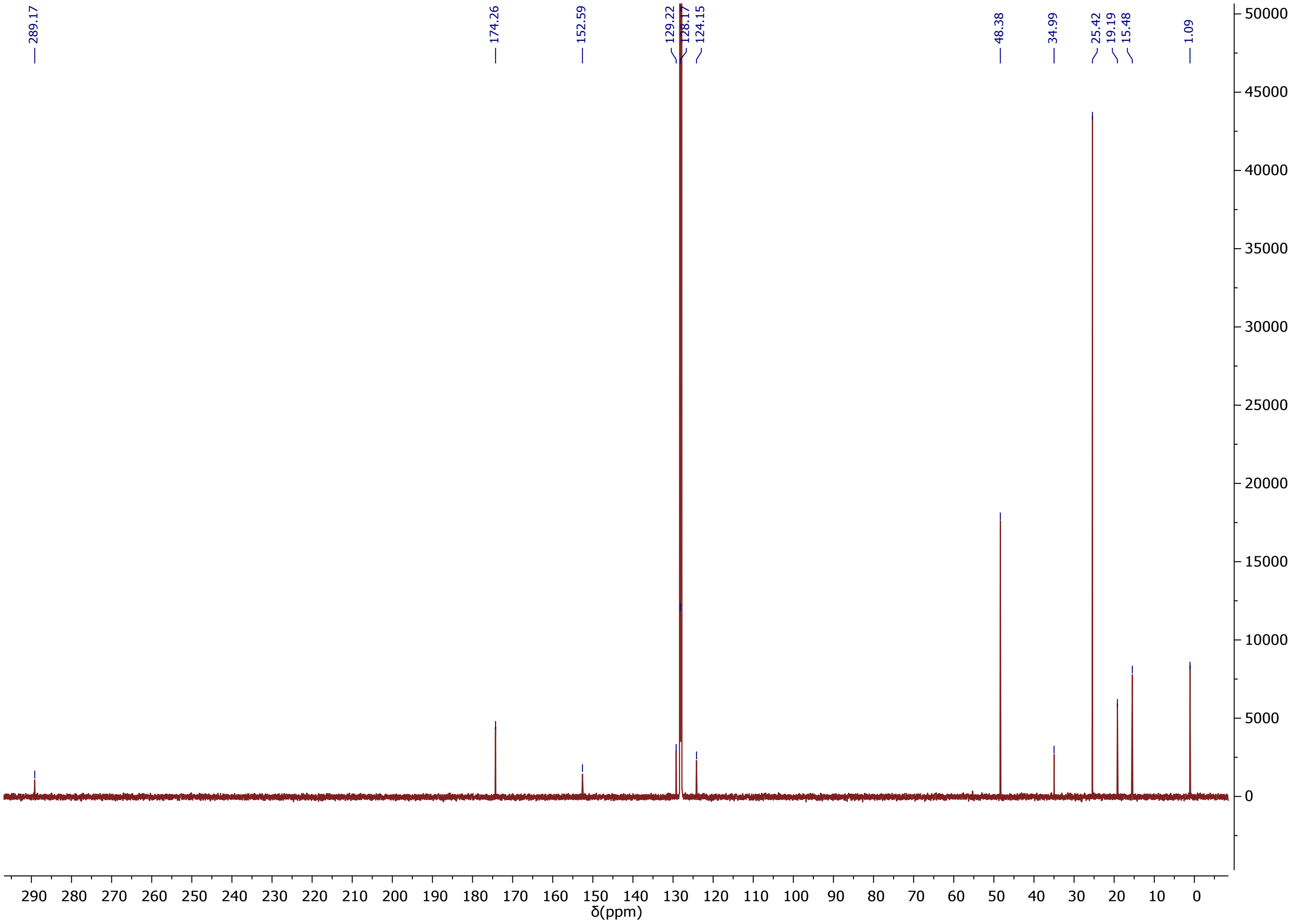

Figure S9. $^{13}C\{^1H\}$ NMR spectrum of Th[η^2-(C=N)-2,6-Me_2-C_6H_3(CH_2SiMe_3)](BIMA)$_3$ (**5**) at 298 K in C_6D_6

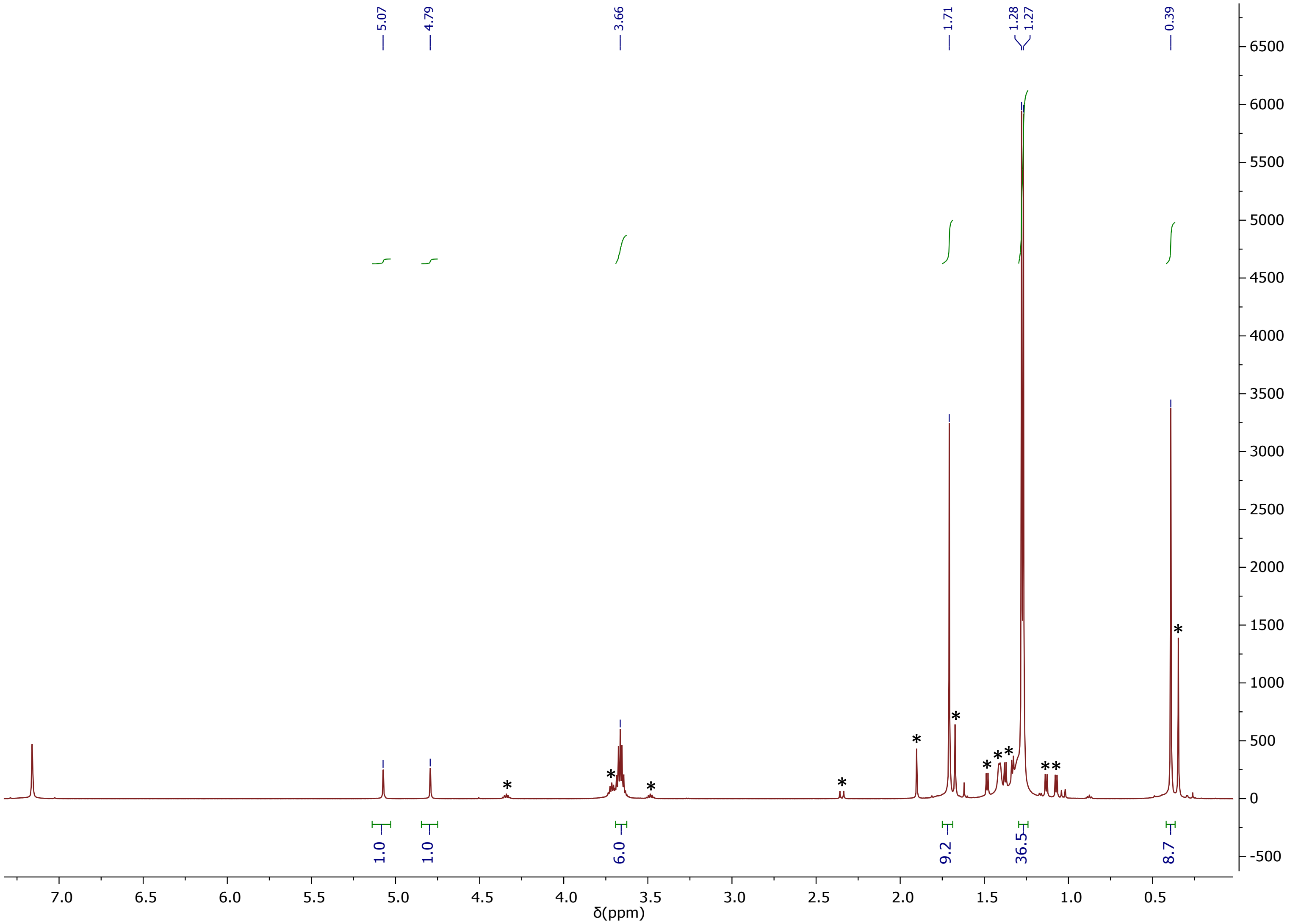

Figure S10. ^{1}H NMR spectrum of bulk mixture of **6** and **7** at 298 K in C_6D_6. Integrated resonances are attributable to **6**. *Resonances attributable to **7**

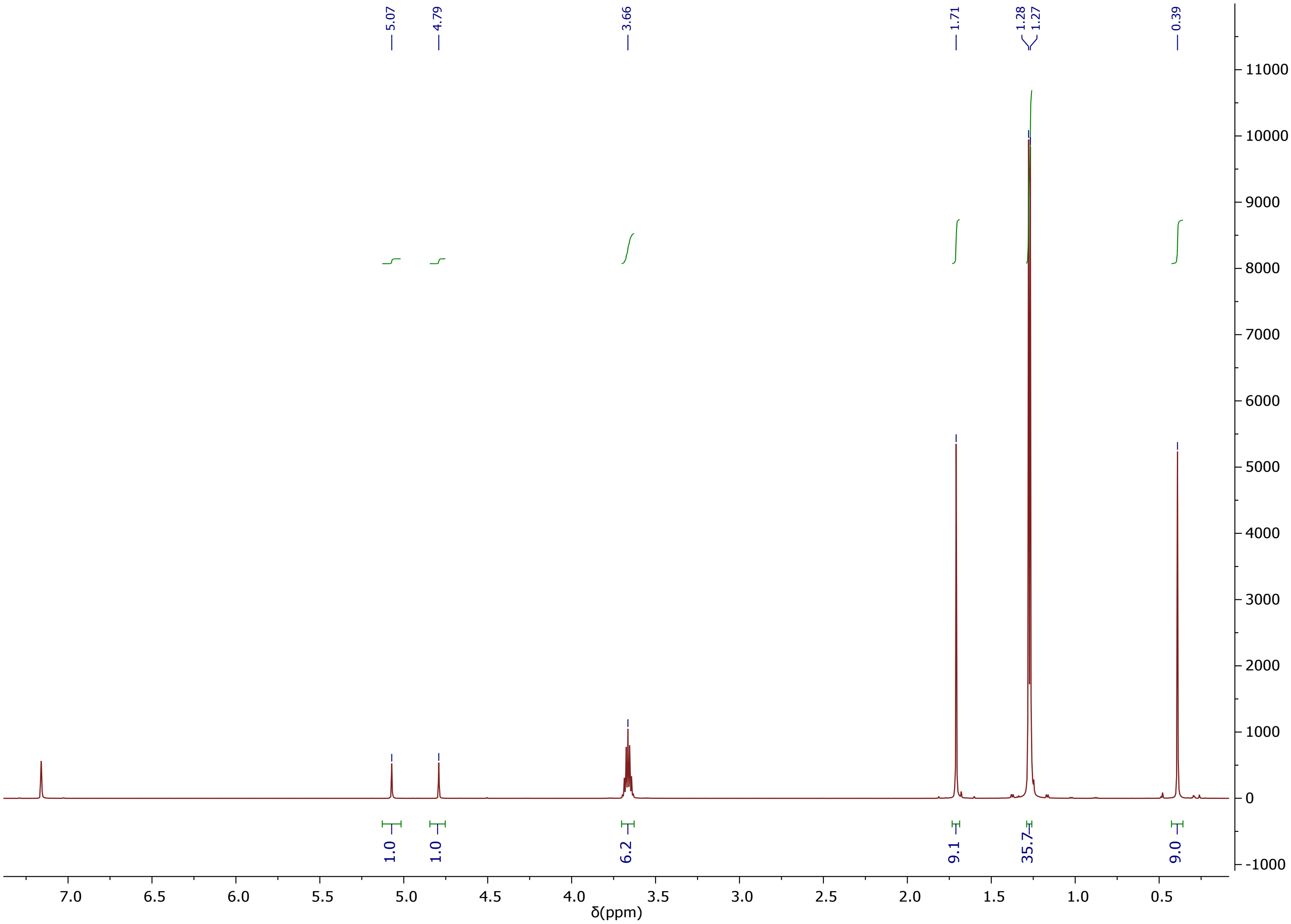

Figure S11. ^{1}H NMR spectrum of Th[OC(=CH_2)$SiMe_3$]$(BIMA)_3$ (**6**) at 298 K in C_6D_6

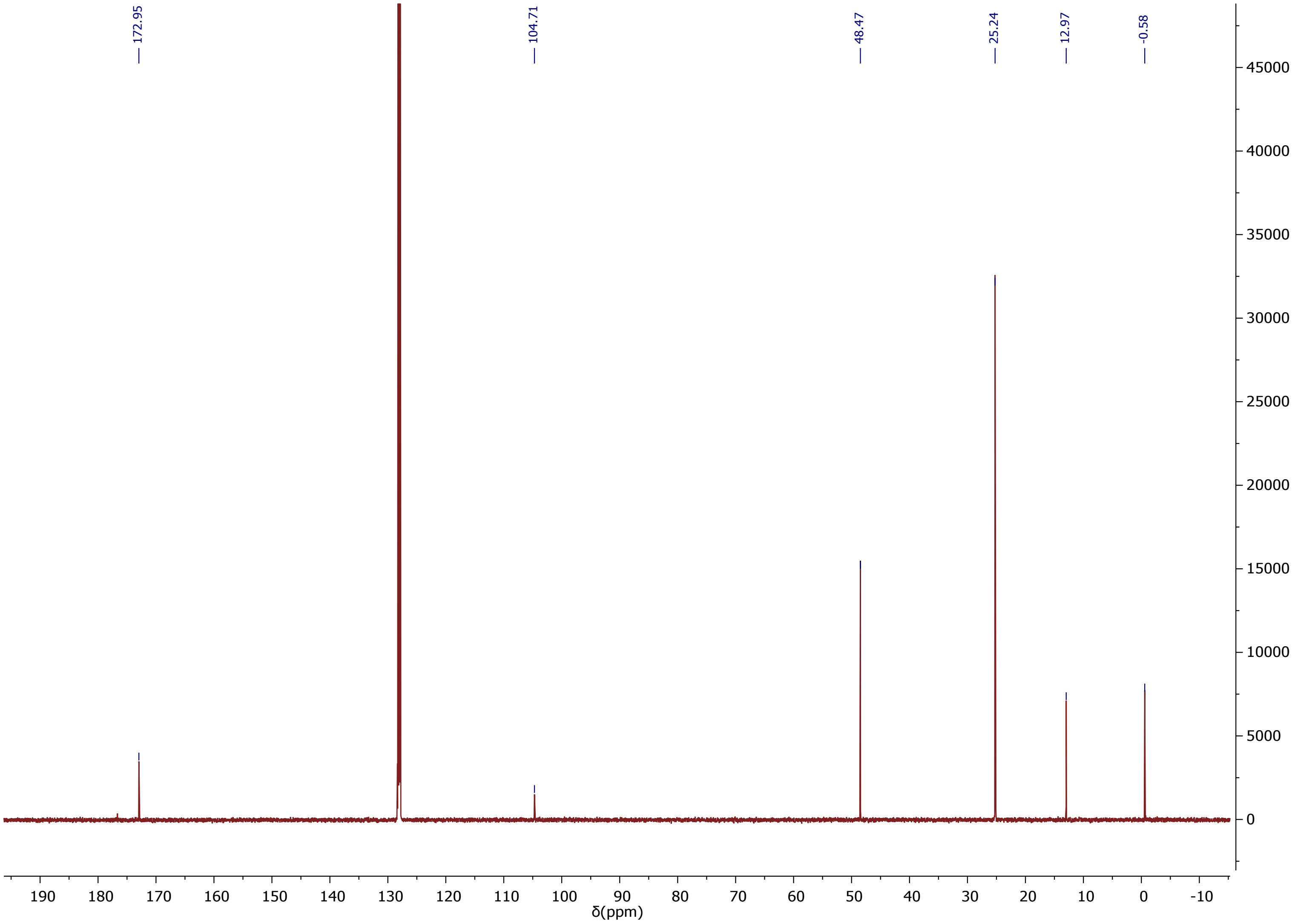

Figure S12. $^{13}C\{^{1}H\}$ NMR spectrum of $Th[OC(=CH_2)SiMe_3](BIMA)_3$ (**6**) at 298 K in C_6D_6

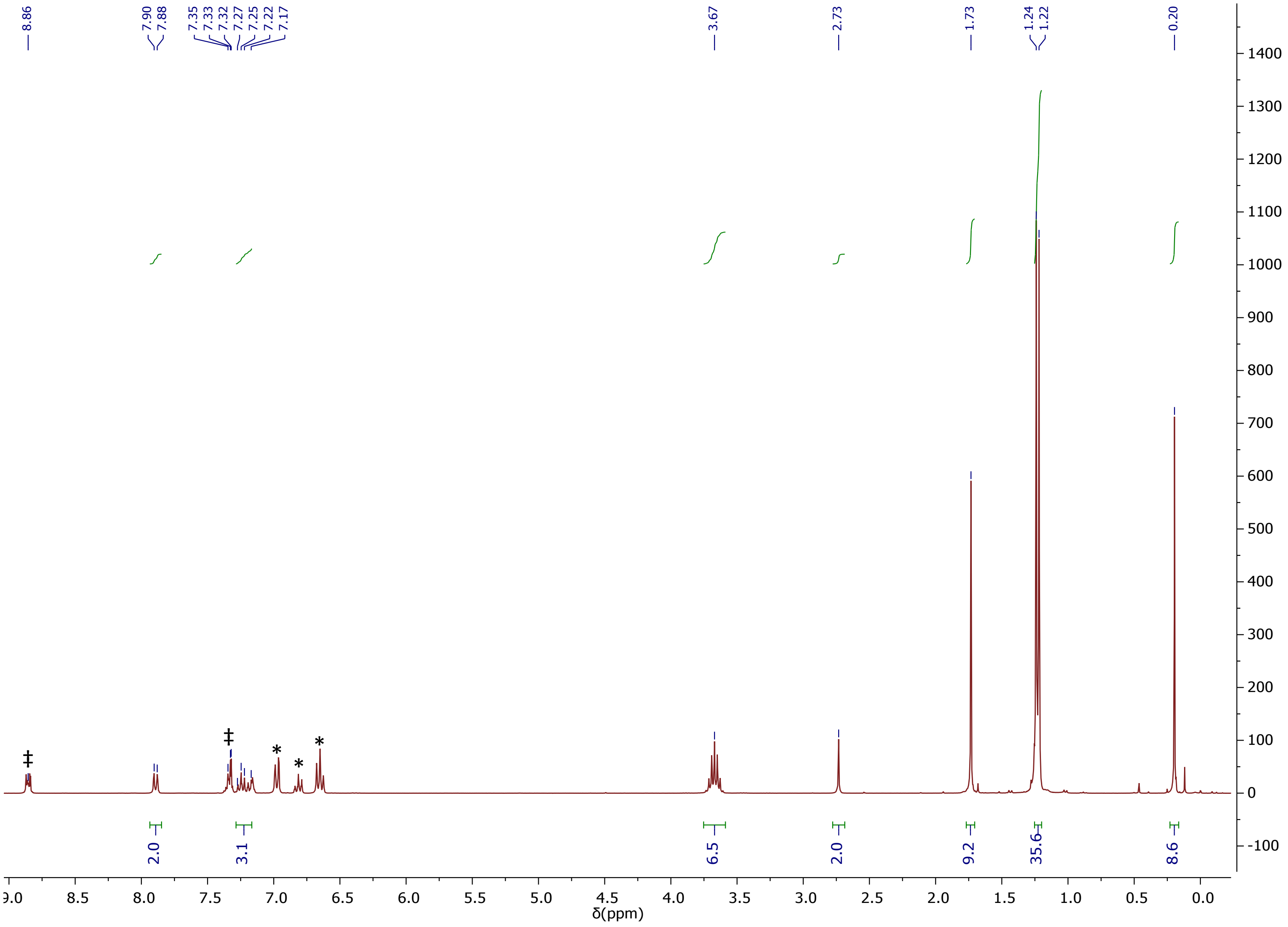

Figure S13. ^{1}H NMR spectrum of Th[-N=C(Ph)(CH_2SiMe_3)]$(BIMA)_3$ (**8**) generated *in situ*, at 298 K in C_6D_6. *PhCN. ‡2,4,6-triphenyl triazine

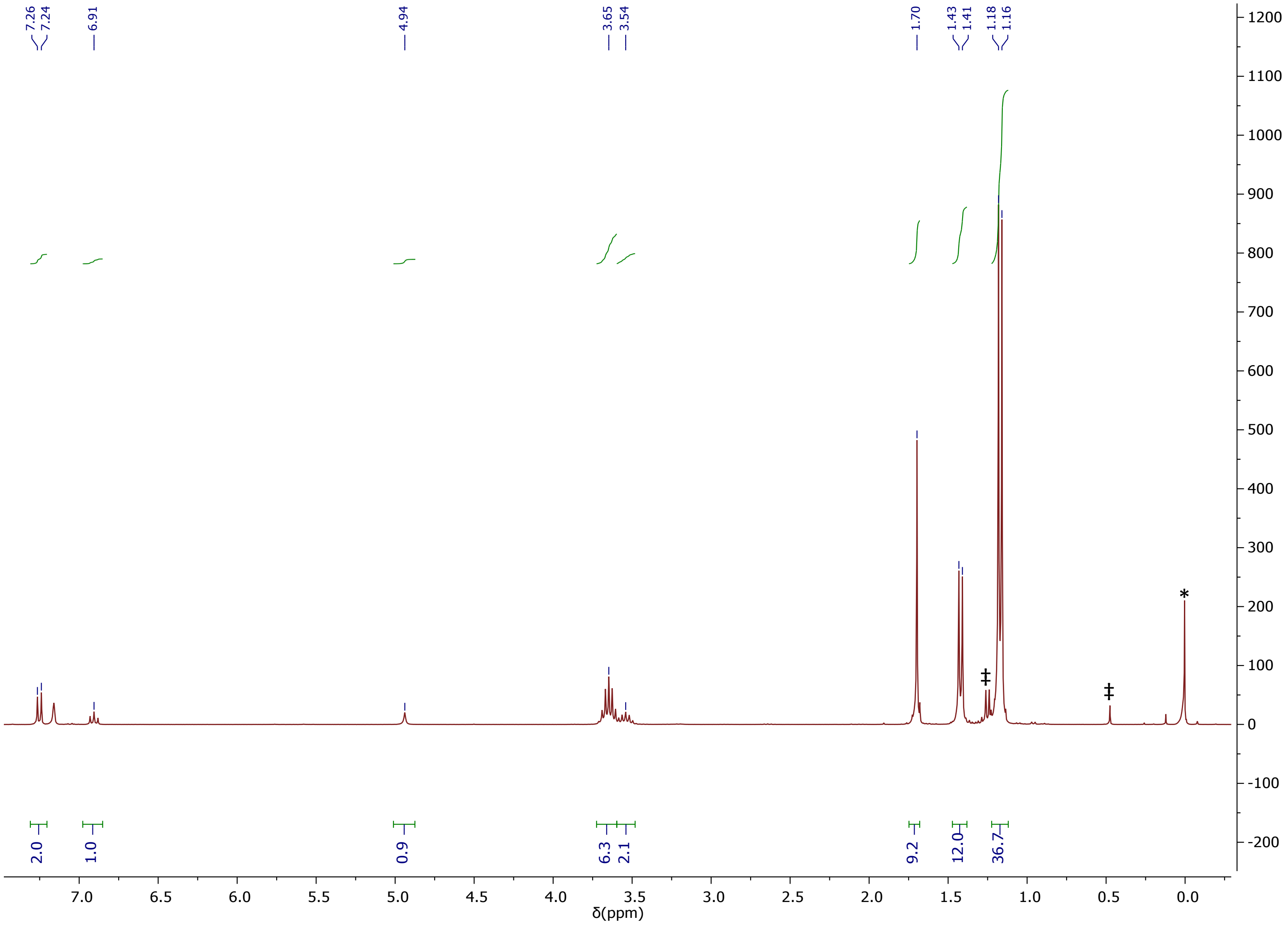

Figure S14. ^{1}H NMR spectrum of Th(NH-2,6-*i*Pr_2-C_6H_3)(BIMA)$_3$ (**9**) generated *in situ*, at 298 K in C_6D_6. *$SiMe_4$. ‡**1**

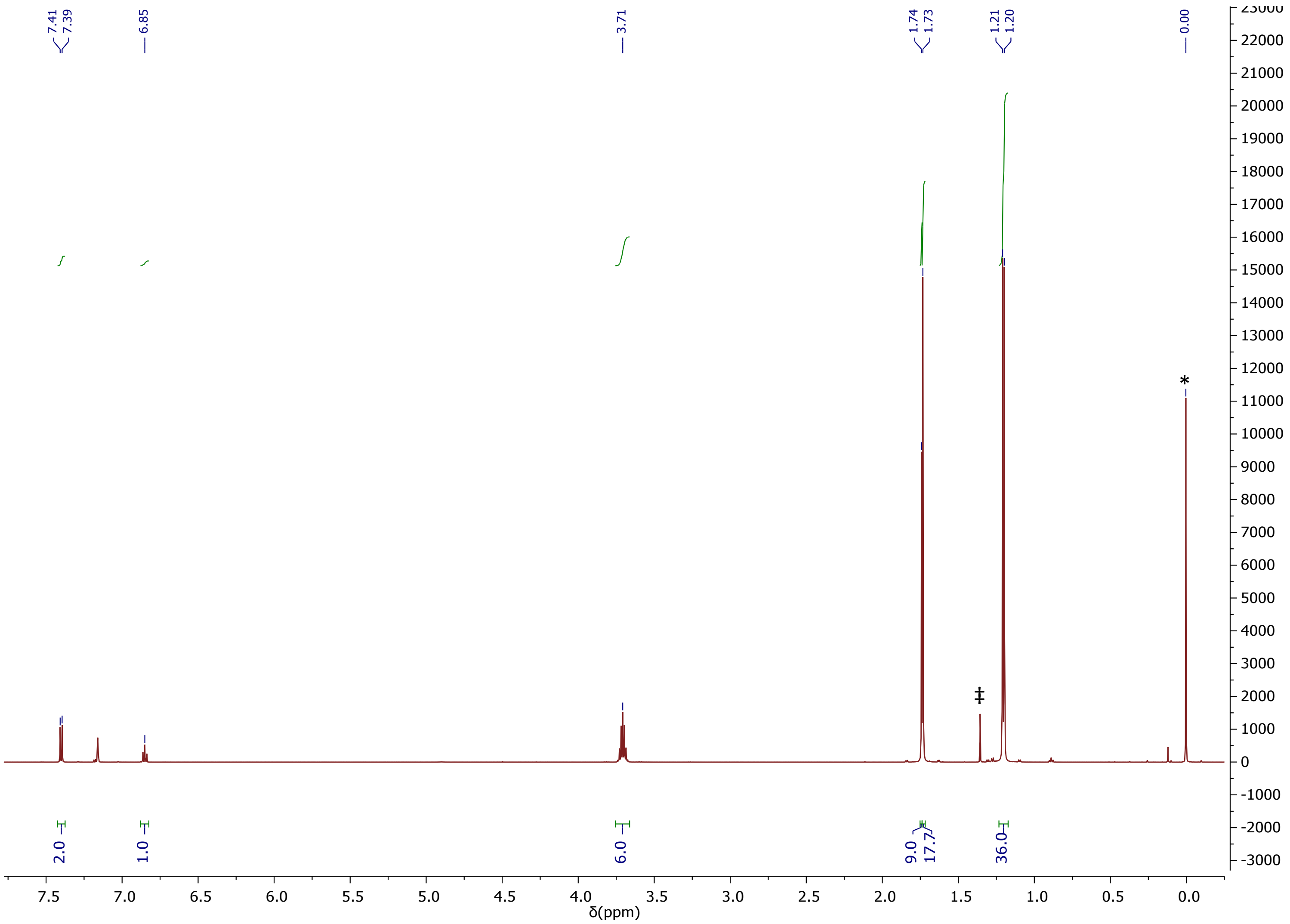

Figure S15. ^{1}H NMR spectrum of Th(O-2,6-^{t}Bu$_2$-C_6H_3)(BIMA)$_3$ (**10**) generated *in situ*, at 298 K in C_6D_6. *$SiMe_4$. ‡2,6-di-*tert*-butylphenol

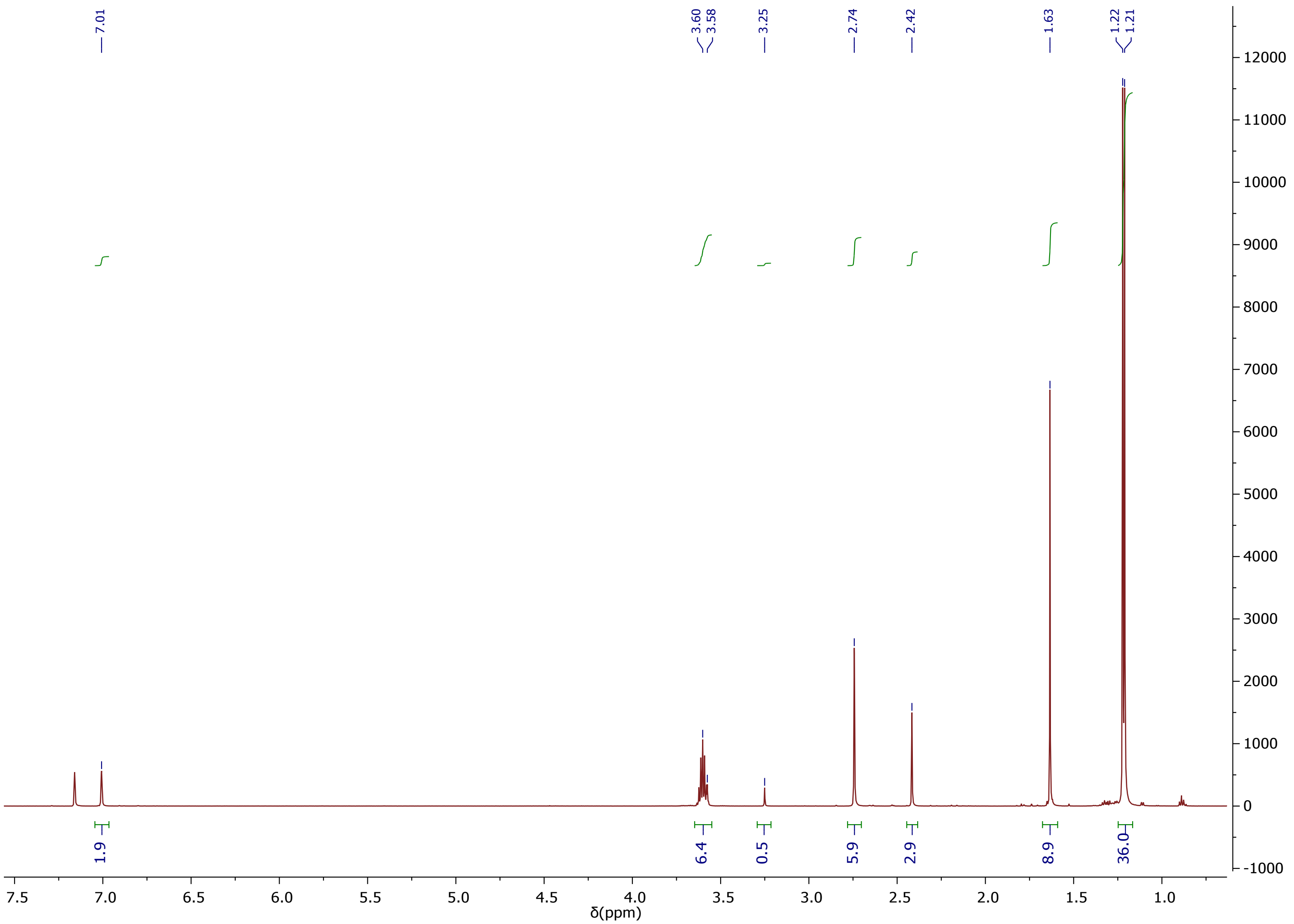

Figure S16. ^{1}H NMR spectrum of Th(PHMes)(BIMA)$_3$ (**11a**) at 298 K in C_6D_6

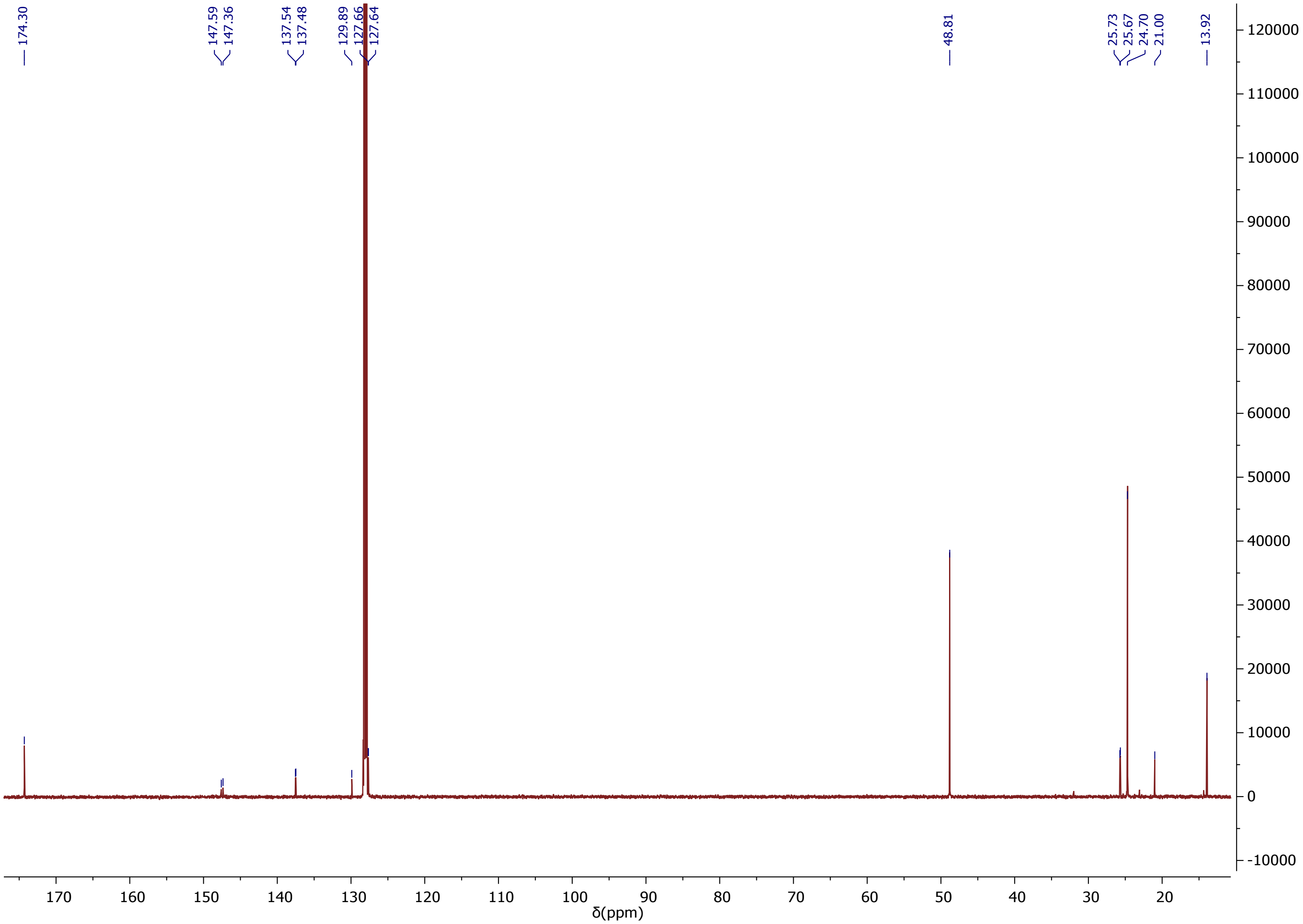

Figure S17. $^{13}C\{^{1}H\}$ NMR spectrum of Th(PHMes)(BIMA)$_3$ (**11a**) at 298 K in C_6D_6

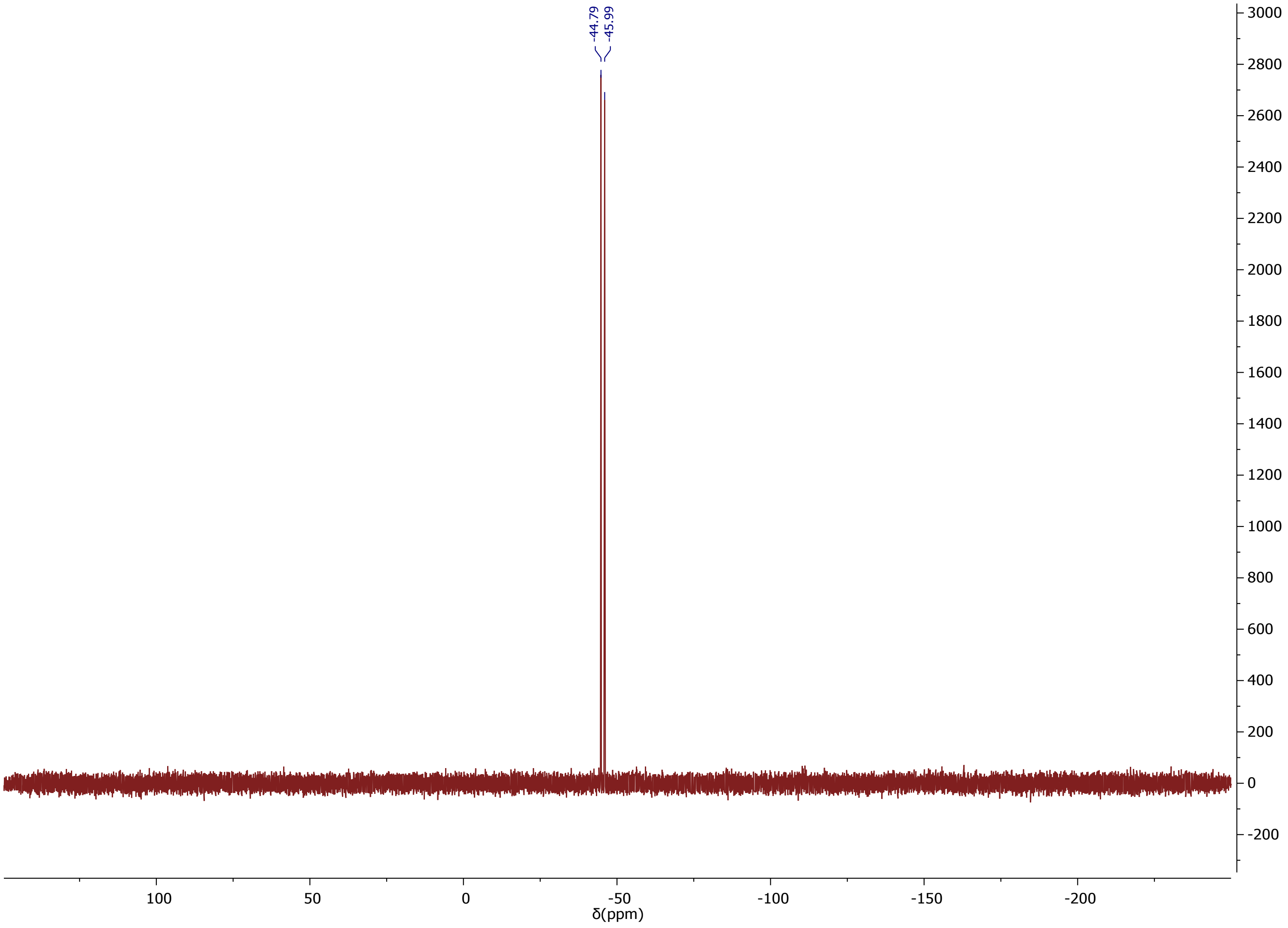

Figure S18. ^{31}P NMR spectrum of Th(PHMes)(BIMA)$_3$ (**11a**) at 298 K in C_6D_6

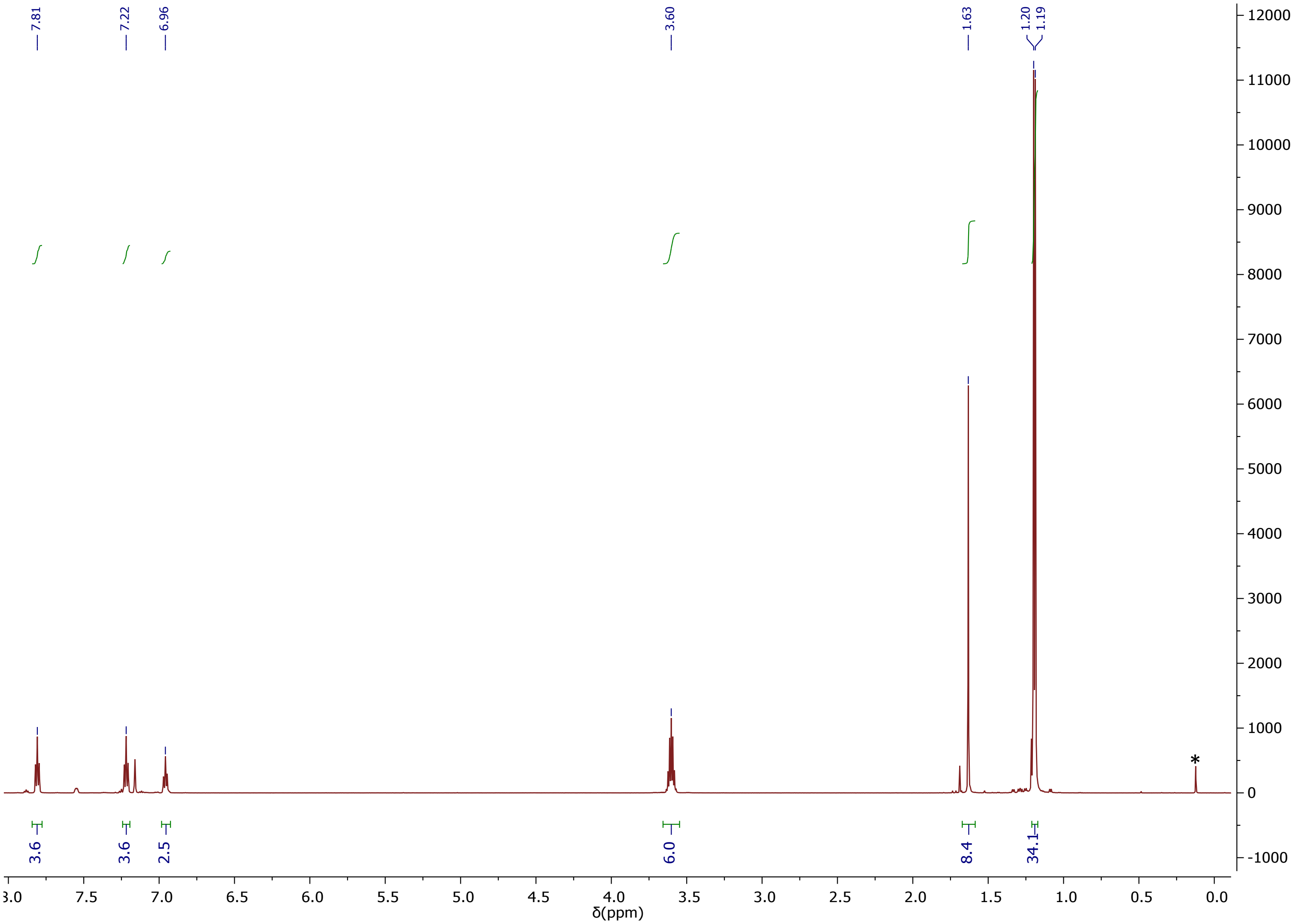

Figure S19. ^{1}H NMR spectrum of $Th(PPh_2)(BIMA)_3$ (**11b**) at 298 K in C_6D_6. *HMDSO

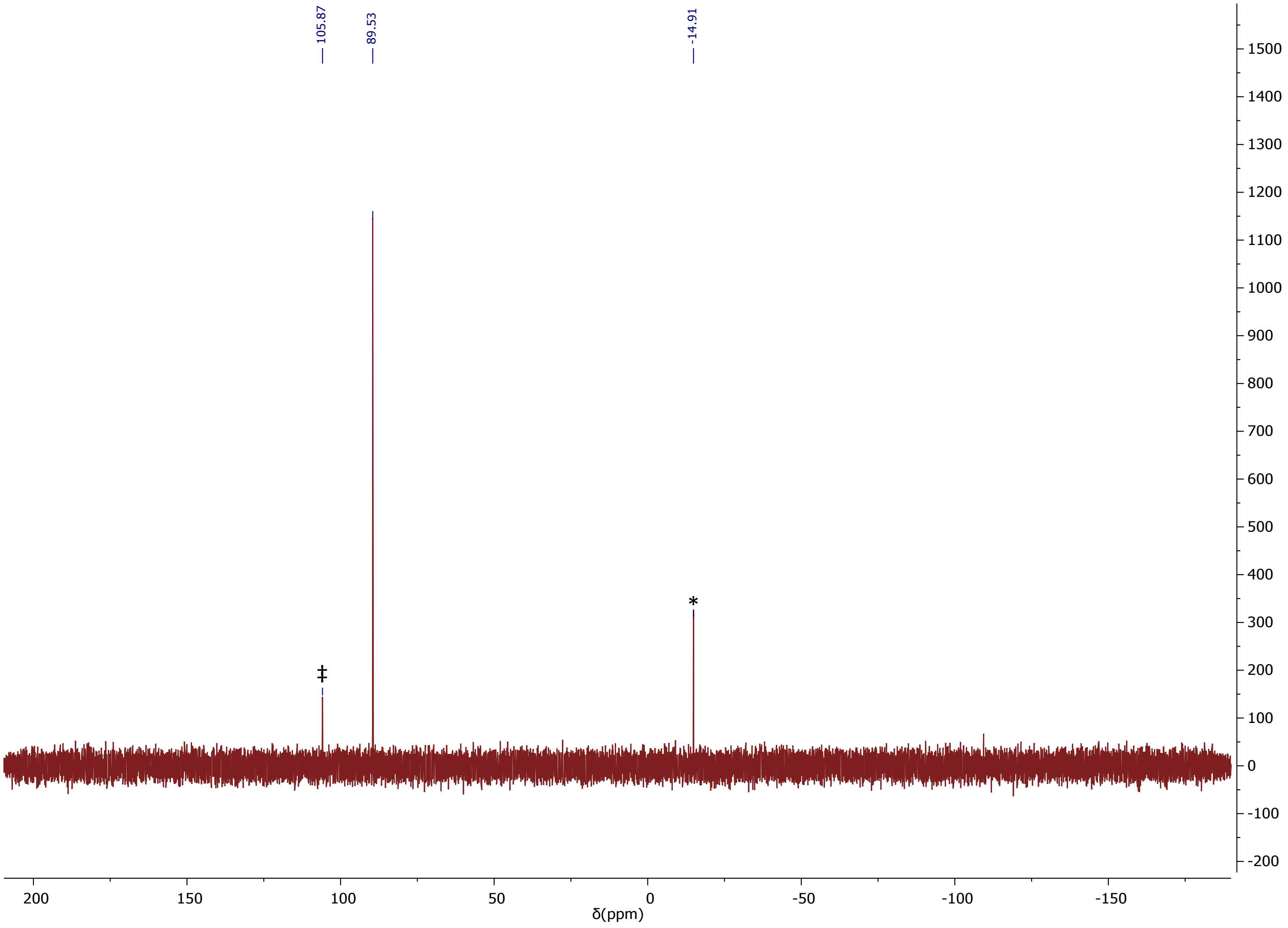

Figure S20. $^{31}P\{^{1}H\}$ NMR spectrum of $Th(PPh_2)(BIMA)_3$ (**11b**) at 298 K in C_6D_6. *Ph_2P-PPh_2. ‡unidentified phosphorus-containing species

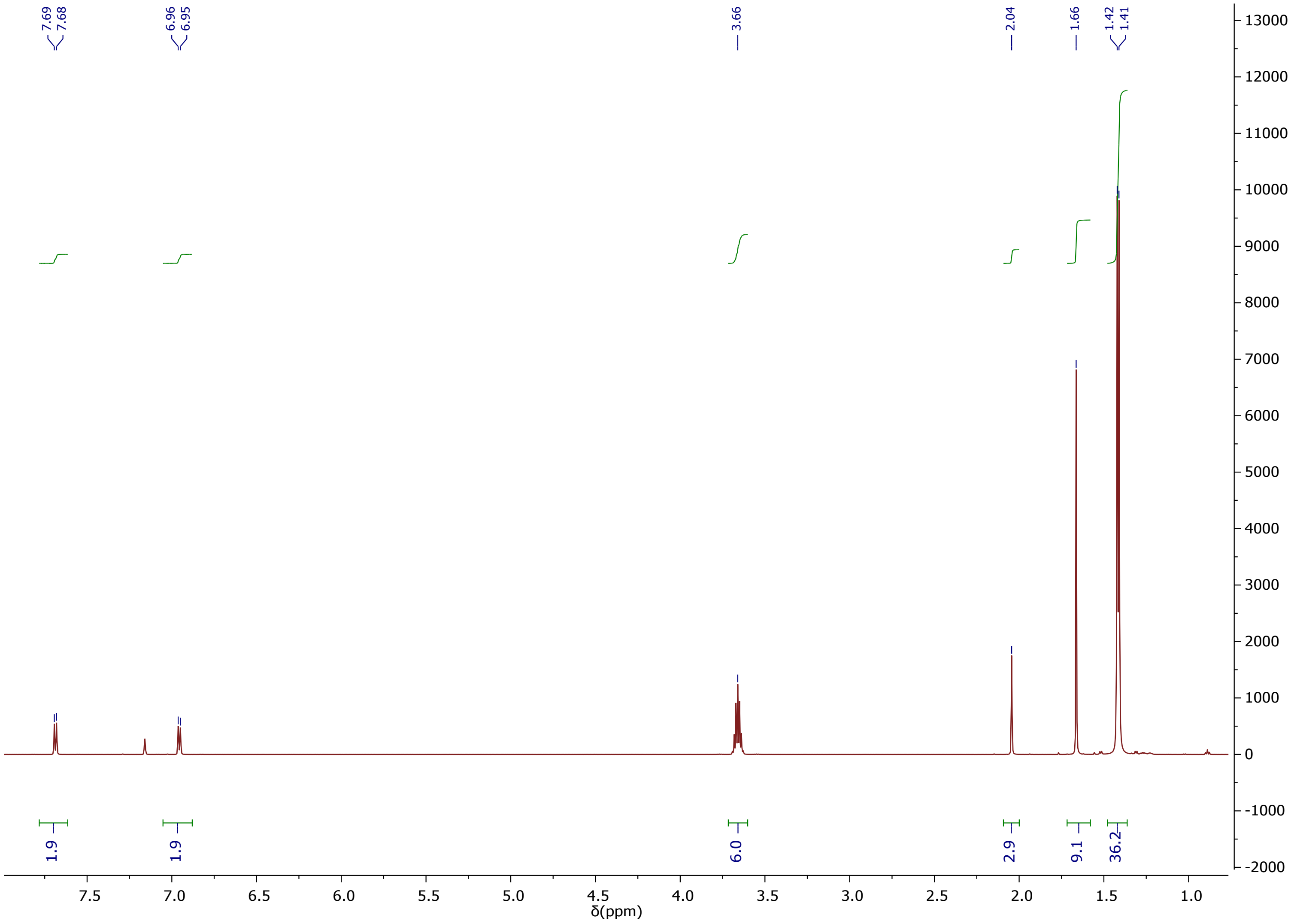

Figure S21. ^{1}H NMR spectrum of Th(C≡C-*p*-tolyl)(BIMA)$_3$ (**12**) at 298 K in C_6D_6

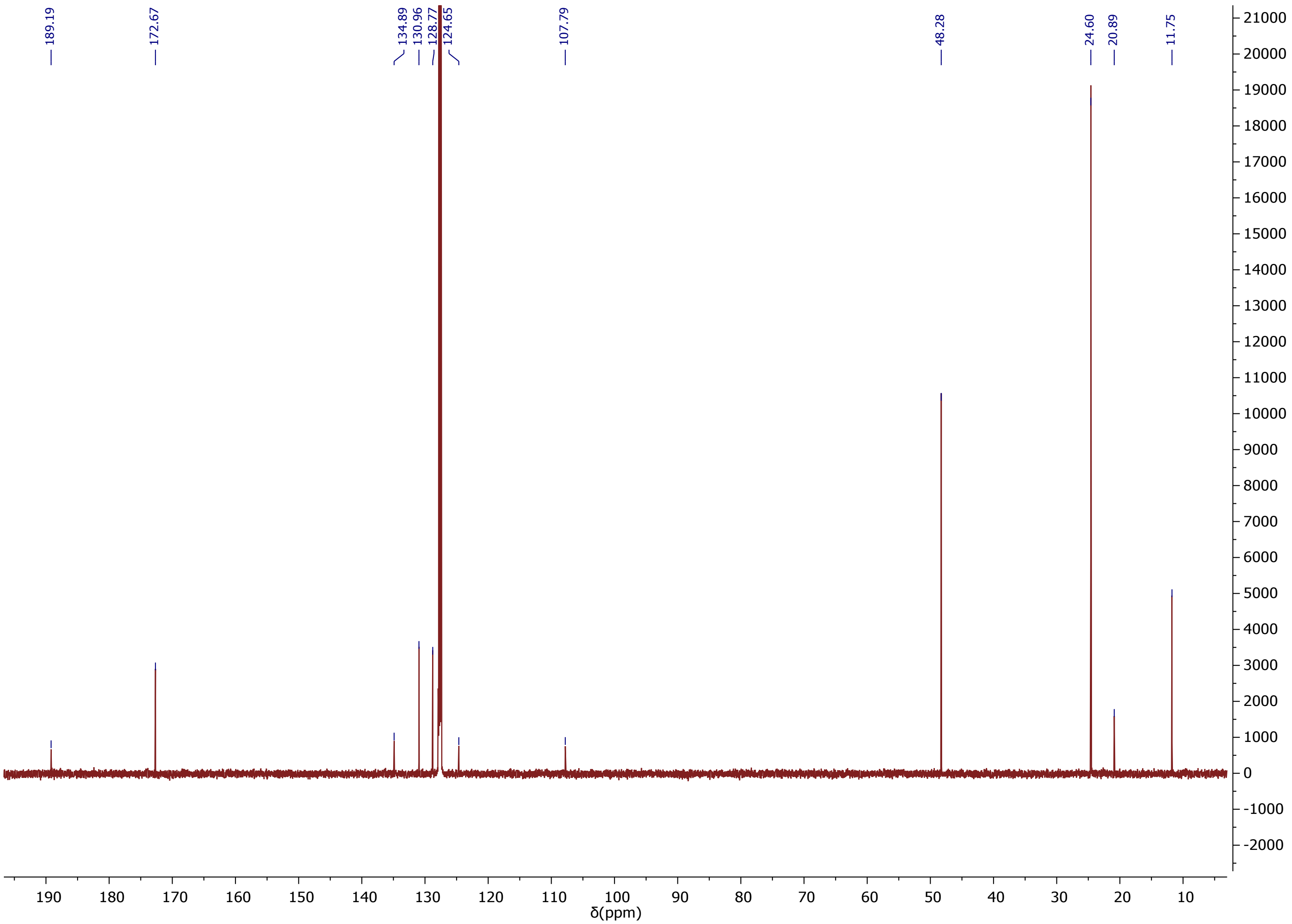

Figure S22. $^{13}C\{^{1}H\}$ NMR spectrum of Th(C≡C-*p*-tolyl)(BIMA)$_3$ (**12**) at 298 K in C_6D_6

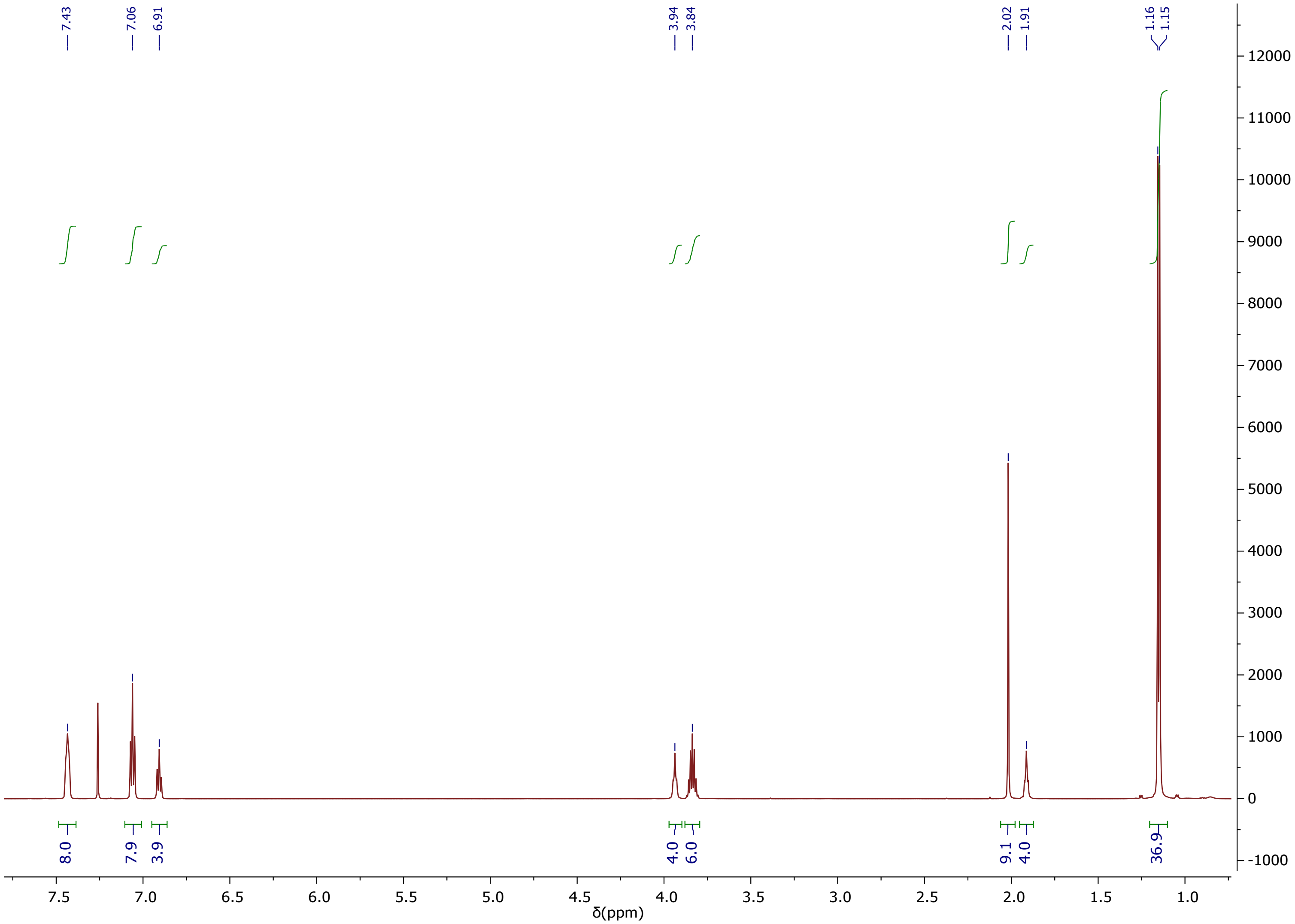

Figure S23. ^{1}H NMR spectrum of Th[(THF)(BIMA)$_3$][BPh$_4$] (**13**) at 298 K in $CDCl_3$

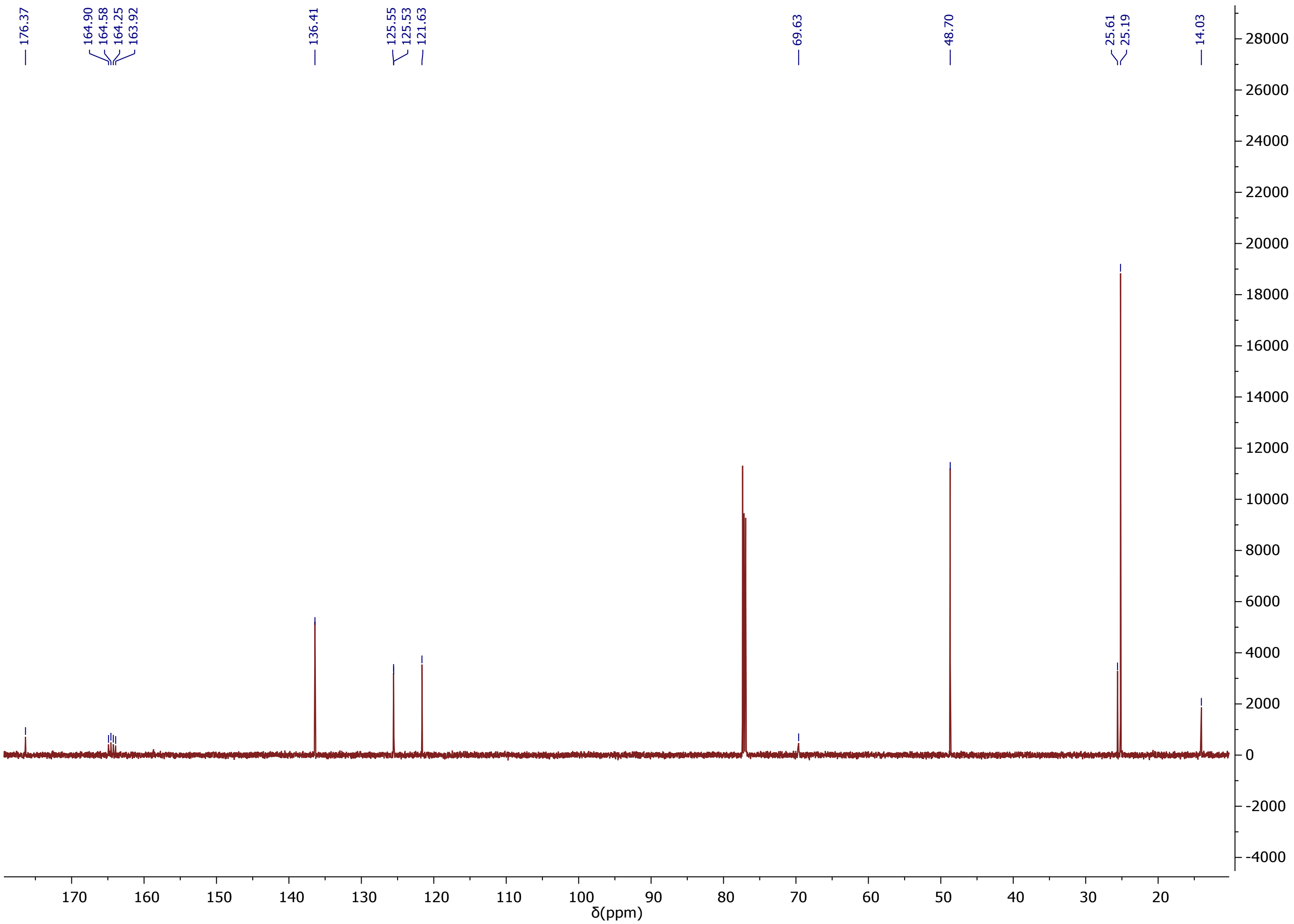

Figure S24. $^{13}C\{^{1}H\}$ NMR spectrum of Th[(THF)(BIMA)$_3$][BPh$_4$] (**13**) at 298 K in $CDCl_3$

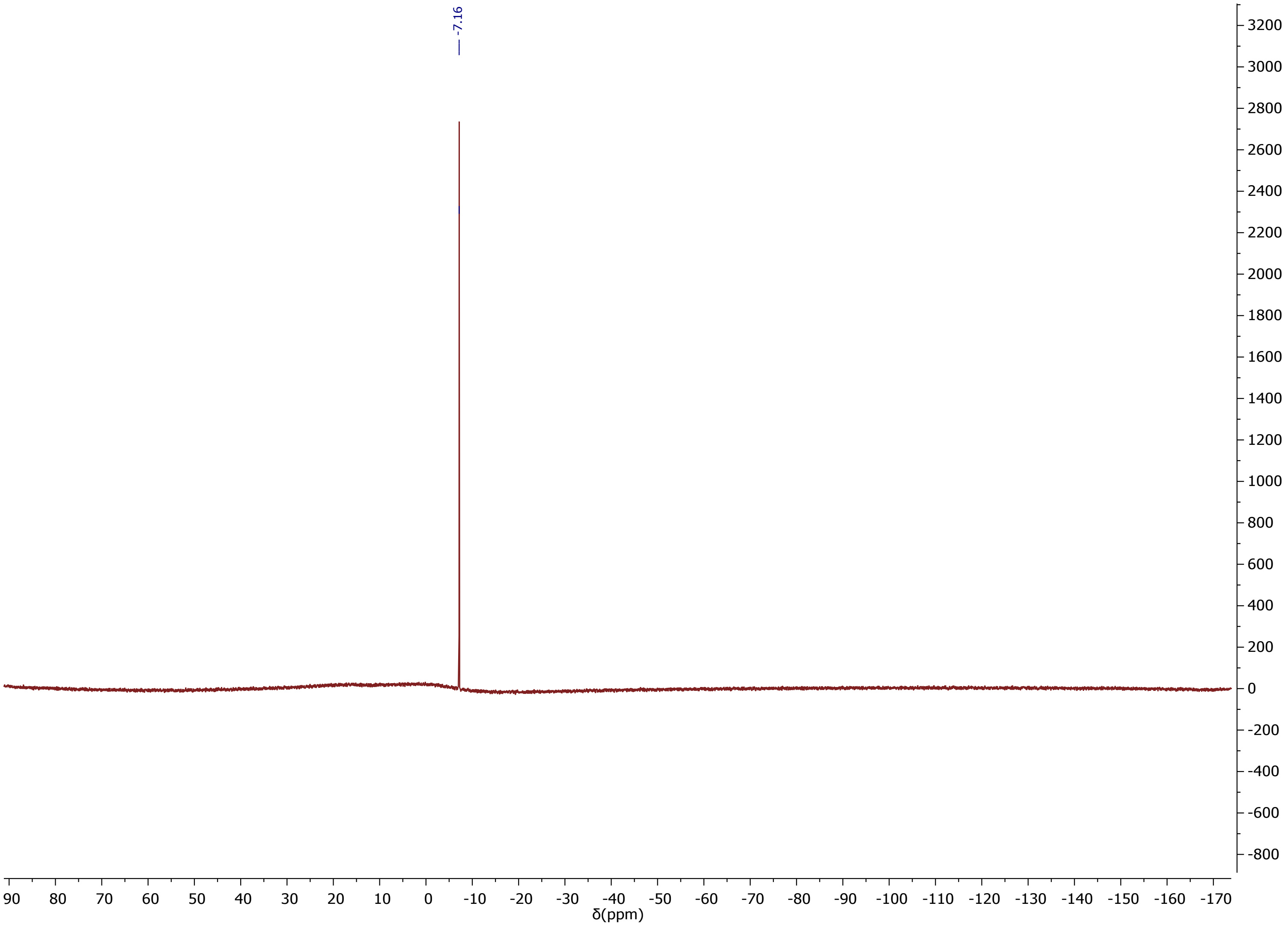

Figure S25. $^{11}B\{^{1}H\}$ NMR spectrum of Th[(THF)(BIMA)$_3$][BPh$_4$] (**13**) at 298 K in $CDCl_3$

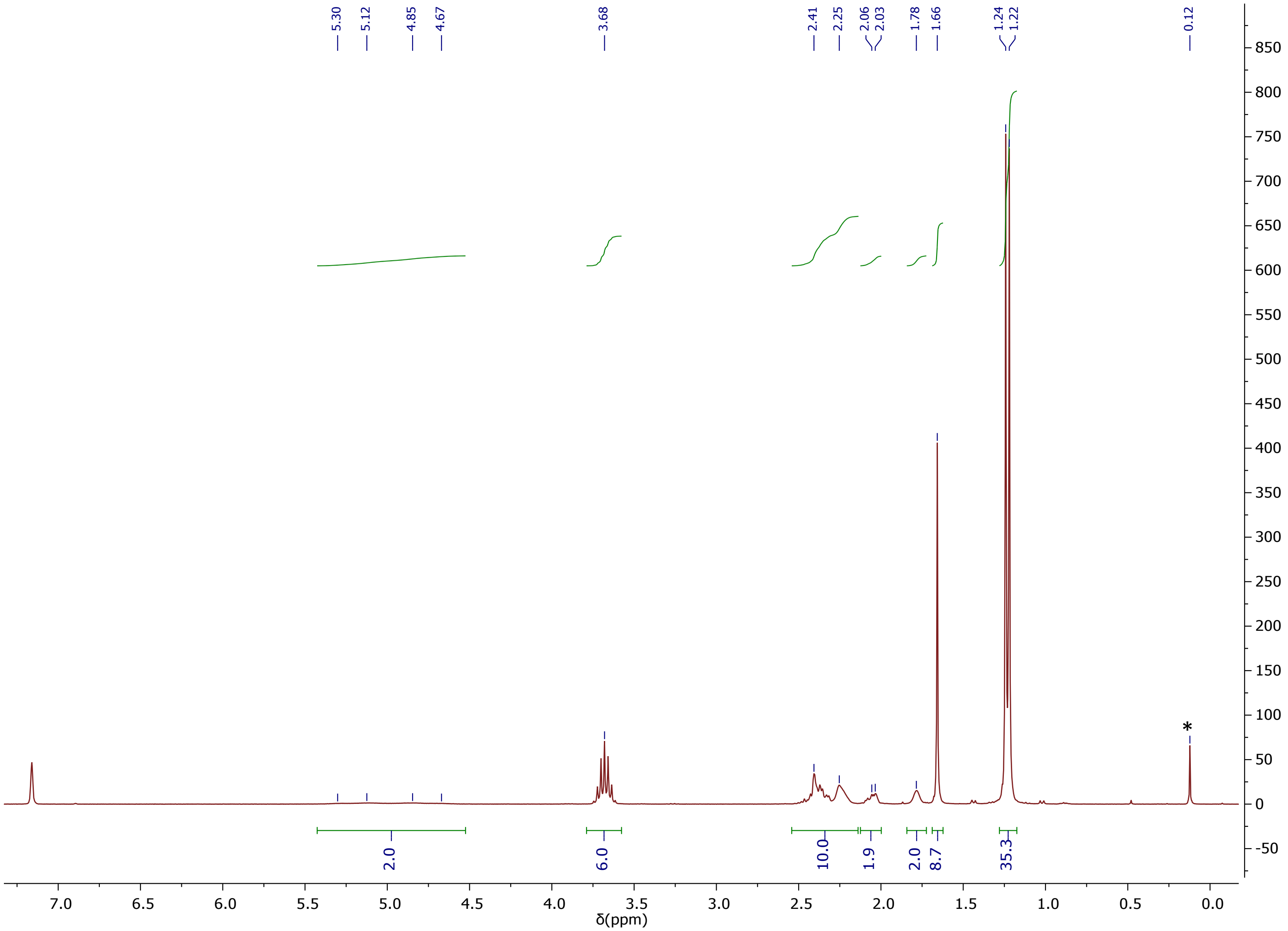

Figure S26. ^{1}H NMR spectrum of $(BIMA)_3Th(\mu\text{-}H)_2$(9-BBN) (**14**) at 298 K in C_6D_6. *HMDSO

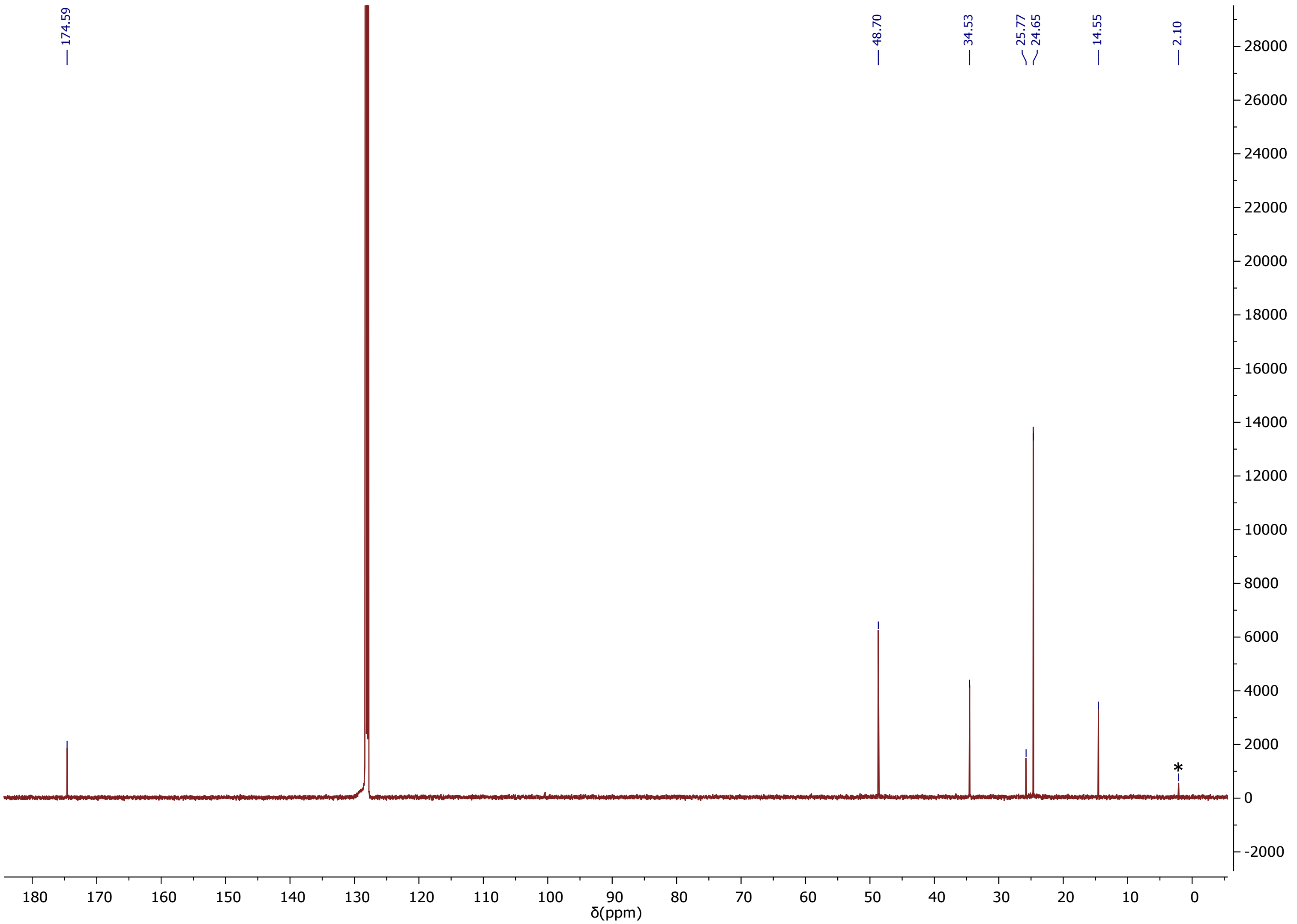

Figure S27. $^{13}C\{^1H\}$ NMR spectrum of $(BIMA)_3Th(\mu\text{-}H)_2$(9-BBN) (**14**) at 298 K in C_6D_6. *HMDSO

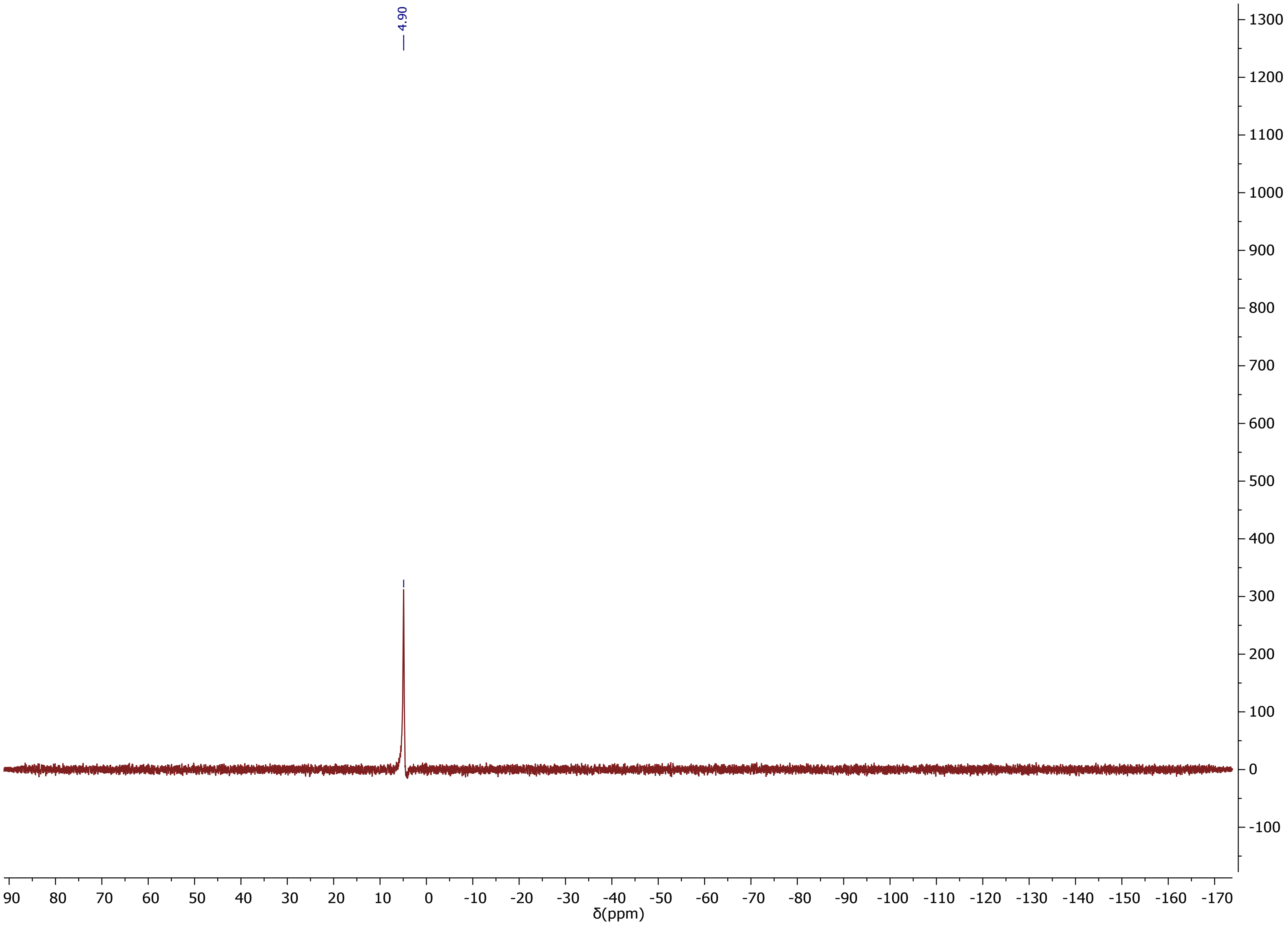

Figure S28. $^{11}B\{^{1}H\}$ NMR spectrum of $(BIMA)_3Th(\mu\text{-}H)_2(9\text{-}BBN)$ (**14**) at 298 K in C_6D_6

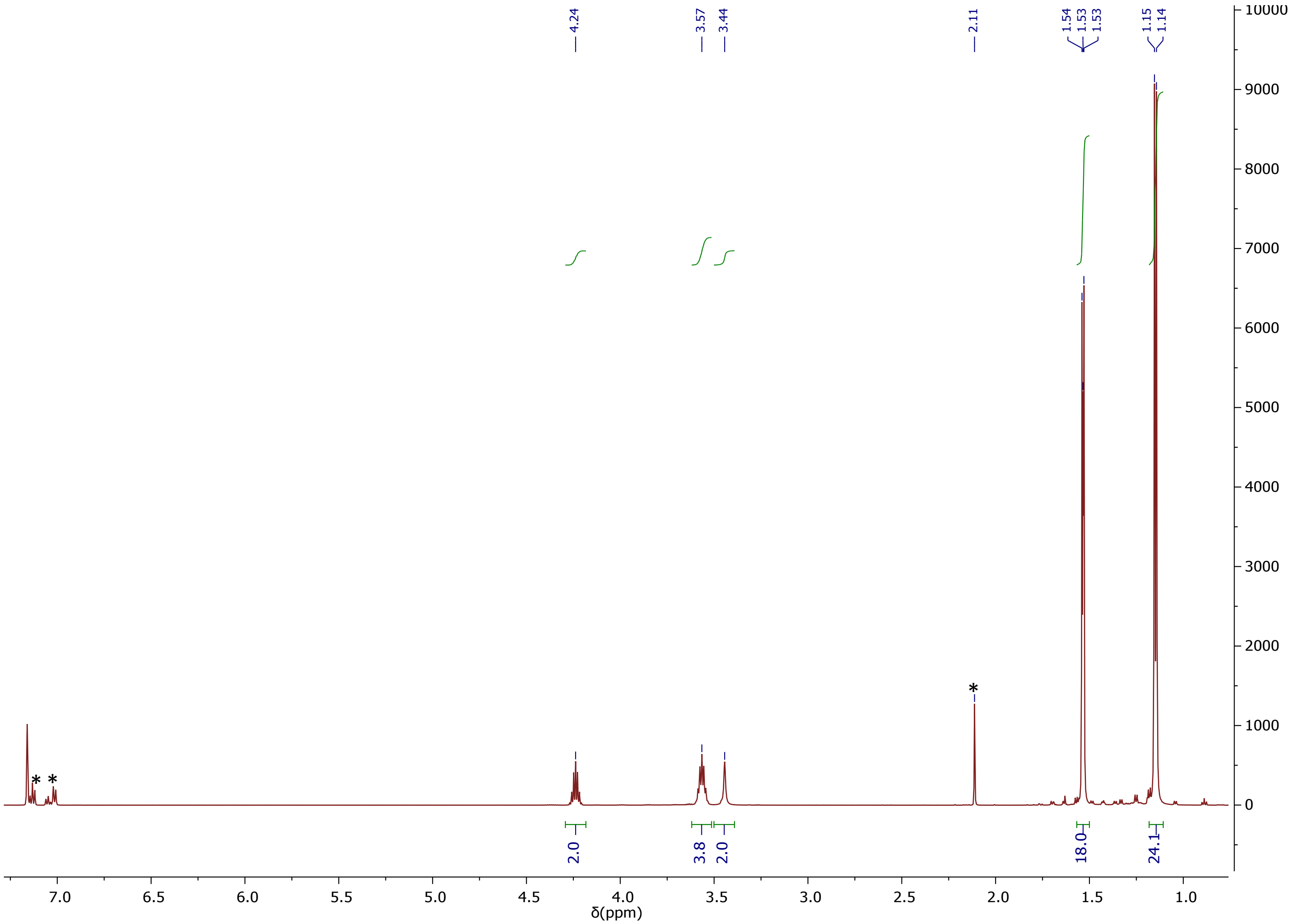

Figure S29. ^{1}H NMR spectrum of Th(BIMA)$_2$(BIMA*) (**15**) at 298 K in C_6D_6. *Toluene

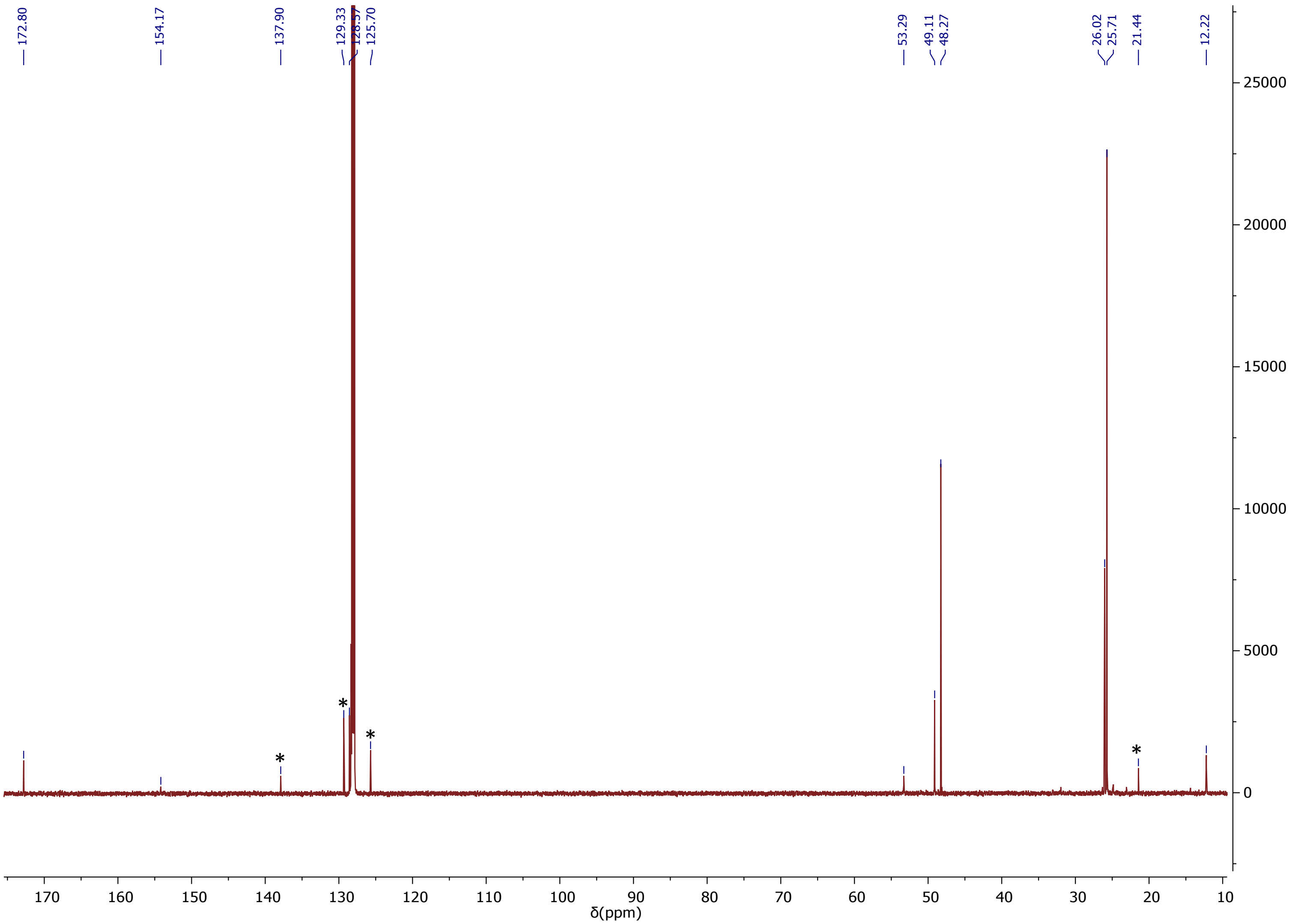

Figure S30. $^{13}C\{^{1}H\}$ NMR spectrum of Th(BIMA)$_2$(BIMA*) (**15**) at 298 K in C_6D_6. *Toluene

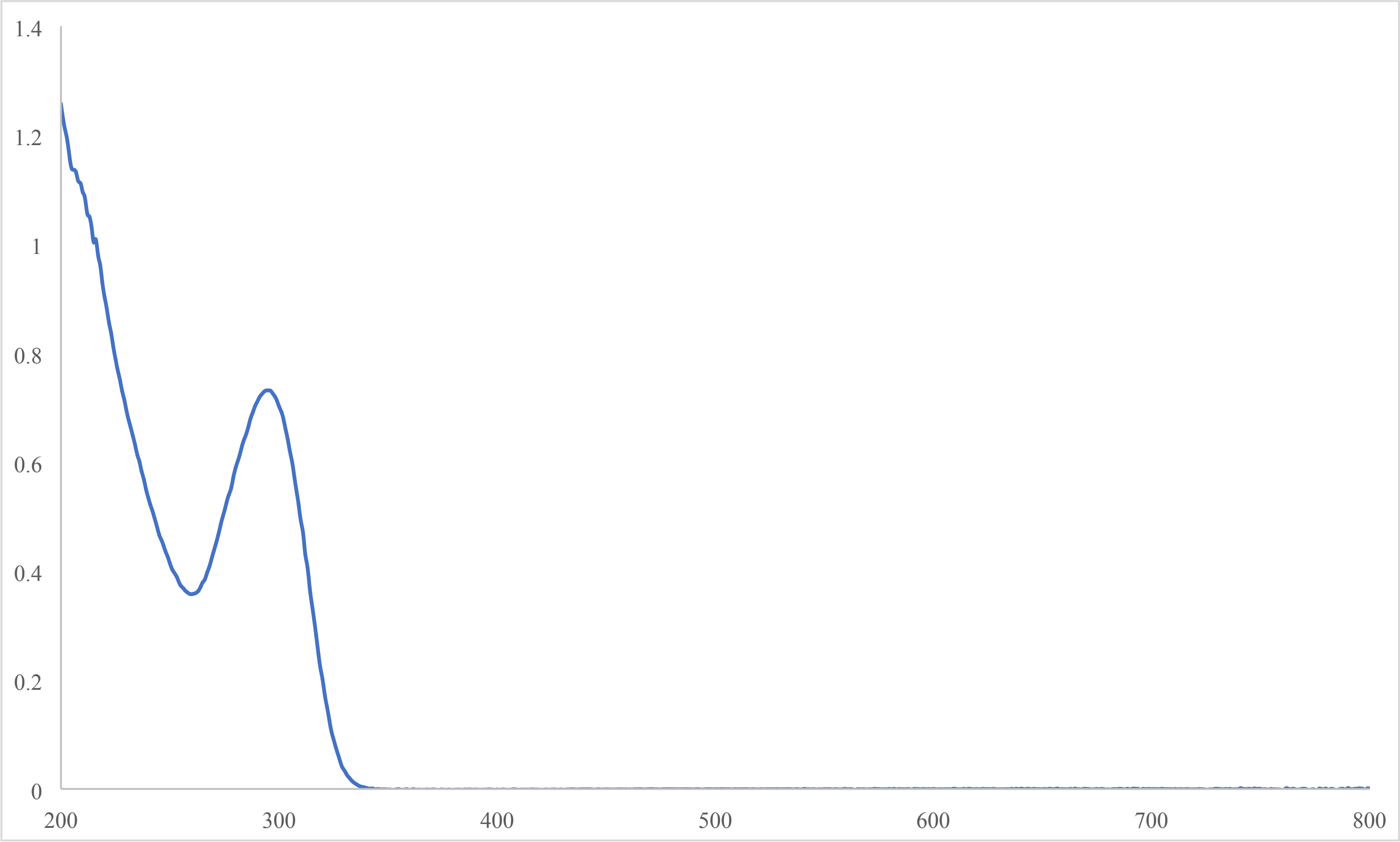

Figure S31. UV-Vis spectrum of $Th(CH_2SiMe_3)(BIMA)_3$ (**1**) in hexane

C. **X-ray Crystallography.** Single-crystal X-ray diffraction experiments were performed at the UC Berkeley CHEXRAY crystallographic facility. Measurements of all compounds were performed on a Bruker APEX-II area detector using Mo Kα radiation (λ = 0.71073 Å). Crystals were kept at 100(2) K throughout collection. Data collection was performed with Bruker APEX2 software (v. 2014.11). Data refinement and reduction were performed with Bruker SAINT (V8.34A). All structures were solved with SHELXT.53 Structures were refined with SHELXL-2014. Molecular graphics were computed with Mercury 3.10. All non-hydrogen atoms were refined anisotropically, and hydrogen atoms were either included at the geometrically calculated positions and refined using a riding model or located as Q peaks in the Fourier difference map.

	**2**	**3**	**4**	**5**	**6**	**7**	**11a**
Chemical formula	$C_{32}H_{68}N_8Th$	$C_{35}H_{69}N_9SiTh$	$C_{34}H_{67}N_7SiTh$	$C_{37}H_{71}N_7SiTh$	$C_{29}H_{62}N_6OSiTh$	$C_{30}H_{62}N_6O_2SiTh$	$C_{33}H_{63}N_6PTh$
Formula weight	796.99	876.12	834.07	874.15	770.97	798.98	806.92
Colour, habit	Colourless, block	Colourless, block	Colourless, block	Colourless, block	Colourless, block	Colourless, block	Yellow, block
Temperature (K)	100(2)	100(2)	100(2)	100(2)	100(2)	100(2)	100(2)
Crystal system	Triclinic	Monoclinic	Monoclinic	Monoclinic	Triclinic	Triclinic	Monoclinic
Space group	P-1	P 21/c	P 21/n	P 21/n	P-1	P-1	P 21/n
a (Å)	10.3830(4)	16.9023(9)	12.6373(9)	10.9839(5)	12.986(4)	9.4981(19)	22.5782(10)
b (Å)	11.3585(5)	11.4723(6)	17.8743(14)	20.9900(8)	16.992(5)	11.950(2)	13.2908(6)
c (Å)	17.4827(8)	21.7432(12)	17.1544(13)	18.2460(8)	17.337(4)	16.888(3)	25.4828(11)
α (°)	89.7768(18)	90	90	90	103.843(10)	90.32(3)	90
β (°)	89.4038(16)	95.127(3)	90.033(3)	93.4600(18)	97.592(10)	99.06(3)	100.332(2)
γ (°)	63.7604(15)	90	90	90	93.871(12)	102.29(3)	90
V ($Å^3$)	1849.26(14)	4199.3(4)	3874.9(5)	4199.0(3)	3662.2(17)	1848.0(7)	7522.9(6)
Z	2	4	4	4	2	2	4
Densitiy (Mg m^{-3})	1.431	1.386	1.430	1.383	1.398	1.436	1.425
F(000)	812	1784	1696	1784	1560	808	3264
Radiation Type	MoK_{α}	MoK_{α}	MoK_{α}	MoK_{α}	MoK_{α}	MoK_{α}	MoK_{α}
μ (mm^{-1})	4.063	3.613	3.910	3.612	4.132	4.099	3.613
Crystal size (mm^3)	0.18 x 0.20 x 0.20	0.05 x 0.05 x 0.10	0.10 x 0.22 x 0.25	0.15 x 0.20 x 0.20	0.15 x 0.24 x 0.29	0.05 x 0.10 x 0.13	0.15 x 0.22 x 0.40
Meas. Refl.	53843	142853	176871	60423	118651	148820	169945
Indep. Refl.	6800	15081	7927	7751	15074	5516	18721
R(int)	0.0291	0.0441	0.0315	0.0279	0.0399	0.0422	0.0414
Final R indices [I > 2σ(I)]	R = 0.0107 R_w = 0.0273	R = 0.0317 R_w = 0.0700	R = 0.0153 R_w = 0.0309	R = 0.0220 R_w = 0.0470	R = 0.0273 R_w = 0.0556	R = 0.0198 R_w = 0.0453	R = 0.0252 R_w = 0.0510
Goodness-of-fit	1.090	1.089	1.062	1.072	1.109	1.099	1.042
$\Delta\rho_{max}$, $\Delta\rho_{min}$ (e $Å^{-3}$)	0.499, -0.498	1.383, -1.210	1.145, -0.484	1.103, -1.137	3.302, -2.439	2.310, -0.709	1.986, -1.880
CCDC	1811106	1811107	1811108	1811109	1811110	1811111	1811112

	**12**	**13**	**14**	**15**
Chemical formula	$C_{33}H_{58}N_6Th$	$C_{56}H_{87}N_6O_2BTh$	$C_{32}H_{67}BN_6Th$	$C_{24}H_{50}N_6Th$
Formula weight	770.91	1119.16	778.76	654.74
Colour, habit	Colourless, block	Colourless, rod	Colourless, block	Colourless, block
Temperature (K)	100(2)	100(2)	100(2)	100(2)
Crystal system	Monoclinic	Monoclinic	Monoclinic	Triclinic
Space group	P 21/c	P 21/c	P 21/n	P-1
a (Å)	19.0249(9)	18.6971(7)	12.1573(13)	11.5082(5)
b (Å)	10.3942(5)	15.8036(5)	18.4711(18)	12.3194(5)
c (Å)	19.1330(9)	18.9111(6)	16.3766(18)	12.8462(5)
α (°)	90	90	90	61.8432(7)
β (°)	110.1048(18)	103.688(2)	90.215(5)	79.1570(8)
γ (°)	90	90	90	86.2752(8)
V ($Å^3$)	3553.0(3)	5429.2(3)	3677.5(7)	1576.47(11)
Z	4	4	4	4
Densitiy (Mg m^{-3})	1.441	1.369	1.407	1.468
F(000)	1552	2304	1584	694
Radiation Type	$MoK_α$	$MoK_α$	$MoK_α$	$MoK_α$
μ (mm^{-1})	4.226	4.129	4.083	4.753
Crystal size (mm^3)	0.08 x 0.08 x 0.12	0.02 x 0.02 x 0.05	0.05 x 0.05 x 0.05	0.10 x 0.10 x 0.10
Meas. Refl.	81894	138819	193336	47536
Indep. Refl.	6536	10015	6764	5773
R(int)	0.0265	0.0508	0.0484	0.0263
Final R indices [I > 2σ(I)]	R = 0.0148 R_w = 0.0320	R = 0.0204 R_w = 0.0407	R = 0.0207 R_w = 0.0452	R = 0.0207 R_w = 0.0488
Goodness-of-fit	1.102	1.068	1.103	1.117
$Δρ_{max}$, $Δρ_{min}$ (e $Å^{-3}$)	1.363, -0.615	1.452, -0.967	1.291, -0.758	2.661, -1.099
CCDC	1811113	1811114	1811115	1811116

D. References

1. T. Cantat, B. L. Scott, and J. L. Kiplinger, *Chem. Commun.,* 2010, **46**, 919-921.
2. S. Hao, S. Gambarotta, C. Bensimon, and J. J. H. Edema, *Inorg. Chim. Acta,* 1993, **213**, 65-74.
3. N. S. Settineri, M. E. Garner, and J. Arnold, *J. Am. Chem. Soc.,* 2017, **139**, 6261-6269.

4. J. Zhao, Z. Li, S. Yan, S. Xu, M. Wang, B. Fu, and Z. Zhang, *Org. Lett*., 2016, **18**, 1736−1739.
5. M. E. Garner and J. Arnold, *Organometallics,* 2017, **36**, 4511-4514.
